# Supplementary material for: De novo acyl carrier proteins display structure-independent modification and sequence novelty
Source: RSC Chem Biol. 2026 Apr 24;7(6):983–97. doi: 10.1039/d5cb00148j (PMC13159022; doi:10.1039/d5cb00148j)
Supplement: CB-007-D5CB00148J-s001 [file CB-007-D5CB00148J-s001.pdf]

Supplementary Information (SI) for RSC Chemical Biology.
This journal is © The Royal Society of Chemistry 2026

De novo acyl carrier proteins display structure-independent modification and sequence novelty

Michael A. Herrera[1]*, Grace K. King[2‡], Zoe Ozols[2‡], Gioele A. Tiburtini[3‡], Nicoletta Schiavo[3‡], Francesca Spyrakis[3]*, Louise K. Charkoudian[2]* and Dominic J. Campopiano[1]*

[1] School of Chemistry, The University of Edinburgh, Edinburgh EH9 3FJ, UK

[2] Department of Chemistry, Haverford College, Haverford, Pennsylvania 19041, USA

[3]Department of Drug Sciences and Technology, University of Turin, Turin 10125, Italy

‡ These authors contributed equally

* Corresponding author

Supplementary Information

Heading	Contents	Page
Additional Materials and Methods	Buffers and reagents<br>Plasmids, strains and proteins<br>LC/ESI-MS instrumentation, hardware and parameters.<br>Size-exclusion chromatography following *holo*→C12-acyl conversion.	2-3
Supplementary Tables	S1-S20	4-20
Supplementary Figures	Sequence analysis (Fig. S1-3)	21-22
	Purification data (Fig. S4-14)	23-29
	LC/ESI-MS (Fig. S15-37)	30-41
	Structural modelling and MDS (Fig. S38-S47)	32-48
	Additional CD data (Fig. S48)	49
	Additional sequence data (Fig. S49)	50
	Raw SDS-PAGE images	51
Plasmid Maps	pALGO and pCHALGO maps	53-64

Additional Materials and Methods

Buffers and reagents. A summary of all reagent stocks and rich media can be found in Table S1. CoASH, lauric acid, chloramphenicol and kanamycin sulfate were purchased from Sigma-Aldrich. Imidazole, DTT and IPTG were purchased from Fluorochem. Yeast extract and tryptone were purchased from Merck Life Sciences, and SOC recovery medium (for transformations) was purchased from New England Biolabs. VLB-Millar broth, VLB-agar and Autoinduction media was purchased from Formedium. All other chemicals and solvents were purchased from Fisher Scientific. Deionised water (dH_2O) for buffers, media and reagent stocks was obtained using a Sartorius purifier. All media was sterilised by autoclave prior to use. All buffers (see table S1) for protein purification were filtered (0.22 µm) and de-gassed under vacuum. For antibiotic selection, the working concentrations of carbenicillin, kanamycin and chloramphenicol were 100, 50 and 30 µg mL^{-1}, respectively.

Plasmids, strains and proteins. A summary of all expression plasmids used in this study can be found in Table S2. Plasmid pACYC-*sfp* was kindly provided by Ingenza Ltd. Plasmid pSGL-006 was kindly provided by the Prof. Michael D. Burkart research group. Plasmids pALGO-(013, 027, 040, 044, 055, 057, 059) and pCHALGO-(009, 012, 024, 044, 097) were synthesised and cloned by Genscript. Plasmids were propagated using NEB 5-alpha (New England Biolabs) cells and purified using Promega Wizard Plus SV miniprep kit. DNA sequencing was performed by GeneWiz. All purification hardware (including ÄKTA Start and ÄKTA Go FPLC purifiers) were purchased from Cytiva. SDS-PAGE was performed using pre-cast NuPAGE 4-12% Bis-Tris gels and NuPAGE MES running buffer purchased from Invitrogen and used according to manufacturer protocol. Protein gels were stained using InstantBlue Coomassie stain (Abcam). *Vh*AasS and *Ec*AcpS were provided as pre-purified aliquots courtesy of Dr. Gustavo Perez-Ortiz. Protein concentrations were determined using the BCA assay.

LC/ESI-MS instrumentation, hardware and parameters. This study was a joint expedition between labs in the University of Edinburgh (UK) and Haverford College (USA). Each lab reproduced the expression, purification and PTM of *Ec*AcpP and ALGO candidates ALGO-055 and ALGO-059, albeit mass analysis was performed using distinctive LC/ESI-MS setups as described below.

University of Edinburgh: Proteins were diluted to ~2-5 µM using LC-MS grade water and analysed using a Waters Synapt G2-Si Quadrupole/Time of Flight (TOF) HDMS coupled to a Waters Acquity Class I Plus UPLC, equipped with a Phenomenex Aeris C4 column (200 Å, 3.6 µm, 2.1 mm x 50 mm). Samples were analysed in positive ion mode. LC-MS grade water + 0.1% formic acid was used as Solvent A. ACN + 0.1% formic acid was used as Solvent B. Analytes were resolved using a fixed flow

rate (0.2 ml min-1) using the following method parameters: 95% A / 5% B (initial ratio, 1.5 min hold); 5% A / 95% B (3.5 min gradient, 6 min hold), 95% A / 5% B (0.1 min gradient, 0.9 min hold). Mass spectra were analysed and deconvoluted using MassLynx.

Haverford College: Proteins were diluted to ~10 µM using LCMS grade water and analysed using an Agilent Technologies InfinityLab G6125B LC/MS coupled with an Agilent 1260 Infinity II LC system, equipped with a Waters XBridge Protein BEH C4 reverse phase column (300 Å, 3.55 µm, 2.1 mm x 50 mm) heated to 45 °C. Samples were analysed via electrospray ionization mass spectrometry (ESI-MS) in positive ion mode. LC-MS grade water + 0.1% formic acid was used as Solvent A. ACN + 0.1% formic acid was used as Solvent B. Analytes were resolved using a fixed flow rate (0.2 ml min-1) using the following method parameters: 95% A / 5% B (initial ratio, 1 min hold); 5% A / 95% B (3.1 min gradient, 1.42 min hold), 95% A / 5% B (0.4 min gradient, 4.08 min hold). Mass spectra were deconvoluted using ESIprot online.

Size-exclusion chromatography following *holo*→C_{12}-acyl conversion. All buffers used in FPLC were filter sterilized (0.2 µm Nylon membrane filter) and degassed by vacuum, except for small buffer volumes used to wash syringe, needle, and injection port which were not degassed. FPLC was used to separate ACPs from other enzymes after *in vitro* reactions, in preparation for CD spectroscopy. FPLC was performed on an Akta Pure chromatography system (GE Healthcare) equipped with a Superdex 75 Increase 10/300 GL column (GE Healthcare). The column was equilibrated with 50 mM sodium phosphate buffer (pH 7.6) for ~ 90 minutes. A 500 µL sample loop was primed with 1 mL of 50 mM sodium phosphate buffer (pH 7.6), before ~500 µL of sample was injected into the loop. Buffer was run at a flow rate of 0.8 mL/min, with pressure maintained at 0.3-0.4 MPa, and UV absorbance monitored at 280 nm. Fractions were collected in 0.5 mL increments. Fractions containing significant UV absorbance (280 nm) were analyzed by LCMS, and pooled and concentrated by centrifugal filtration for further use. Samples were also confirmed by SDS-PAGE

Supplementary Tables (S1-20)

Table S1 Rich media and reagent stock solutions

Item	Contents
**VLB media**	Yeast extract (5 g L^{-1}), tryptone (10 g L^{-1}), NaCl (10 g L^{-1})
**VLB-Agar**	Yeast extract (5 g L^{-1}), tryptone (10 g L^{-1}), NaCl (10 g L^{-1}), Agar (15 g L^{-1})
**Autoinduction Media**	Yeast extract (5 g L^{-1}), tryptone (10 g L^{-1}), D-glucose (0.5 g L^{-1}), α-lactose (2 g L^{-1}), $(NH_4)_2SO_4$ (3.3 g L^{-1}), KH_2PO_4 (6.8 g L^{-1}), Na_2HPO_4 (7.1 g L^{-1}), $MgSO_4$ (0.15 g L^{-1}), trace elements (0.03 g L^{-1})
**Kanamycin (1000X)**	Kanamycin sulfate (50 mg mL^{-1}) in dH_2O
**Carbenicillin (1000X)**	Carbenicillin (100 mg mL^{-1}) in dH_2O
**Chloramphenicol (1000X)**	Chloramphenicol (30 mg mL^{-1}) in EtOH
**1 M IPTG (1000X)**	IPTG (238 mg mL^{-1}) in dH_2O
**DTT (100X)**	DTT (15.4 mg mL^{-1}) in dH_2O
**Lauric acid (1000X)**	Lauric acid (20.0 mg mL^{-1}) in DMSO
**CoASH (1000X)**	Coenzyme A sodium salt (76.6 mg mL^{-1}) in dH_2O
**ATP**	Adenosine triphosphate sodium salt (55.1 mg mL^{-1}) in dH_2O
**$MgCl_2$**	Magnesium chloride anhydrous (47.6 mg mL^{-1}) in dH_2O

Table S2 Plasmids and protein expression summary

Internal Name	Product	Vector	Resistance	Expression
pACYC-sfp	*Bs*Sfp	pACYC-Duet	CampR	0.1 mM IPTG, 16 °C, 18 hours
pET16b-aass	*Vh*AasS	pET16b	AmpR	0.5 mM IPTG, 37 °C, 4.5 hours
pSGL-005	*Ec*AcpP (C-term Histag)	pET23a	AmpR	Auto-induction, 30 °C, 18 hours
pALGO-013	ALGO-013 (C-term Histag)	pET28a	KanR	1 mM IPTG, 18 °C, 18 hours
pALGO-023	ALGO-013 (C-term Histag)	pET28a	KanR	1 mM IPTG, 18 °C, 18 hours
pALGO-040	ALGO-040 (C-term Histag)	pET28a	KanR	1 mM IPTG, 18 °C, 18 hours
pALGO-044	ALGO-044 (C-term Histag)	pET28a	KanR	1 mM IPTG, 18 °C, 18 hours
pALGO-055	ALGO-055 (C-term Histag)	pET28a	KanR	1 mM IPTG, 18 °C, 18 hours
pALGO-057	ALGO-057 (C-term Histag)	pET28a	KanR	1 mM IPTG, 18 °C, 18 hours
pALGO-059	ALGO-059 (C-term Histag)	pET28a	KanR	1 mM IPTG, 18 °C, 18 hours
pCHALGO-012	chALGO-012 (C-term Histag)	pET28a	KanR	1 mM IPTG, 18 °C, 18 hours
pCHALGO-012	chALGO-024 (C-term Histag)	pET28a	KanR	1 mM IPTG, 18 °C

Table S3 Seed AcpP sequences used for homologue retrieval.

Organism	Sequence
Escherichia coli	MSTIEERVKKIIGEQLGVKQEEVTNNASFVEDLGADSLDTVELVMALEEEFDTEIPDEEAEKITTVQAAIDYINGHQA
Pseudomonas putida	MSTIEERVKKIVAEQLGVKEEEVTPEKSFVDDLGADSLDTVELVMALEEEFETEIPDEEAEKITTVQAAIDYVNSHKA
Bacteroides fragilis	MSEIASRVKAIIVDKLGVEESEVTETASFTNDLGADSLDTVELIMEFEKEFGISIPDDQAEKIGTVQDAIAYIEEHAK
Sphingomonas paucimobilis	MSETADRVKKIVVEHLGVEADKVTEDASFIDDLGADSLDIVELVMAFEEEFGVEIPDDAAEKITTVKDAITYIDENKA
Salmonella enterica	MSTIEERVKKIIGEQLGVKQEEVTNNASFVEDLGADSLDTVELVMALEEEFDTEIPDEEAEKITTVQAAIDYINSHQA
Chlamydia trachomatis	MSLEDDVKAIIVDQLGVSPEDVKVDSSFIEDLNADSLDLTELIMTLEEKFAFEISEDDAEQLRTVGDVIKYIQEHQN
Helicobacter pylori	MSLFEDIQAVIAEQLNVDAAQVTPEAEFVKDLGADSLDVVELIMALEEKFGIEIPDEQAEKIVNVGDVVKYIEDNKLA
Legionella pneumophila	MSTVEERVRKIVVEQLGVKEEELKNDASFVDDLGADSLDTVELVMALEEEFETEIPDEKAEKITTIQEAIDYIESNLNKEEA
Vibrio cholerae	MSNIEERVKKIIVEQLGVDEAEVKNESSFVEDLGADSLDTVELVMALEEEFDTEIPDEEAEKITTVQAAIDYVTSNAQ
Neisseria meningitidis	MSNIEQQVKKIVAEQLGVNEADVKNESSFQDDLGADSLDTVELVMALEEAFGCEIPDEDAEKITTVQLAIDYINTHNG
Acinetobacter baumannii	MSTIEERVKKIVAEQLGVKEEEVTNSASFVEDLGADSLDTVELVMALEEEFETEIPDEKAEKITTVQEAIDYIVAHQQ
Campylobacter jejuni	MATFDDVKAVVVEQLGIDADAVKMESKIIEDLGADSLDVVELIMALEEKFEVEIPDSDAEKLIKIEDVVNYIDNLKK
Porphyromonas gingivalis	MSEVEKKVIDLVVDKLNVEASEVTREASFSNDLGADSLDTVELMMNFEKEFNMSIPDDQAQEIKTVGDAIDYIEKNLK
Caulobacter vibrioides	MSDILERVRKIVIEHLDADPEKVTEKASFIDDLGADSLDNVELVMAFEEEFDIEIPDDAAEHIQTVGDAVKFITEKTA
Bordetella parapertussis	MESIEQRVKKIVAEQLGVNEAEIKNESSFLDDLGADSLDMVELVMALEDEFETEIPDEEAEKITTVQQAVDYINSHGKQ
Burkholderia cepacia	MDNIEQRVKKIVAEQLGVAEAEIKTEASFVNDLGADSLDTVELVMALEDEFGMEIPDEEAEKITTVQQAIDYARANVKA
Yersinia pestis	MSTIEERVKKIIVEQLGVKEDEVKNSASFVEDLGADSLDTVELVMALEEEFDTEIPDEEAEKITTVQAAIDFINANQQ

Table S4 Descriptive statistics of sampled ALGO sequences (pI).

Factor	N	Mean	StDev	95% CI
r = 0.00	100	4.386	0.089	(4.369, 4.404)
r = 0.60	100	4.320	0.106	(4.299, 4.341)
r = 1.00	100	4.240	0.174	(4.205, 4.274)

Table S5 Descriptive statistics of sampled ALGO sequences (GRAVY).

Factor	N	Mean	StDev	95% CI
r = 0.00	100	-0.201	0.137	(-0.228, -0.173)
r = 0.60	100	-0.105	0.181	(-0.141, -0.069)
r = 1.00	100	-0.029	0.185	(-0.066, 0.007)

Table S6 Descriptive statistics of sampled ALGO sequences (pLDDT).

Factor	N	Mean	StDev	95% CI
r = 0.00	100	93.356	0.932	(93.170, 93.540)
r = 0.60	100	90.086	2.746	(89.541, 90.631)
r = 1.00	100	86.823	4.216	(85.986, 87.660)

Table S7 Welch's ANOVA (pI).

Source	DF Num	DF Den	F-Value	P-Value
Factor (pI)	2	187.826	31.773	1.30E-12

Table S8 Welch's ANOVA (GRAVY).

Source	DF Num	DF Den	F-Value	P-Value
Factor (GRAVY)	2	193.849	28.882	1.05E-11

Table S9 Welch's ANOVA (pLDDT).

Source	DF Num	DF Den	F-Value	P-Value
Factor (pLDDT)	2	150.882	166.258	<1.000 E-16

Table S10 Games-Howell simultaneous tests for differences of means (pI)

Difference of Levels	Difference of means	SE of Difference	95% CI	T-Value	Adjusted P-Value
r = 0.60 – r = 0.00	-0.066	0.014	(-0.099, -0.034)	-4.790	1.963E-05
r = 1.00 – r = 0.00	-0.146	0.020	(-0.193, -0.100)	-7.508	9.951E-06
r = 1.00 - r = 0.60	-0.080	0.020	(-0.129, -0.032)	-3.951	3.485E-04

Table S11 Games-Howell simultaneous tests for differences of means (GRAVY).

Difference of Levels	Difference of means	SE of Difference	95% CI	T-Value	Adjusted P-Value
r = 0.60 – r = 0.00	0.095	0.023	(0.042, 0.149)	4.191	1.362E-04
r = 1.00 – r = 0.00	0.171	0.023	(0.117, 0.226)	7.437	9.951E-06
r = 1.00 - r = 0.60	0.076	0.026	(0.015, 0.137)	2.933	1.045E-02

Table S12 Games-Howell simultaneous tests for differences of means (pLDDT).

Difference of Levels	Difference of means	SE of Difference	95% CI	T-Value	Adjusted P-Value
r = 0.60 – r = 0.00	-3.269	0.290	(-3.958, -2.581)	-11.28	9.951E-06
r = 1.00 – r = 0.00	-6.533	0.432	(-7.558, -5.507)	-15.13	9.951E-06
r = 1.00 - r = 0.60	-3.263	0.503	(-4.452, -2.075)	-6.49	9.953E-06

Table S13 ALGO sequences sampled for experimental testing.

Protein	Sequence	%ID (vs *Ec*AcpP)	GRAVY	pI	pLDDT
ALGO-013	MSTLPDKVKKIVADQLGVKEPKVKNHASFIQDLGA<br>DSLDTVELVMSMEEDFDVEIPDDTAEKISSVAQAID<br>YVTDHSA	64.10	-0.25	4.41	93.96
ALGO-023	MMSTNDKVEKIVLNELGVGETEVQLDAIFVDDLGA<br>DSLDSVELIMTLENSFDIQIPDDHAETITTVQFAVSY<br>ATAKSK	51.28	0.03	4.30	94.02
ALGO-040	MNDVSQRVQRVLGEQLGVVETEVVTSAAFVEDLG<br>ADSLDTVELVMSLEEAFNFNIPDEHAEDITQVQSAV<br>HYINEQSA	61.54	-0.08	4.20	94.72
ALGO-044	MSEIIKKVKKIVVQELGVQVNEVHESSAFVQDLGA<br>DSLDFVELIMSFEEQFHTAIPDEDAEKIITVAQAIHYI<br>NNNSG	57.69	-0.01	4.54	94.26
ALGO-055	MNPLEQRVKTIIVQELGVNEDVVINDASFVRDLGA<br>DSLDSVELVMALEKEFSIQIPDEQAEKIIQVSAAIDY<br>AEKAAK	61.54	0.00	4.46	93.52
ALGO-057	MNELFRKVQAIVVENLGVEGVKVTNESAFANDLGA<br>DSLDQVELLMAIEEQFDCDIPDEEAEDIITVADAILYI<br>GASSQ	53.85	0.13	4.33	93.91
ALGO-059	MSNLDQRVIDIIVQELGVPPKEVKSEASFIKDLGAD<br>SLDTVELIMSIEEDFNVEIPDEDAEHITTVASVLNYL<br>NEHSN	56.41	-0.15	4.27	93.74
^{ch}ALGO-009	MNPLDQRVITIIVQELGVPPKEVKSDASFVKDLGAD<br>SLDTVELIMSIEKEFSVQIPDEDAEHITTVSAVLDYA<br>NKHAN	57.69	-0.10	4.18	92.71
^{ch}ALGO-012	MSNLDQRVIDIIVQELGVNPDVVKSEASFIKDLGAD<br>SLDTVELIMSIEKDFSIQIPDEDAEHIIQVSAALNYAE<br>EHAN	51.28	-0.03	3.84	94.48
^{ch}ALGO-024	MSPLDQRVIDIIVQELGVNPDVVKSEASFIKDLGAD<br>SLDTVELIMALEKDFSIQIPDEDAEKIITVAAVIDYLN<br>EHAN	58.97	0.14	3.82	94.27
^{ch}ALGO-044	MNNLEQRVKDIIVQELGVNEDEVISDASFIKDLGAD<br>SLDTVELIMALEEDFNVEIPDEDAEKITTVAAAINYA<br>EKHSN	64.10	-0.235	3.78	93.56
^{ch}ALGO-097	MSNLEQRVKTIIVQELGVPEKVVINDASFVKDLGAD<br>SLDTVELIMSLEKEFNIEIPDEDAEHIITVASVINYAN<br>KASK	60.26	0.013	4.26	91.85

Table S14 LC/ESI-MS (*Ec*AcpP and ALGO sequences).

Protein	Empirical Formula	Theoretical MW / Da	Actual MW / Da	Comment
apo-*Ec*AcpP	$C_{420}H_{658}N_{114}O_{146}S_2$	9704.50	9573.39 ± 0.09	-Met_1
holo-*Ec*AcpP	$C_{431}H_{679}N_{116}O_{152}S_3P$	10044.83	9913.78 ± 0.04	-Met_1
C_{12}-*Ec*AcpP	$C_{443}H_{702}N_{116}O_{153}S_3P$	10228.14	10096.08 ± 0.14	-Met_1
apo-ALGO-013	$C_{418}H_{657}N_{113}O_{139}S_3$	9585.53	9454.33 ± 0.02	-Met_1
apo-ALGO-040	$C_{417}H_{642}N_{118}O_{142}S_2$	9644.37	9512.88 ± 0.25<br>9644.40 ± 0.18	Partial loss of Met_1
apo-ALGO-055	$C_{425}H_{679}N_{117}O_{136}S_2$	9667.74	9667.43 ± 0.00	
holo-ALGO-055	$C_{436}H_{700}N_{119}O_{142}S_3P$	10008.08	10007.91 ± 0.12	
C_{12}-ALGO-055	$C_{448}H_{722}N_{119}O_{143}S_3P$	10190.38	10190.28 ± 0.00	
apo-ALGO-059	$C_{426}H_{665}N_{115}O_{143}S_2$	9749.63	9618.46 ± 0.12	-Met_1
holo-ALGO-059	$C_{437}H_{686}N_{117}O_{149}S_3P$	10089.96	9958.44 ± 0.13	-Met_1
C_{12}-ALGO-059	$C_{449}H_{708}N_{117}O_{150}S_3P$	10272.26	10140.79 ± 0.12	-Met_1
apo-^{ch}ALGO-012	$C_{423}H_{662}N_{116}O_{140}S_2$	9676.64	9545.40 ± 0.02	-Met_1
holo- ^{ch}ALGO-012	$C_{434}H_{683}N_{118}O_{146}S_3P$	10016.91	9885.66 ± 0.01	-Met_1
C_{12}- ^{ch}ALGO-012	$C_{446}H_{704}N_{118}O_{147}S_3P$	10198.21	10067.91 ± 0.02	-Met_1
apo- ^{ch}ALGO-024	$C_{427}H_{675}N_{113}O_{137}S_2$	9647.76	9647.70 ± 0.02	
holo- ^{ch}ALGO-024	$C_{438}H_{696}N_{115}O_{143}S_3P$	9988.04	9987.96 ± 0.02	
C_{12}- ^{ch}ALGO-024	$C_{450}H_{718}N_{115}O_{144}S_3P$	10170.34	10170.16 ± 0.04	

Table S15 Disordered-ordered classification of proteins studied by CD spectroscopy. Values were computed using BeStSel server using mean residue ellipticity as input.

Protein	PTM	197 nm	206 nm	233 nm	Prediction
*Ec*AcpP	*apo-*	3.89	-3.33	-1.52	ordered
	holo-	3.77	-3.11	-1.23	ordered
	C_{12}-	5.79	-4.16	-1.74	ordered
ALGO-055	*apo-*	-4.01	-5.19	-1.58	disordered
	holo-	-2.25	-2.73	-0.73	disordered
	C_{12}-	1.37	-4.16	-1.66	ordered
ALGO-059	*apo-*	-4.46	-4.38	-1.11	disordered
	holo-	-2.74	-2.66	-0.66	disordered
	C_{12}-	1.38	-4.61	-1.62	ordered

Table S16 Estimated percent helical content of proteins studied by CD spectroscopy across PTM states. Values were computed using BeStSel server using mean residue ellipticity as input.

Protein	***apo-***	***holo-***	**C_{12}-**
*Ec*AcpP	28.1	22.2	35.0
ALGO-055	15.6	5.8	21.1
ALGO-059	12.2	4.0	28.2

Table S17 Positional analysis of ALGO-055 with comparison to *Ec*AcpP.

Position	Residue	vdW / Å	H_{KD}	pI	Rarity (vs input MSA)	Equivalent (*Ec*AcpP)	BLOSUM62 (vs. *Ec*AcpP)	Grantham Distance (vs. *Ec*AcpP)
1	M	162.9	1.90	5.74	0.749	M	5	0
2	N	122.4	-3.50	5.41	0.012	S	1	46
3	P	121.6	-1.60	6.30	0.001	T	-1	38
4	L	163.1	3.80	5.98	0.072	I	2	5
5	E	138.8	-3.50	3.22	0.585	E	5	0
6	Q	146.9	-3.50	5.65	0.162	E	2	29
7	R	190.3	-4.50	10.76	0.728	R	5	0
8	V	138.2	4.20	5.96	0.933	V	4	0
9	K	165.1	-3.90	9.74	0.814	K	5	0
10	T	119.6	-0.70	5.60	0.007	K	-1	78
11	I	163.0	4.50	6.02	0.898	I	4	0
12	I	163.0	4.50	6.02	0.394	I	4	0
13	V	138.2	4.20	5.96	0.545	G	-3	109
14	Q	146.9	-3.50	5.65	0.004	E	2	29
15	E	138.8	-3.50	3.22	0.004	Q	2	29
16	L	163.1	3.80	5.98	0.996	L	4	0
17	G	63.8	-0.40	5.97	0.876	G	6	0
18	V	138.2	4.20	5.96	0.915	V	4	0
19	N	122.4	-3.50	5.41	0.142	K	0	94
20	E	138.8	-3.50	3.22	0.655	Q	2	29
21	D	114.4	-3.50	2.77	0.234	E	2	45
22	V	138.2	4.20	5.96	0.001	E	-2	121
23	V	138.2	4.20	5.96	0.867	V	4	0
24	I	163.0	4.50	6.02	0.012	T	-1	89
25	N	122.4	-3.50	5.41	0.376	N	6	0
26	D	114.4	-3.50	2.77	0.100	N	1	23
27	A	89.3	1.80	6.00	0.652	A	4	0
28	S	89.0	-0.80	5.68	0.838	S	4	0
29	F	190.8	2.80	5.48	0.983	F	6	0
30	V	138.2	4.20	5.96	0.619	V	4	0
31	R	190.3	-4.50	10.76	0.001	E	0	54
32	D	114.4	-3.50	2.77	0.999	D	6	0
33	L	163.1	3.80	5.98	0.999	L	4	0
34	G	63.8	-0.40	5.97	0.957	G	6	0
35	A	89.3	1.80	6.00	0.984	A	4	0
36	D	114.4	-3.50	2.77	0.999	D	6	0
37	S	89.0	-0.80	5.68	0.999	S	4	0
38	L	163.1	3.80	5.98	0.999	L	4	0
39	D	114.4	-3.50	2.77	1.000	D	6	0
40	S	89.0	-0.80	5.68	0.001	T	1	58
41	V	138.2	4.20	5.96	0.971	V	4	0
42	E	138.8	-3.50	3.22	1.000	E	5	0
43	L	163.1	3.80	5.98	0.992	L	4	0
44	V	138.2	4.20	5.96	0.784	V	4	0
45	M	162.9	1.90	5.74	0.998	M	5	0

46	A	89.3	1.80	6.00	0.848	A	4	0
47	L	163.1	3.80	5.98	0.695	L	4	0
48	E	138.8	-3.50	3.22	0.998	E	5	0
49	K	165.1	-3.90	9.74	0.113	E	1	56
50	E	138.8	-3.50	3.22	0.850	E	5	0
51	F	190.8	2.80	5.48	0.996	F	6	0
52	S	89.0	-0.80	5.68	0.025	D	0	65
53	I	163.0	4.50	6.02	0.258	T	-1	89
54	Q	146.9	-3.50	5.65	0.005	E	2	29
55	I	163.0	4.50	6.02	0.987	I	4	0
56	P	121.6	-1.60	6.30	0.950	P	7	0
57	D	114.4	-3.50	2.77	0.973	D	6	0
58	E	138.8	-3.50	3.22	0.696	E	5	0
59	Q	146.9	-3.50	5.65	0.153	E	2	29
60	A	89.3	1.80	6.00	0.991	A	4	0
61	E	138.8	-3.50	3.22	0.984	E	5	0
62	K	165.1	-3.90	9.74	0.841	K	5	0
63	I	163.0	4.50	6.02	0.910	I	4	0
64	I	163.0	4.50	6.02	0.018	T	-1	89
65	Q	146.9	-3.50	5.65	0.001	T	-1	42
66	V	138.2	4.20	5.96	0.948	V	4	0
67	S	89.0	-0.80	5.68	0.009	Q	0	68
68	A	89.3	1.80	6.00	0.142	A	4	0
69	A	89.3	1.80	6.00	0.869	A	4	0
70	I	163.0	4.50	6.02	0.669	I	4	0
71	D	114.4	-3.50	2.77	0.559	D	6	0
72	Y	194.6	-1.30	5.66	0.832	Y	7	0
73	A	89.3	1.80	6.00	0.018	I	-1	94
74	E	138.8	-3.50	3.22	0.309	N	0	42
75	K	165.1	-3.90	9.74	0.108	G	-2	127
76	A	89.3	1.80	6.00	0.030	H	-2	86
77	A	89.3	1.80	6.00	0.079	Q	-1	91
78	K	165.1	-3.90	9.74	0.137	A	-1	106

Table S18 Positional analysis of ALGO-059 with comparison to *Ec*AcpP.

Position	Residue	vdW / Å	H_{KD}	pI	Rarity (vs input MSA)	Equivalent (*Ec*AcpP)	BLOSUM62 (vs. *Ec*AcpP)	Grantham Distance (vs. *Ec*AcpP)
1	M	162.9	1.90	5.74	0.749	M	5	0
2	S	89.0	-0.80	5.68	0.624	S	4	0
3	N	122.4	-3.50	5.41	0.206	T	0	65
4	L	163.1	3.80	5.98	0.072	I	2	5
5	D	114.4	-3.50	2.77	0.016	E	2	45
6	Q	146.9	-3.50	5.65	0.162	E	2	29
7	R	190.3	-4.50	10.76	0.728	R	5	0
8	V	138.2	4.20	5.96	0.933	V	4	0
9	I	163.0	4.50	6.02	0.032	K	-3	102
10	D	114.4	-3.50	2.77	0.050	K	-1	101
11	I	163.0	4.50	6.02	0.898	I	4	0
12	I	163.0	4.50	6.02	0.394	I	4	0
13	V	138.2	4.20	5.96	0.545	G	-3	109
14	Q	146.9	-3.50	5.65	0.004	E	2	29
15	E	138.8	-3.50	3.22	0.004	Q	2	29
16	L	163.1	3.80	5.98	0.996	L	4	0
17	G	63.8	-0.40	5.97	0.876	G	6	0
18	V	138.2	4.20	5.96	0.915	V	4	0
19	P	121.6	-1.60	6.30	0.006	K	-1	103
20	P	121.6	-1.60	6.30	0.084	Q	-1	76
21	K	165.1	-3.90	9.74	0.013	E	1	56
22	E	138.8	-3.50	3.22	0.611	E	5	0
23	V	138.2	4.20	5.96	0.867	V	4	0
24	K	165.1	-3.90	9.74	0.347	T	-1	78
25	S	89.0	-0.80	5.68	0.047	N	1	46
26	E	138.8	-3.50	3.22	0.568	N	0	42
27	A	89.3	1.80	6.00	0.652	A	4	0
28	S	89.0	-0.80	5.68	0.838	S	4	0
29	F	190.8	2.80	5.48	0.983	F	6	0
30	I	163.0	4.50	6.02	0.241	V	3	29
31	K	165.1	-3.90	9.74	0.032	E	1	56
32	D	114.4	-3.50	2.77	0.999	D	6	0
33	L	163.1	3.80	5.98	0.999	L	4	0
34	G	63.8	-0.40	5.97	0.957	G	6	0
35	A	89.3	1.80	6.00	0.984	A	4	0
36	D	114.4	-3.50	2.77	0.999	D	6	0
37	S	89.0	-0.80	5.68	0.999	S	4	0
38	L	163.1	3.80	5.98	0.999	L	4	0
39	D	114.4	-3.50	2.77	1.000	D	6	0
40	T	119.6	-0.70	5.60	0.784	T	5	0
41	V	138.2	4.20	5.96	0.971	V	4	0
42	E	138.8	-3.50	3.22	1.000	E	5	0
43	L	163.1	3.80	5.98	0.992	L	4	0
44	I	163.0	4.50	6.02	0.209	V	3	29

45	M	162.9	1.90	5.74	0.998	M	5	0
46	S	89.0	-0.80	5.68	0.007	A	1	99
47	I	163.0	4.50	6.02	0.001	L	2	5
48	E	138.8	-3.50	3.22	0.998	E	5	0
49	E	138.8	-3.50	3.22	0.845	E	5	0
50	D	114.4	-3.50	2.77	0.001	E	2	45
51	F	190.8	2.80	5.48	0.996	F	6	0
52	N	122.4	-3.50	5.41	0.112	D	1	23
53	V	138.2	4.20	5.96	0.119	T	0	69
54	E	138.8	-3.50	3.22	0.869	E	5	0
55	I	163.0	4.50	6.02	0.987	I	4	0
56	P	121.6	-1.60	6.30	0.950	P	7	0
57	D	114.4	-3.50	2.77	0.973	D	6	0
58	E	138.8	-3.50	3.22	0.696	E	5	0
59	D	114.4	-3.50	2.77	0.186	E	2	45
60	A	89.3	1.80	6.00	0.991	A	4	0
61	E	138.8	-3.50	3.22	0.984	E	5	0
62	H	157.5	-3.20	7.59	0.012	K	-1	32
63	I	163.0	4.50	6.02	0.910	I	4	0
64	T	119.6	-0.70	5.60	0.548	T	5	0
65	T	119.6	-0.70	5.60	0.892	T	5	0
66	V	138.2	4.20	5.96	0.948	V	4	0
67	A	89.3	1.80	6.00	0.010	Q	-1	91
68	S	89.0	-0.80	5.68	0.032	A	1	99
69	V	138.2	4.20	5.96	0.124	A	0	64
70	L	163.1	3.80	5.98	0.010	I	2	5
71	N	122.4	-3.50	5.41	0.057	D	1	23
72	Y	194.6	-1.30	5.66	0.832	Y	7	0
73	L	163.1	3.80	5.98	0.056	I	2	5
74	N	122.4	-3.50	5.41	0.250	N	6	0
75	E	138.8	-3.50	3.22	0.164	G	-2	98
76	H	157.5	-3.20	7.59	0.342	H	8	0
77	S	89.0	-0.80	5.68	0.050	Q	0	68
78	N	122.4	-3.50	5.41	0.009	A	-2	111

Table S19 Positional comparison between ALGO-055 and ALGO-059

Position	ALGO-055	ALGO-059	BLOSUM62	Grantham Distance
1	M	M	5	0
2	N	S	1	46
3	P	N	-2	91
4	L	L	4	0
5	E	D	2	45
6	Q	Q	5	0
7	R	R	5	0
8	V	V	4	0
9	K	I	-3	102
10	T	D	-1	85
11	I	I	4	0
12	I	I	4	0
13	V	V	4	0
14	Q	Q	5	0
15	E	E	5	0
16	L	L	4	0
17	G	G	6	0
18	V	V	4	0
19	N	P	-2	91
20	E	P	-1	93
21	D	K	-1	101
22	V	E	-2	121
23	V	V	4	0
24	I	K	-3	102
25	N	S	1	46
26	D	E	2	45
27	A	A	4	0
28	S	S	4	0
29	F	F	6	0
30	V	I	3	29
31	R	K	2	26
32	D	D	6	0
33	L	L	4	0
34	G	G	6	0
35	A	A	4	0
36	D	D	6	0
37	S	S	4	0
38	L	L	4	0
39	D	D	6	0
40	S	T	1	58
41	V	V	4	0
42	E	E	5	0
43	L	L	4	0
44	V	I	3	29
45	M	M	5	0

46	A	S	1	99
47	L	I	2	5
48	E	E	5	0
49	K	E	1	56
50	E	D	2	45
51	F	F	6	0
52	S	N	1	46
53	I	V	3	29
54	Q	E	2	29
55	I	I	4	0
56	P	P	7	0
57	D	D	6	0
58	E	E	5	0
59	Q	D	0	61
60	A	A	4	0
61	E	E	5	0
62	K	H	-1	32
63	I	I	4	0
64	I	T	-1	89
65	Q	T	-1	42
66	V	V	4	0
67	S	A	1	99
68	A	S	1	99
69	A	V	0	64
70	I	L	2	5
71	D	N	1	23
72	Y	Y	7	0
73	A	L	-1	96
74	E	N	0	42
75	K	E	1	56
76	A	H	-2	86
77	A	S	1	99
78	K	N	0	94

Table S20 Analysis of amino acid variants found exclusively in unsuccessful ALGO sequences.

Position	Variant	Count	Rarity (vs input MSA)	Equivalent (*Ec*AcpP)	BLOSUM62 (vs *Ec*AcpP)	Grantham Distance (vs *Ec*AcpP)
2	M	1	0.1066	S	-1	135
3	E	2	0.0711	T	1	58
	S	1	0.1703		-1	65
	D	1	0.2303		-1	85
4	T	1	0.0531	I	-1	89
	V	1	0.2109		3	29
5	P	1	0.0005	E	-3	140
	N	1	0.0028		-3	134
	I	1	0.0078		0	80
	S	1	0.0134		0	42
	F	1	0.0964		-1	93
6	R	1	0.0005	E	1	56
	K	1	0.0166		2	45
	D	2	0.1657		0	54
7	K	4	0.1357	R	2	26
9	E	1	0.0009	K	1	56
	Q	2	0.0249		1	53
10	R	1	0.0078	K	-1	106
	A	1	0.1269		2	26
11	V	1	0.0868	I	3	29
12	L	1	0.0005	I	3	29
	V	4	0.5976		2	5
13	L	1	0.0037	G	0	60
	A	1	0.2903		-4	138
14	N	1	0.0028	E	0	42
	D	1	0.1375		2	45
15	N	1	0.0014	Q	0	46
19	V	1	0.0005	K	1	53
	G	1	0.0032		-2	97
	Q	1	0.0037		-2	127
	E	1	0.1592		1	56
20	G	1	0.0037	Q	-2	87
	V	1	0.0212		-2	96
21	P	1	0.0014	E	-1	65
	V	1	0.0023		0	42
	T	2	0.0171		-1	93
	N	1	0.0268		-2	121
22	K	2	0.1384	E	1	56
24	H	1	0.0018	T	-1	42
	Q	1	0.0171		0	69
	V	1	0.066		-2	47
25	T	1	0.042	N	0	65
	L	1	0.0452		0	42
	E	1	0.1343		-3	153
26	H	1	0.0046	N	1	46

	S	2	0.1029		1	68
27	S	2	0.3096	A	1	99
28	I	1	0.0009	S	1	99
	A	3	0.012		-2	142
30	A	1	0.006	V	0	64
31	Q	2	0.0014	E	0	42
	N	1	0.1444		2	45
	D	1	0.1444		2	29
40	F	1	0.0005	T	-2	103
	Q	1	0.0028		-1	42
44	L	1	0.0023	V	1	32
46	T	1	0.0245	A	0	58
47	M	1	0.0111	L	0	22
	F	1	0.2921		2	15
49	N	1	0.0009	E	0	42
50	S	1	0.0005	E	-1	107
	Q	2	0.0097		0	80
	A	1	0.0208		2	29
52	H	1	0.0009	D	-1	81
53	F	1	0.0175	T	-1	149
	C	1	0.1957		-2	103
54	N	1	0.0032	E	-1	107
	D	1	0.0065		0	42
	A	1	0.0443		2	45
58	D	2	0.2635	E	2	45
59	T	1	0.0005	E	-1	65
	H	2	0.0042		0	40
62	D	2	0.0014	K	-1	78
	T	1	0.0554		-1	101
64	S	1	0.0535	T	1	58
65	S	1	0.0475	T	1	58
68	F	1	0.0018	A	-2	113
	Q	2	0.2843		-2	126
	D	1	0.3899		-1	91
70	V	2	0.2796	I	3	29
71	L	1	0.0005	D	0	65
	H	2	0.0009		-1	81
	S	1	0.0655		-4	172
73	V	1	0.2635	I	3	29
74	G	1	0.0078	N	0	65
	T	2	0.0951		0	80
75	D	1	0.0406	G	0	60
	N	1	0.084		0	80
	A	2	0.3009		-1	94
76	S	1	0.0129	H	-1	89
	Q	1	0.0152		-1	32
	K	1	0.0452		1	68

	N	1	0.2884		0	24
78	G	1	0.0097	A	0	60
	Q	1	0.0125		-1	91

Sequence Analysis (Fig. S1-3)

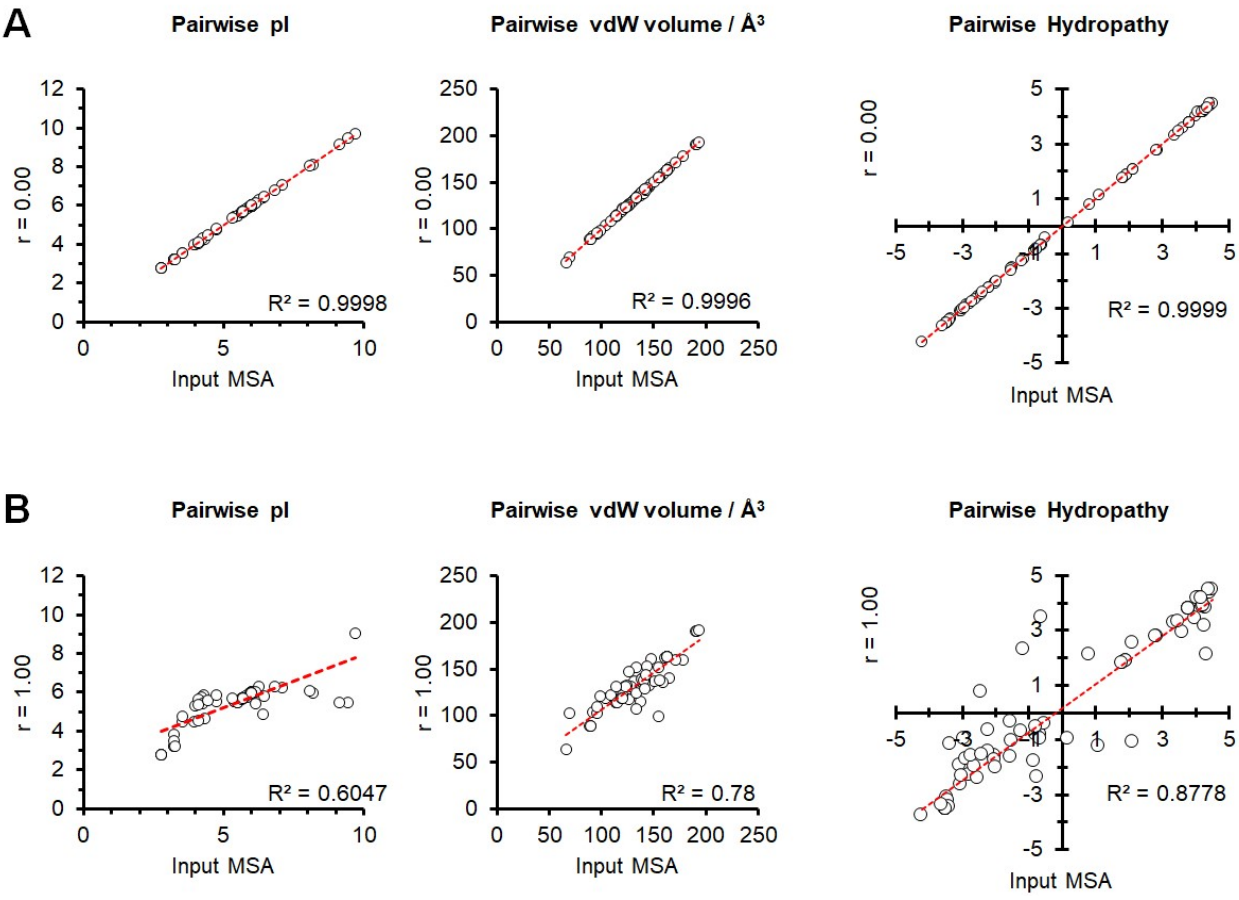

Fig. S1 Example pairwise correlation plots for sequence sets r = 0.00 (A) and r = 1.00 (B).

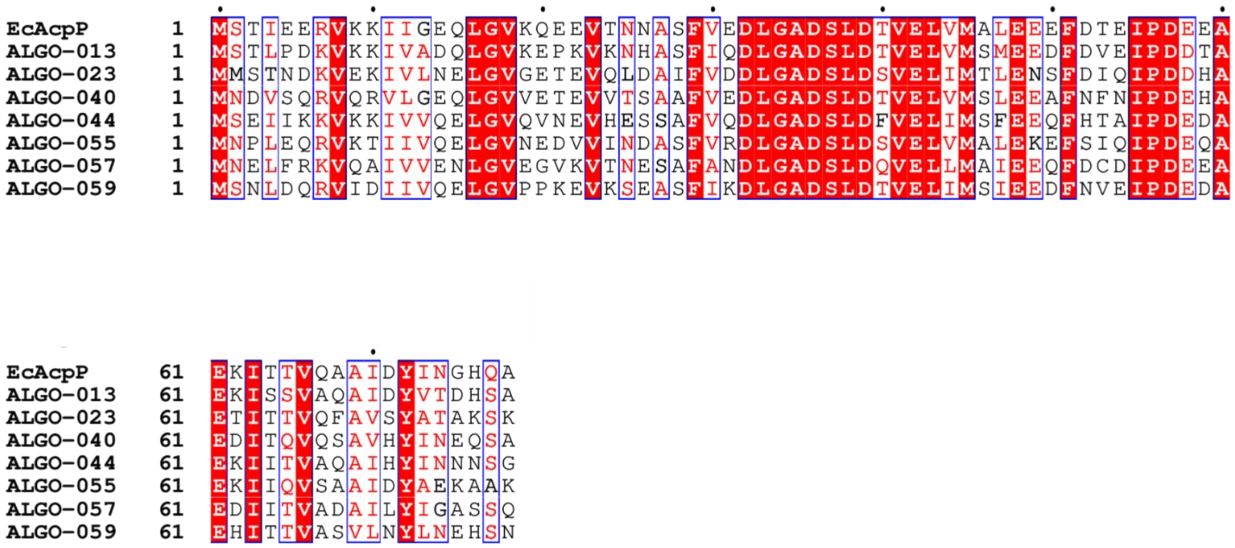

Fig. S2 Multiple sequence alignment of ALGO-CP candidates and *Ec*AcpP.

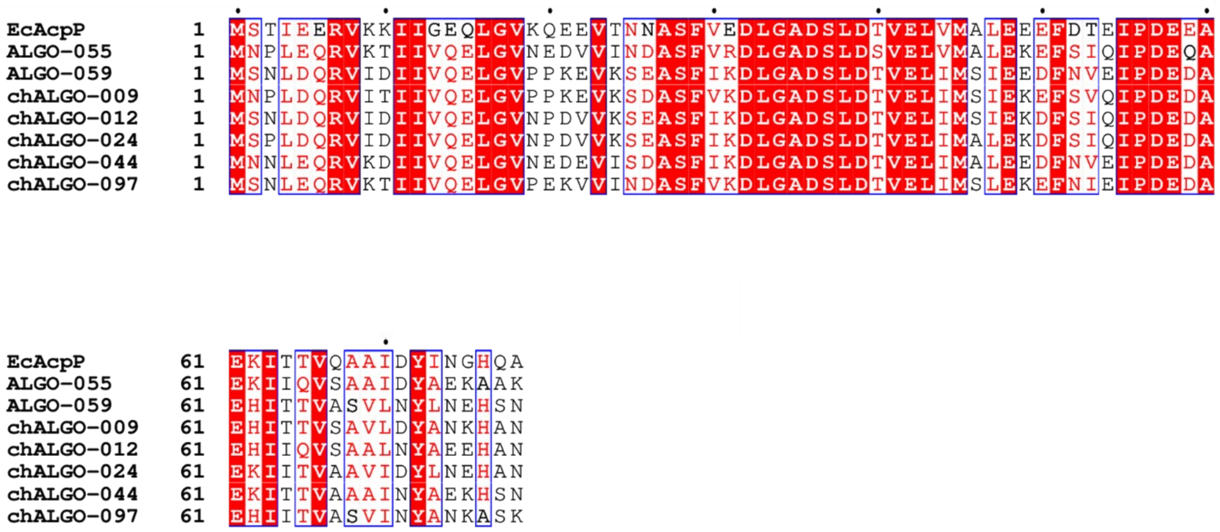

Fig. S3 Multiple sequence alignment of [ch]ALGO-CP chimeras, parental ALGO-055 and ALGO-059, and *Ec*AcpP.

Purification Data (Figs. S4-S14)

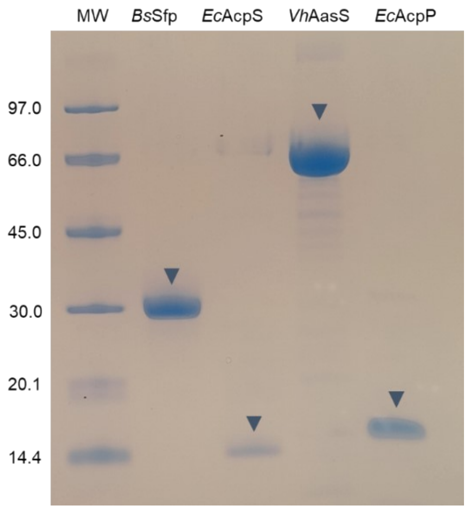

Fig. S4 Annotated SDS-PAGE gel photograph showing purified *Bs*Sfp, *Ec*AcpS, *Vh*AasS and *Ec*AcpP. An Amersham low molecular weight calibration kit was used as the protein ladder (lane MW).

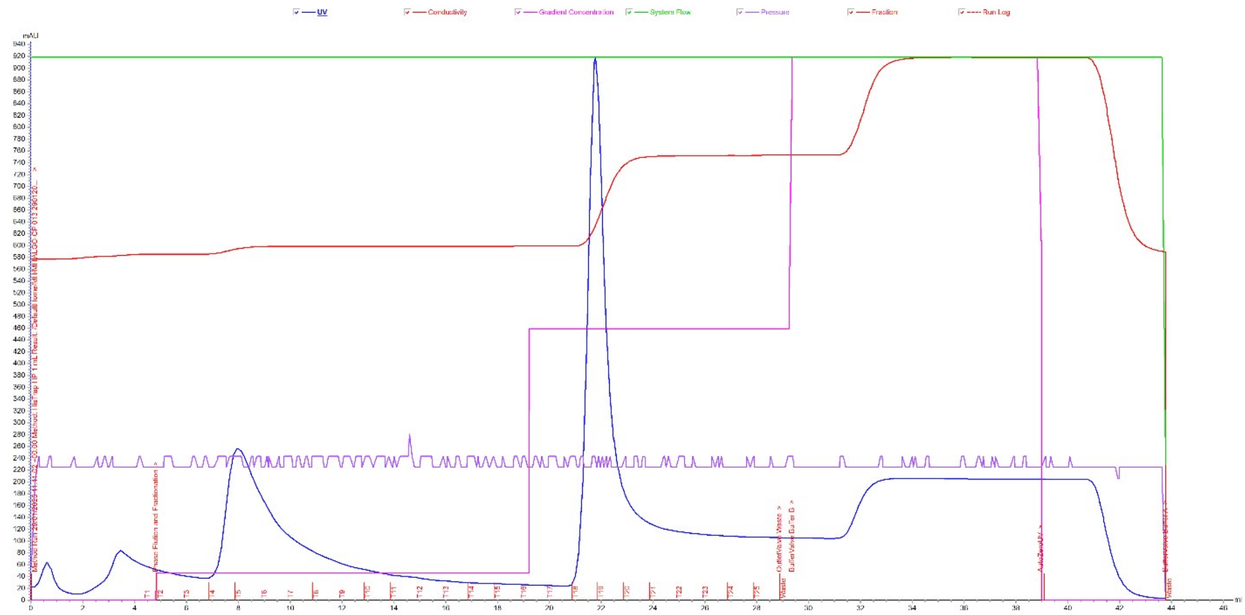

Fig. S5 A_{280} chromatogram of ALGO-013 purification by Ni^{2+}-affinity chromatography.

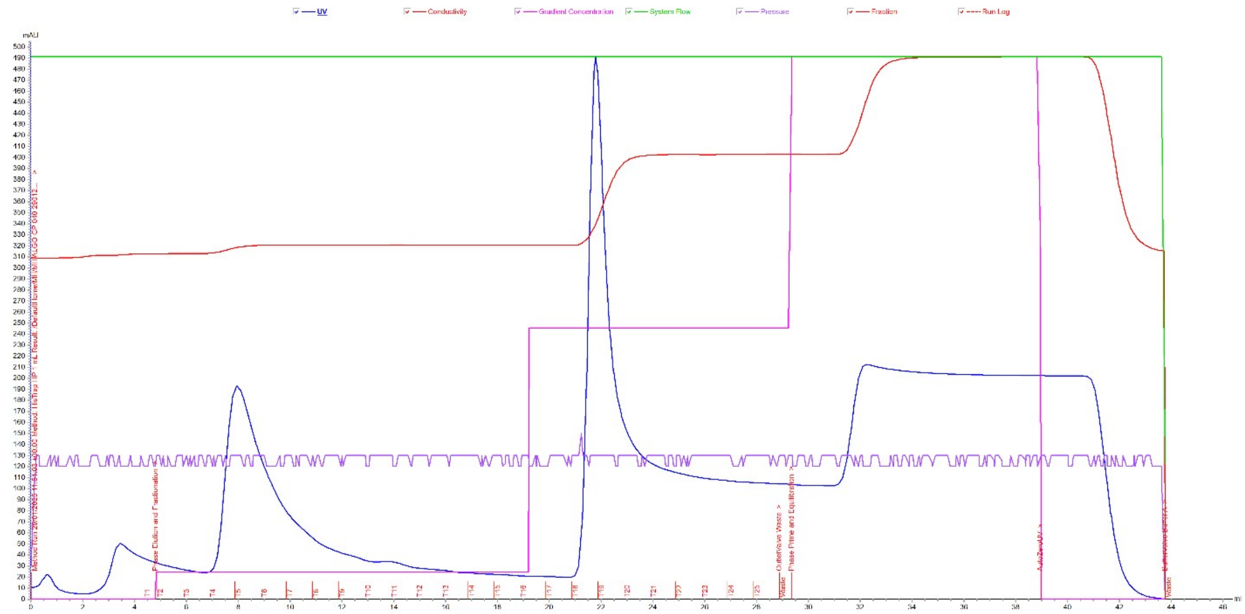

Fig. S6 A_{280} chromatogram of ALGO-040 purification by Ni^{2+}-affinity chromatography.

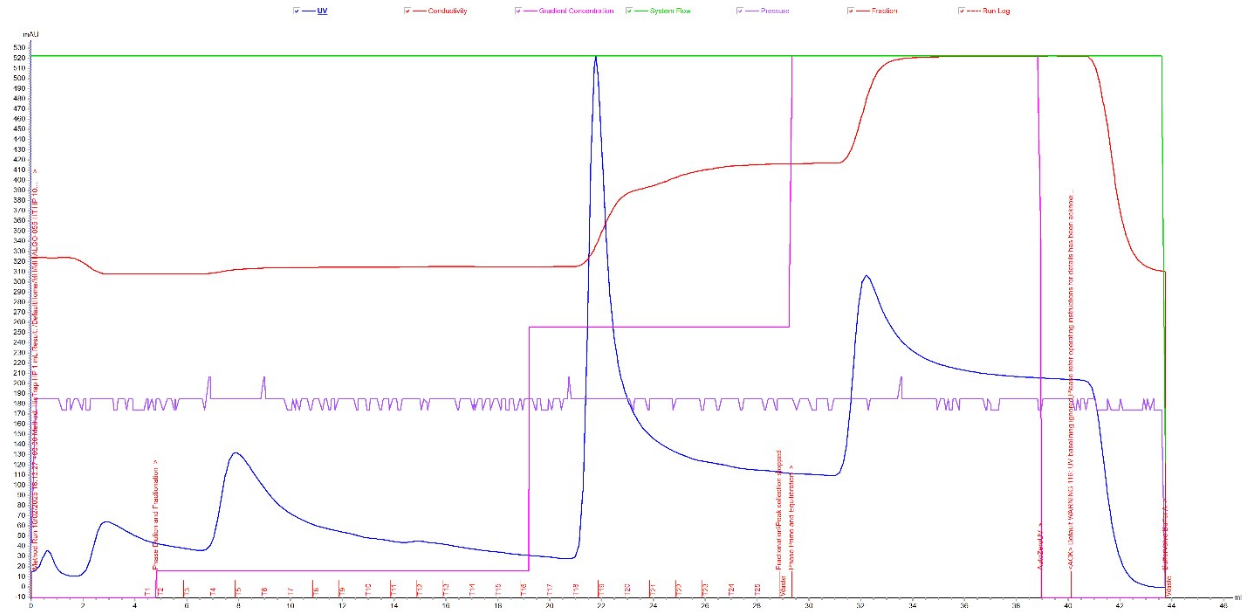

Fig. S7 A_{280} chromatogram of ALGO-055 purification by Ni^{2+}-affinity chromatography.

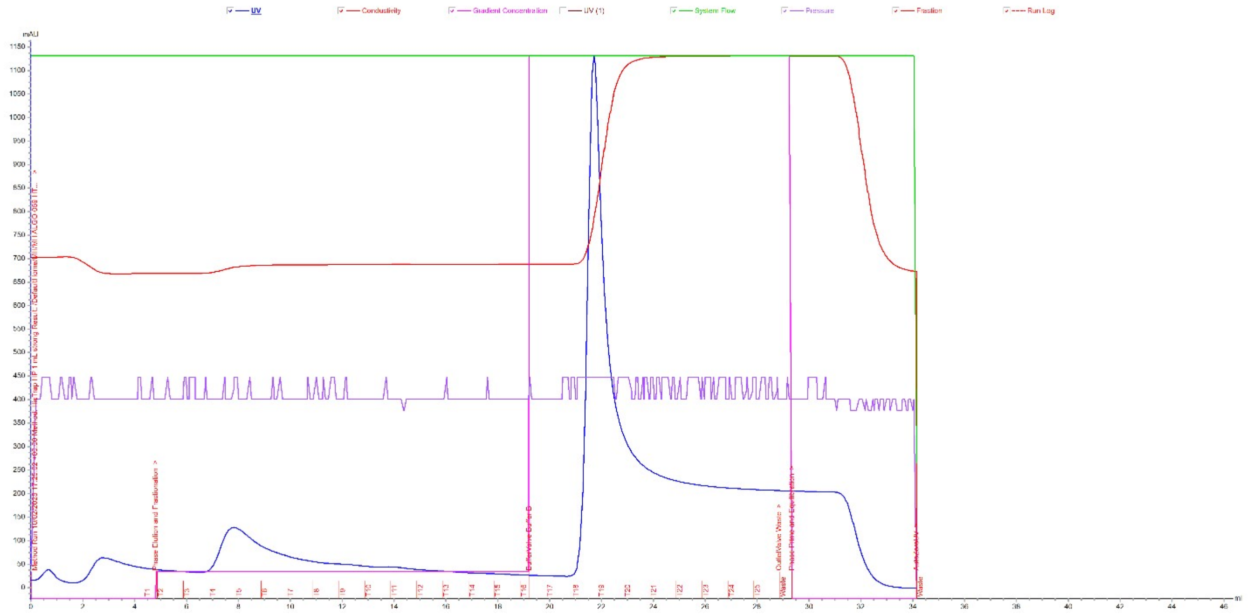

Fig. S8 A_{280} chromatogram of ALGO-059 purification by Ni^{2+}-affinity chromatography.

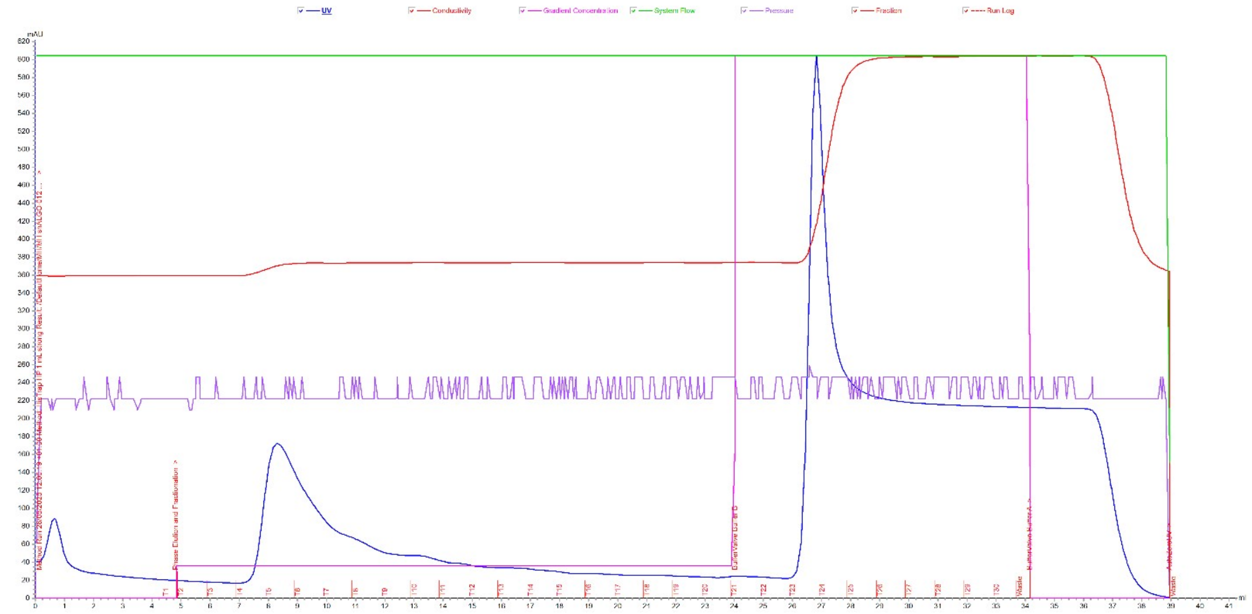

Fig. S9 A_{280} chromatogram of ^{ch}ALGO-012 purification by Ni^{2+}-affinity chromatography.

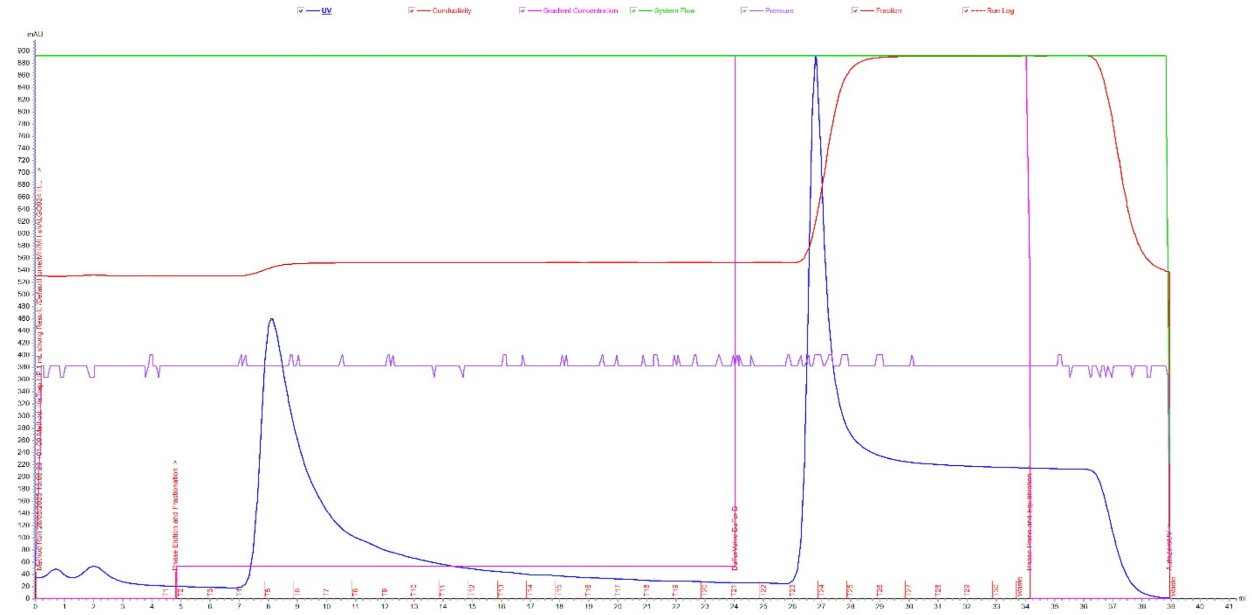

Fig. S10 A_{280} chromatogram of ^{ch}ALGO-024 purification by Ni^{2+}-affinity chromatography.

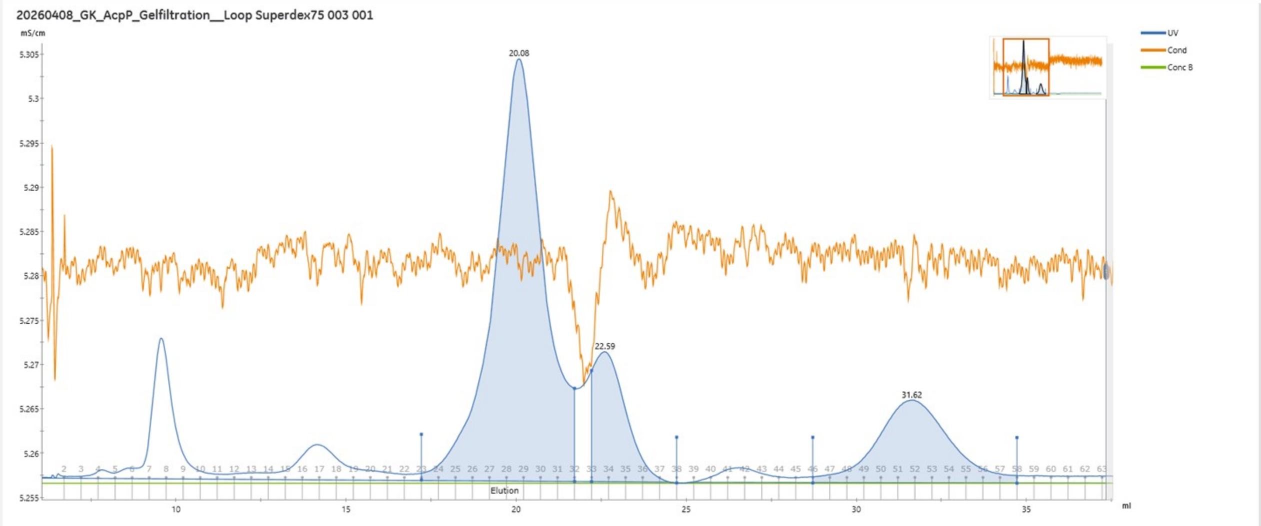

Peak Table - UV

Peak	Retention (ml)	Area (ml*mAU)	Area %	Ext coeff. (mg ml⁻¹ cm⁻¹)	Fraction(s)	Volume (ml)	Conductivity (mS/cm)
Peak A	20.084	32.64	66.98		23 - 31	4.502	5.28
Peak B	22.591	6.467	13.27		33 - 37	2.501	5.28
Peak C	31.624	9.623	19.75		46 - 57	6.002	5.28

Fig. S11 A_{280} chromatogram of purified C_{12}-*Ec*AcpP using Superdex S75 SEC (20.08 mL).

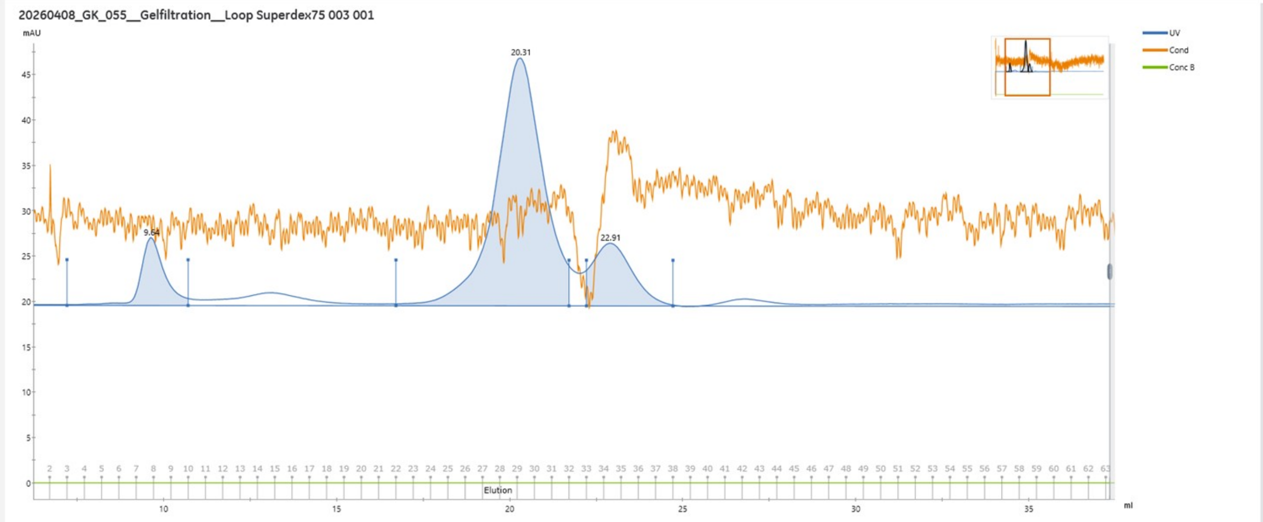

Peak Table - UV

Peak	Retention (ml)	Area (ml*mAU)	Area %	Ext coeff. (mg ml⁻¹ cm⁻¹)	Fraction(s)	Volume (ml)	Conductivity (mS/cm)
Peak A	9.635	5.942	10.2		3 - 9	3.500	5.28
Peak B	20.310	43.23	74.21		22 - 31	5.000	5.28
Peak C	22.907	9.086	15.6		33 - 37	2.499	5.28

Fig. S12 A_{280} chromatogram of purified C_{12}-ALGO-055 using Superdex S75 SEC (20.31 mL).

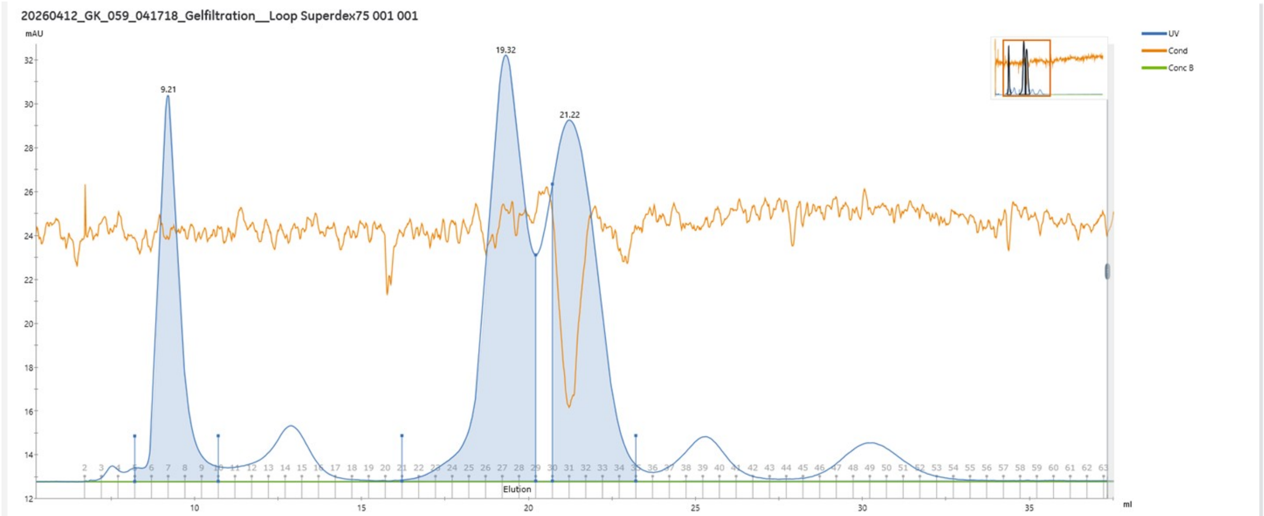

▴ Peak Table - UV

Peak	Retention (ml)	Area (ml*mAU)	Area %	Ext coeff. (mg ml⁻¹ cm⁻¹)	Fraction(s)	Volume (ml)	Conductivity (mS/cm)
Peak A	9.207	12.85	20.46		5 - 9	2.501	5.28
Peak B	19.319	26.13	41.58		21 - 28	4.004	5.28
Peak C	21.221	23.85	37.96		30 - 34	2.499	5.27

Fig. S13 A_{280} chromatogram of purified C_{12}-ALGO-059 using Superdex S75 SEC (19.32 mL).

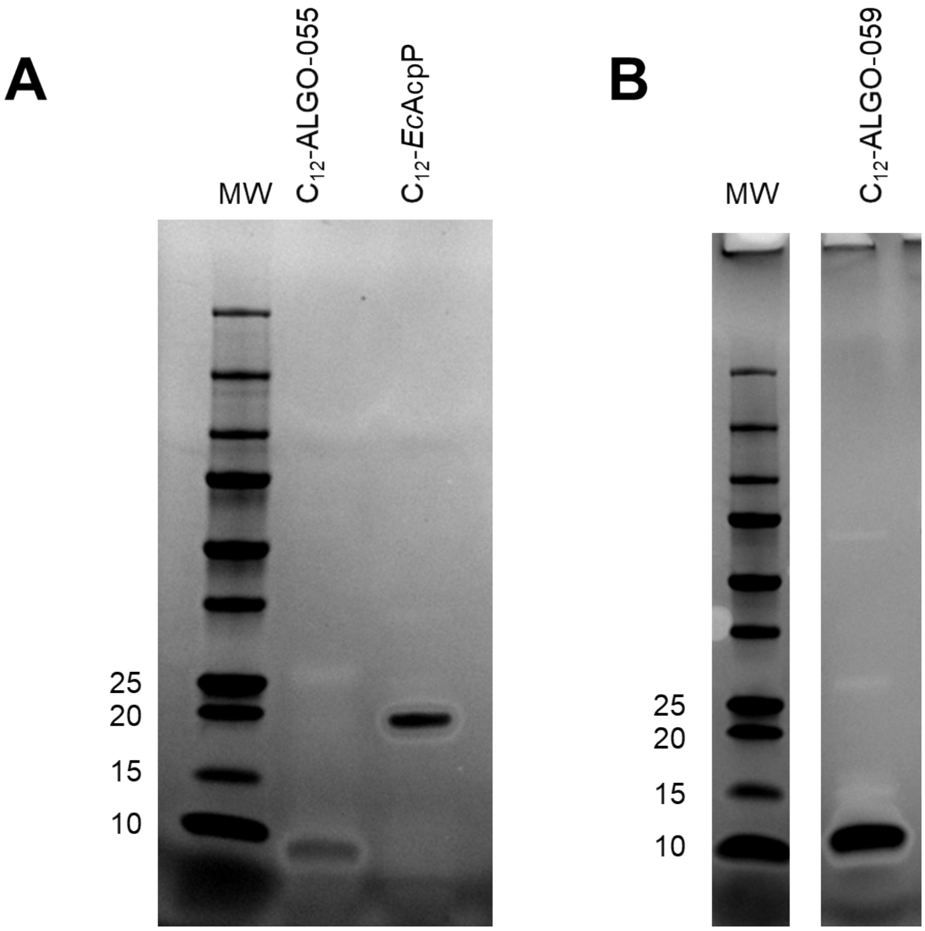

Fig. S14 SDS-PAGE of purified acylated proteins (A) C_{12}-*Ec*AcpP and C_{12}-ALGO-055 (B) C_{12}-ALGO-059. Gels were imaged using a FluorChem M FM1059.

168

LC/ESI-MS data (Figs. S15-S37)

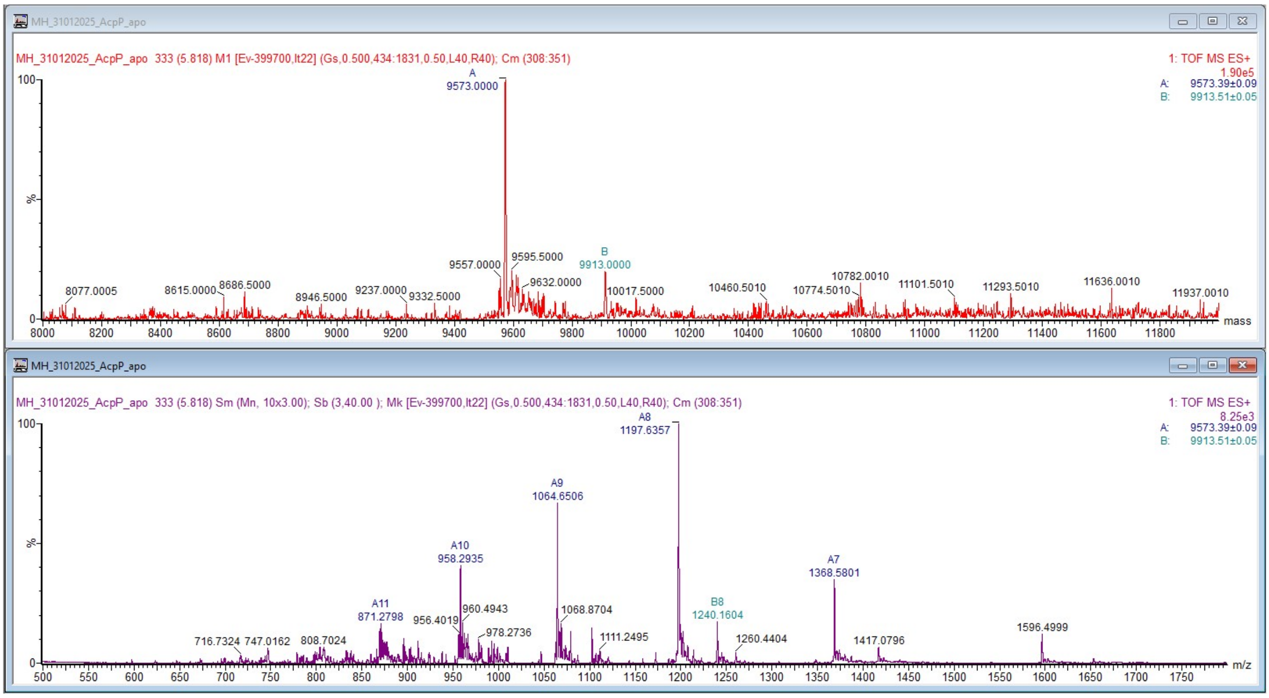

Fig. S15 *apo-Ec*AcpP charge envelope (purple) and deconvoluted mass (red).

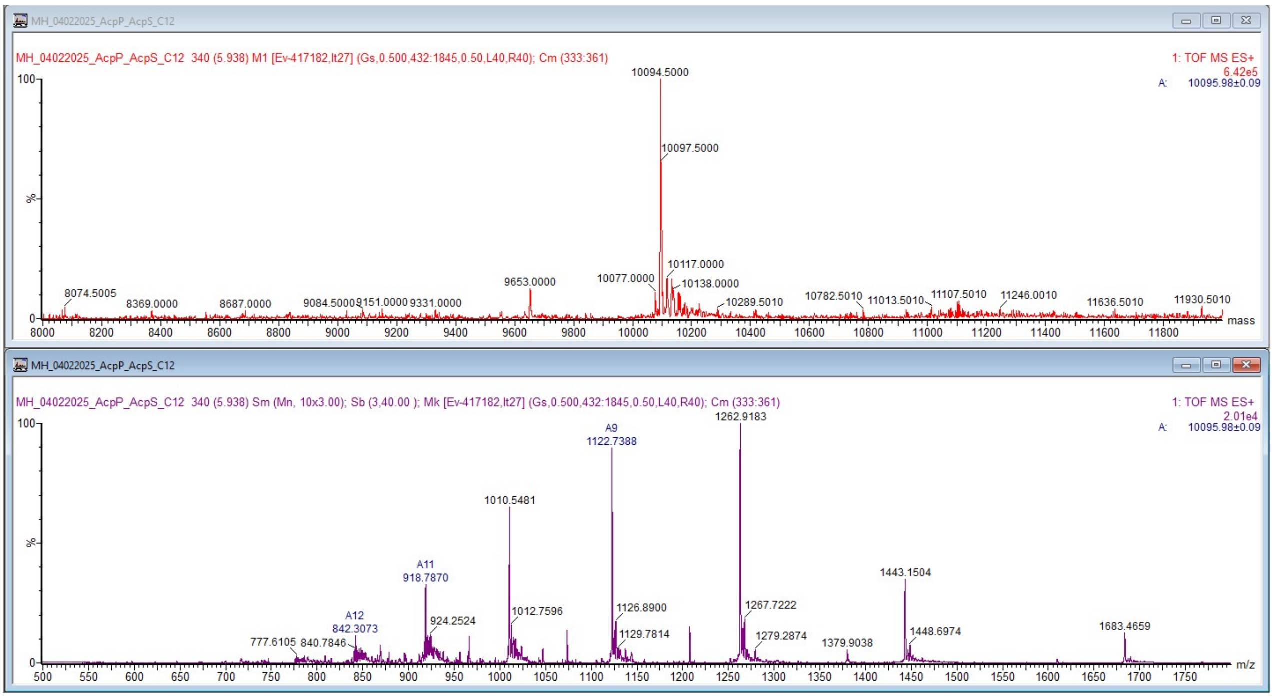

Fig. S16 *holo-Ec*AcpP charge envelope (purple) and deconvoluted mass (red).

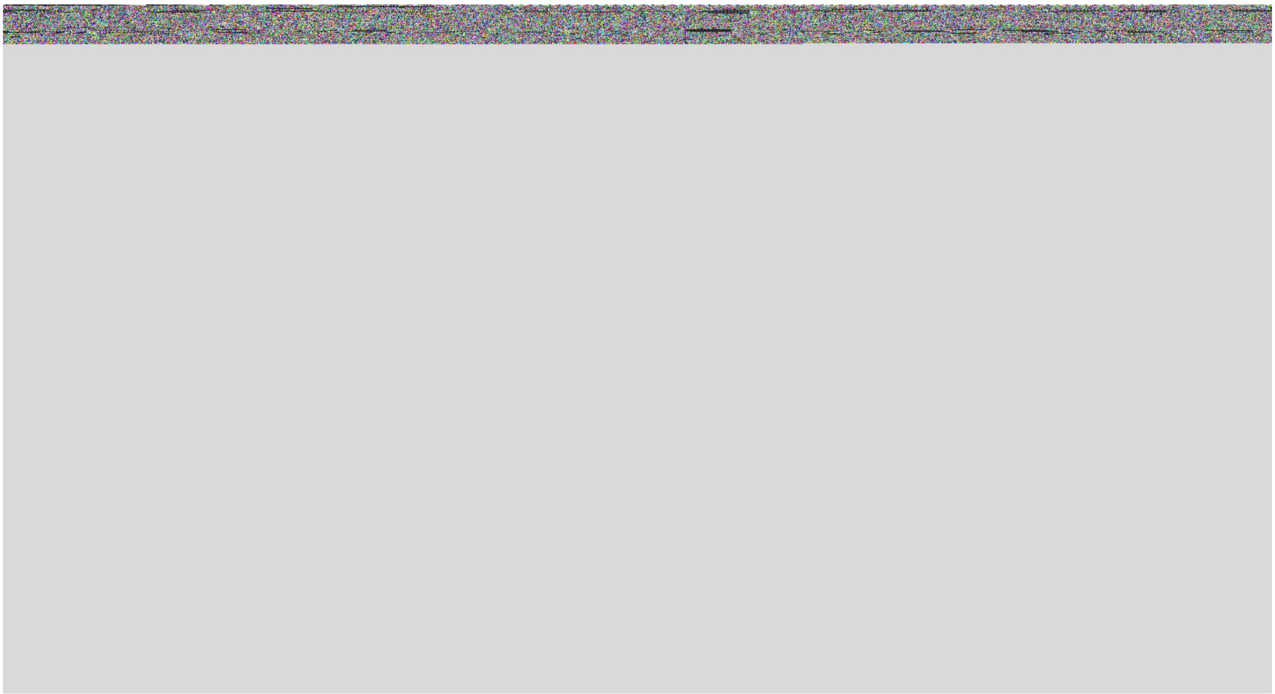

Fig. S17 C_{12}-*Ec*AcpP charge envelope (purple) and deconvoluted mass (red).

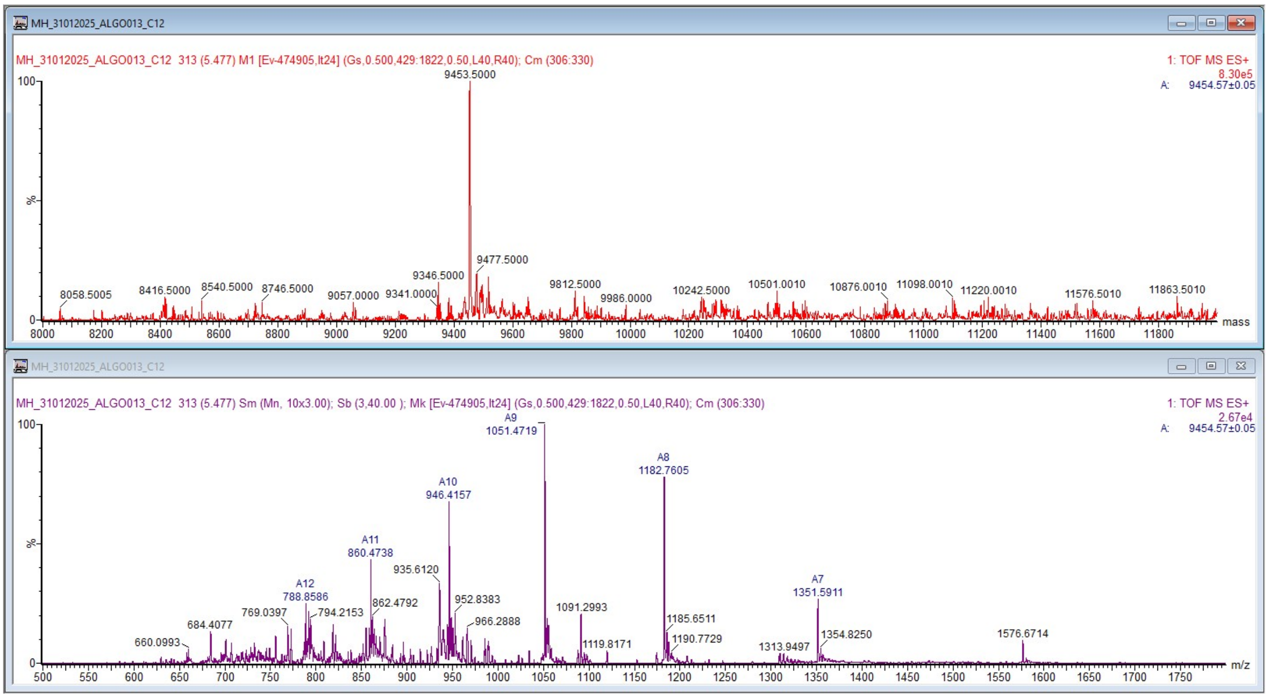

Fig. S18 *apo*-ALGO-013 charge envelope (purple) and deconvoluted mass (red).

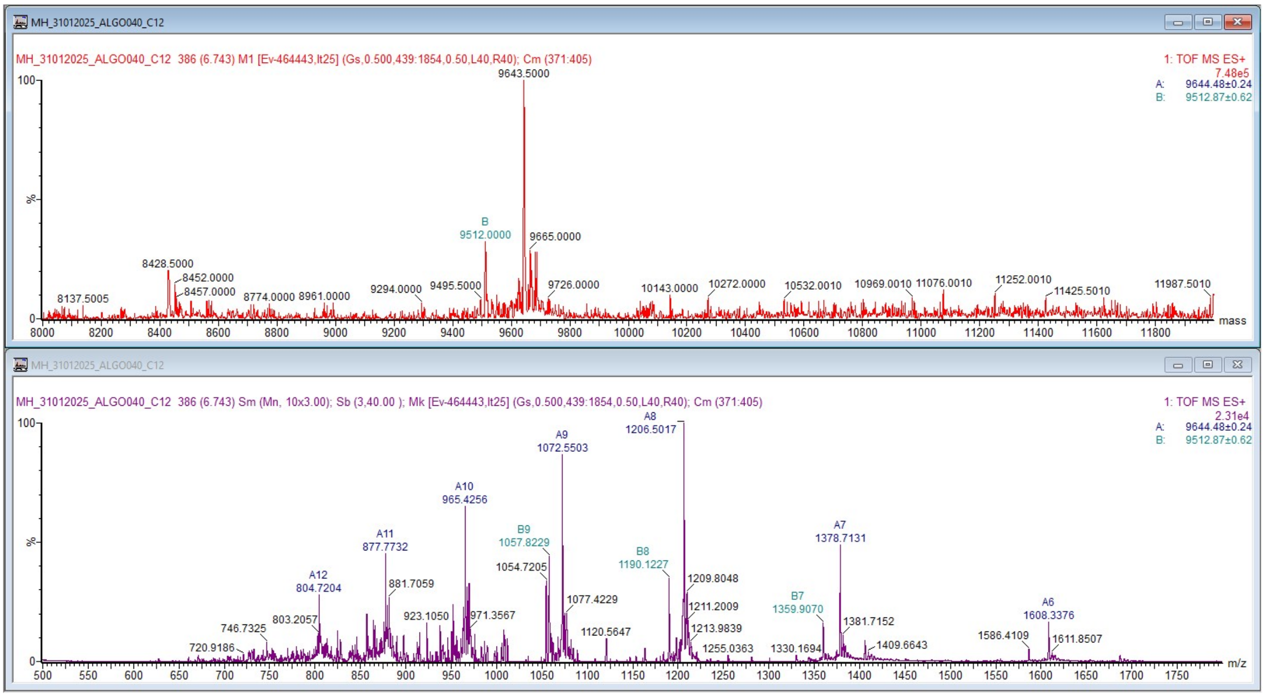

Fig. S19 *apo*-ALGO-040 charge envelope (purple) and deconvoluted mass (red).

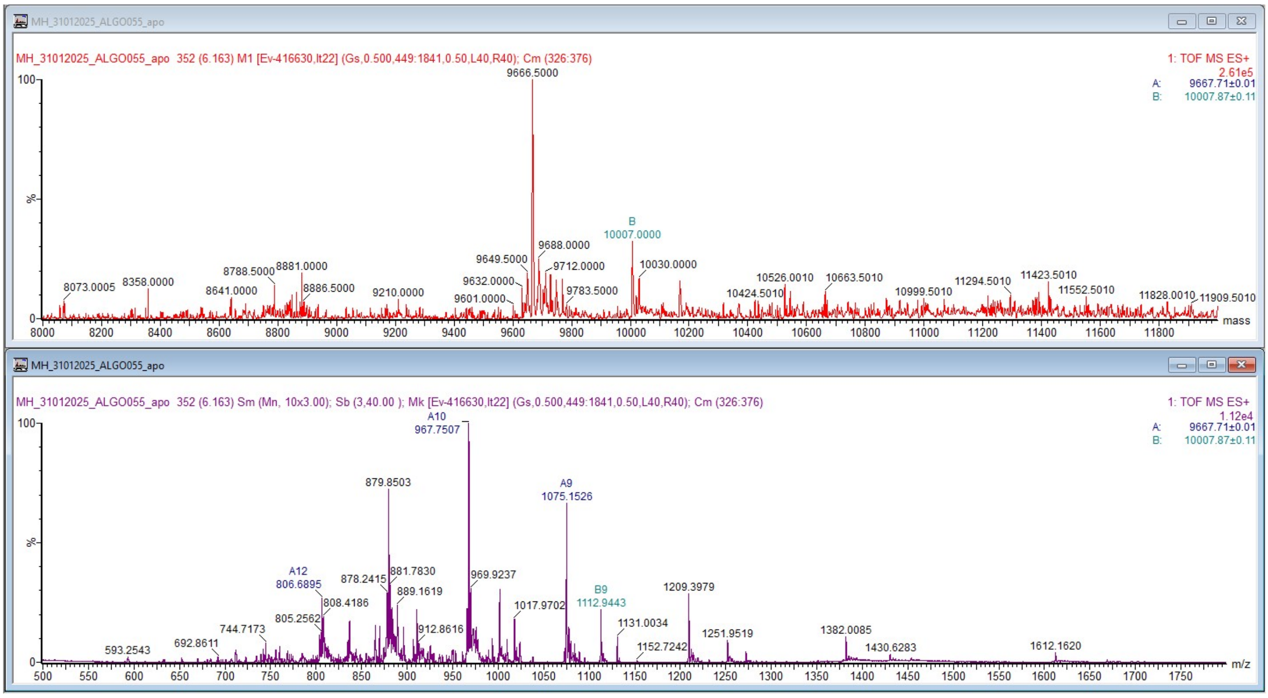

Fig. S20 *apo*-ALGO-055 charge envelope (purple) and deconvoluted mass (red).

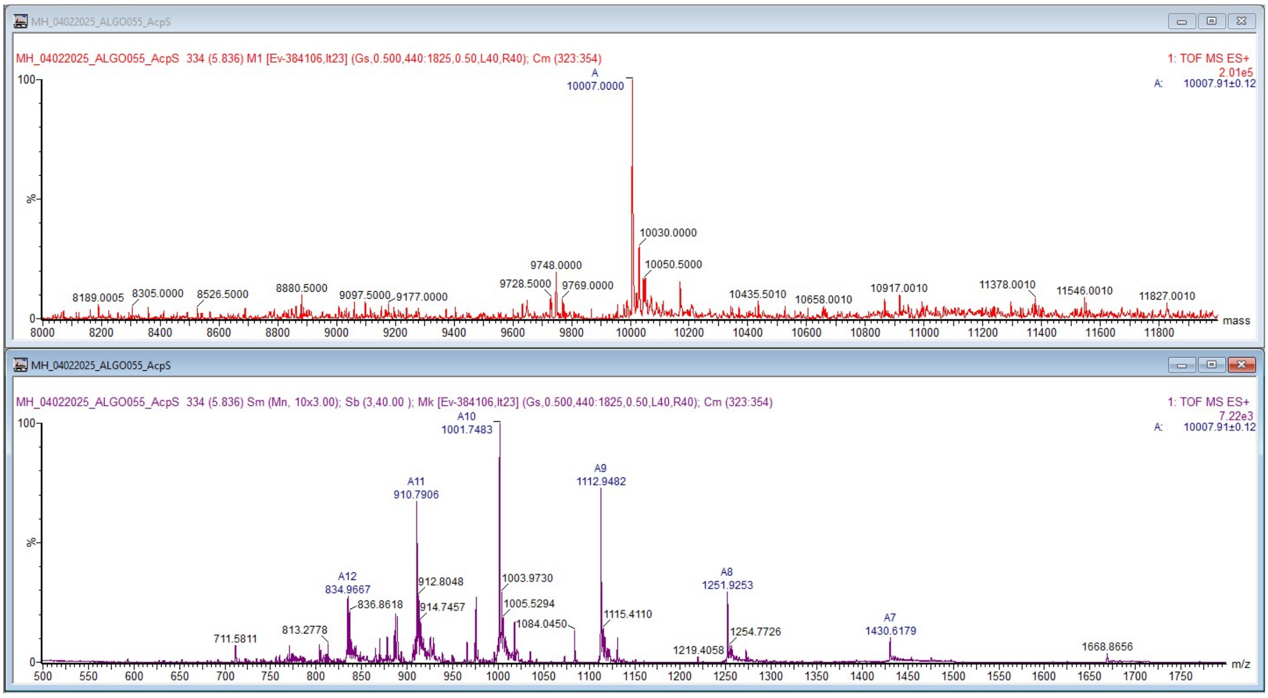

Fig. S21 *holo*-ALGO-055 charge envelope (purple) and deconvoluted mass (red).

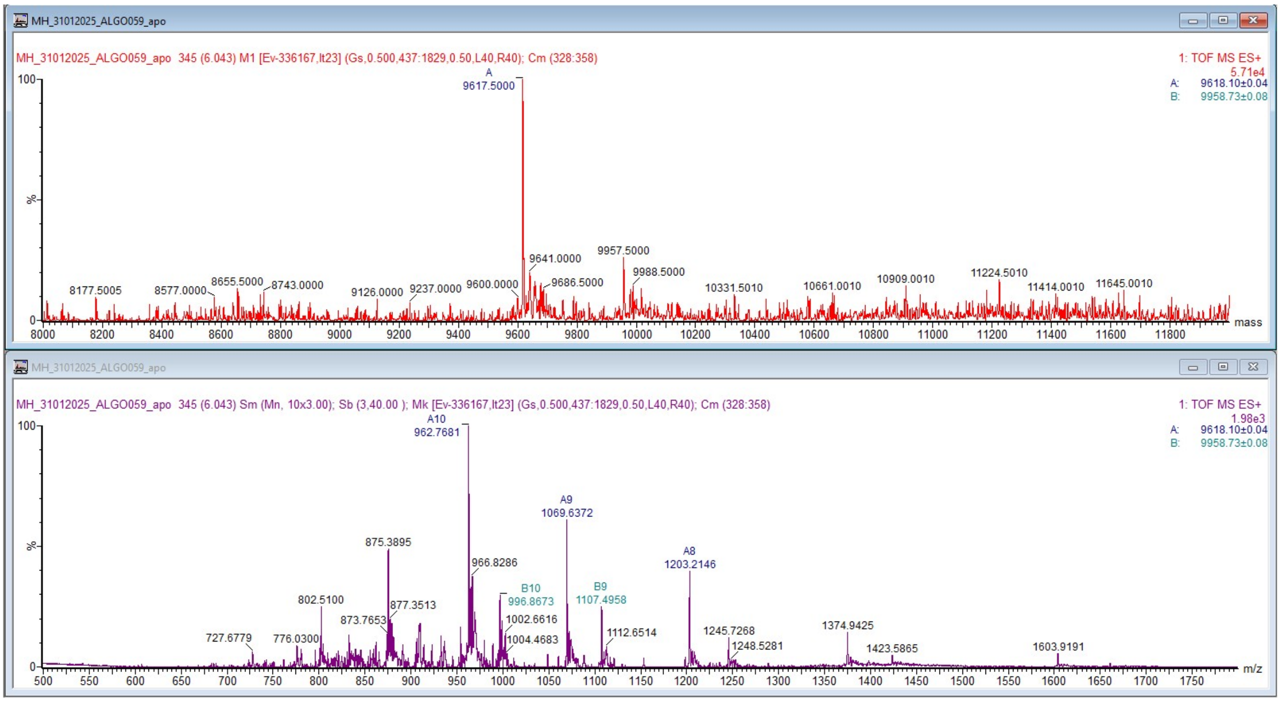

Fig. S22 *apo*-ALGO-059 charge envelope (purple) and deconvoluted mass (red).

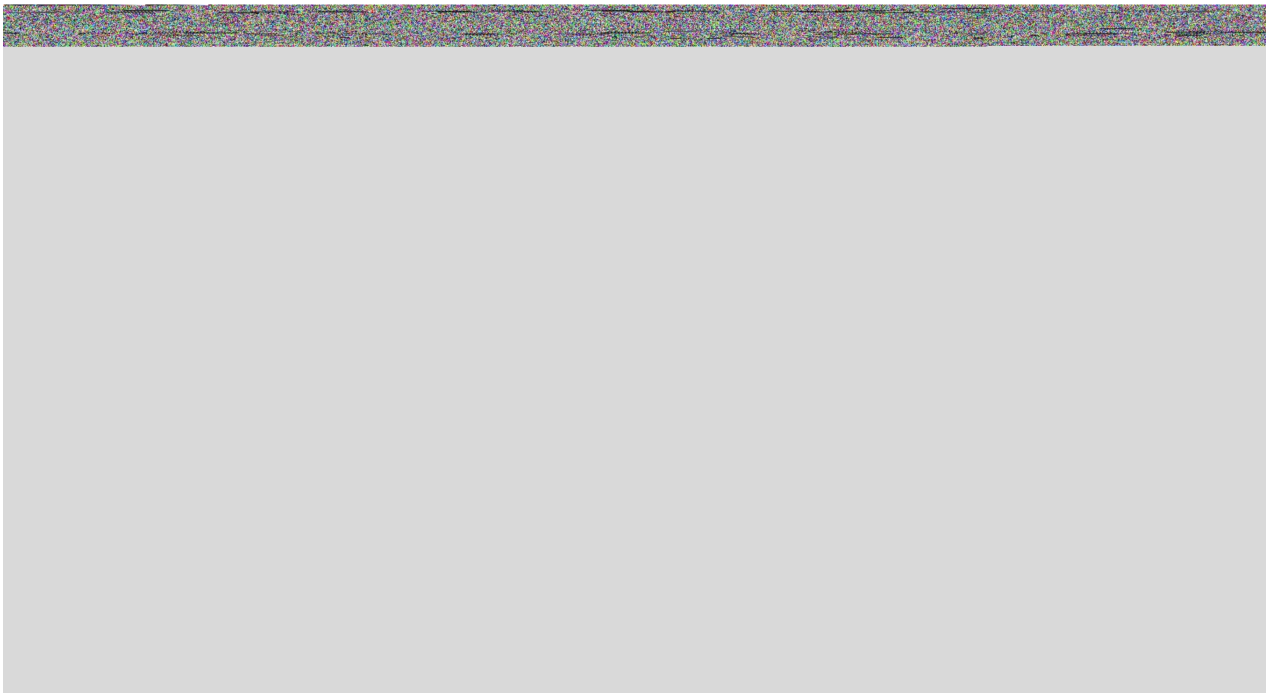

Fig. S23 *holo*-ALGO-059 charge envelope (purple) and deconvoluted mass (red).

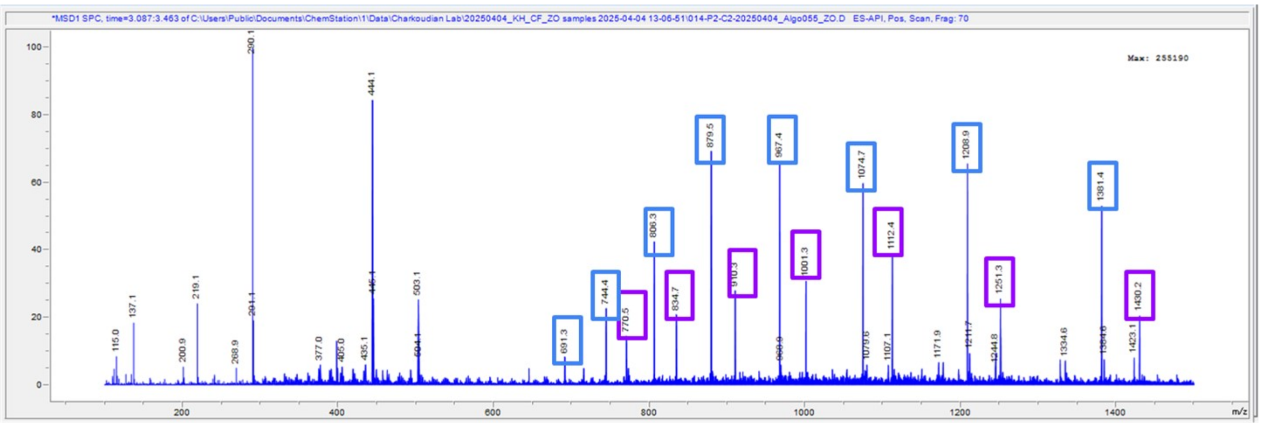

Fig. S24 Attempted apo→holo conversion of immobilised ALGO-055 using *Ec*AcpS. Recorded using an Agilent Technologies InfinityLab G6125B single quadrupole MS.

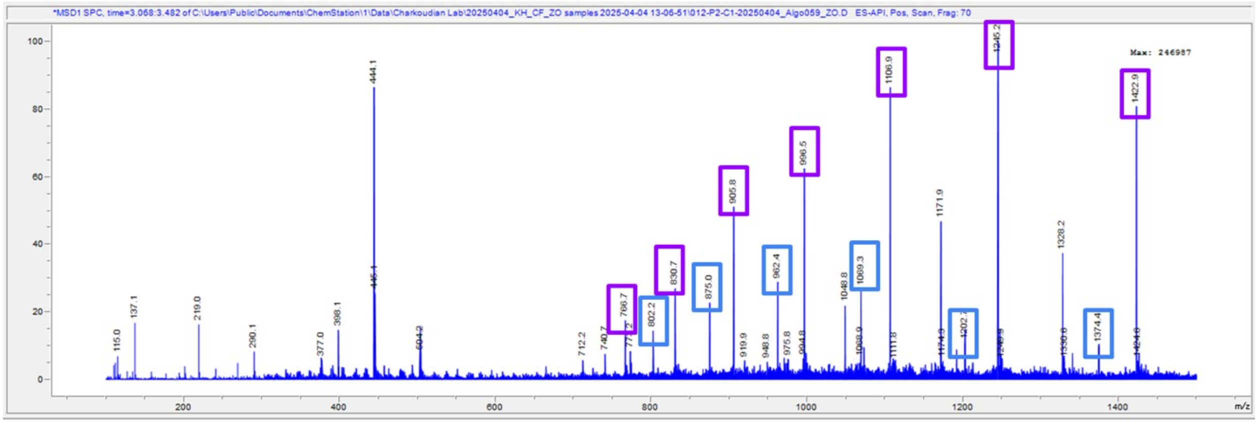

apo-ALGO-059

holo-ALGO-059

Fig. S25 Attempted apo→holo conversion of immobilised ALGO-059 using *Ec*AcpS. Recorded using an Agilent Technologies InfinityLab G6125B single quadrupole MS.

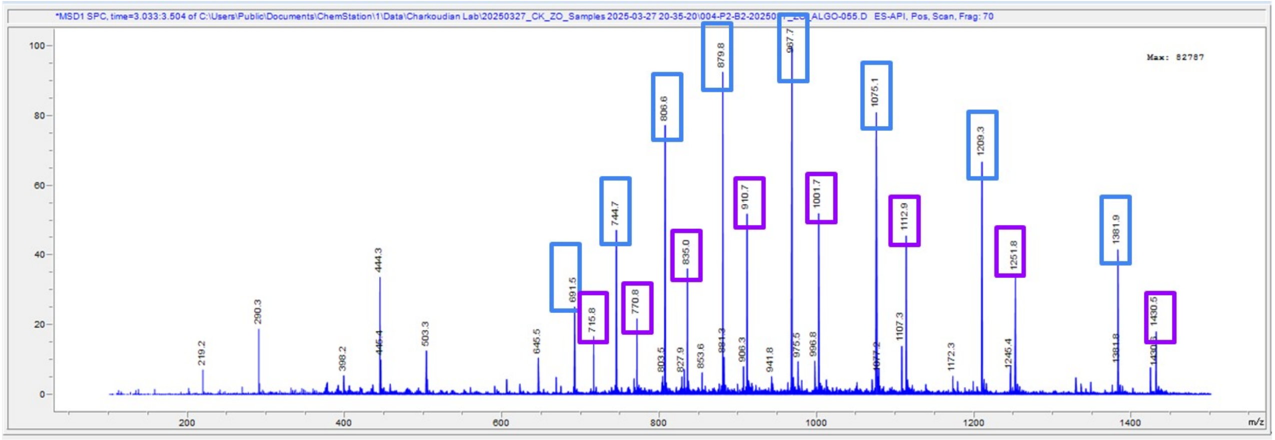

apo-ALGO-055

holo-ALGO-055

Fig. S26 Attempted apo→holo conversion of ALGO-055 using *Bs*Sfp. Recorded using an Agilent Technologies InfinityLab G6125B single quadrupole MS.

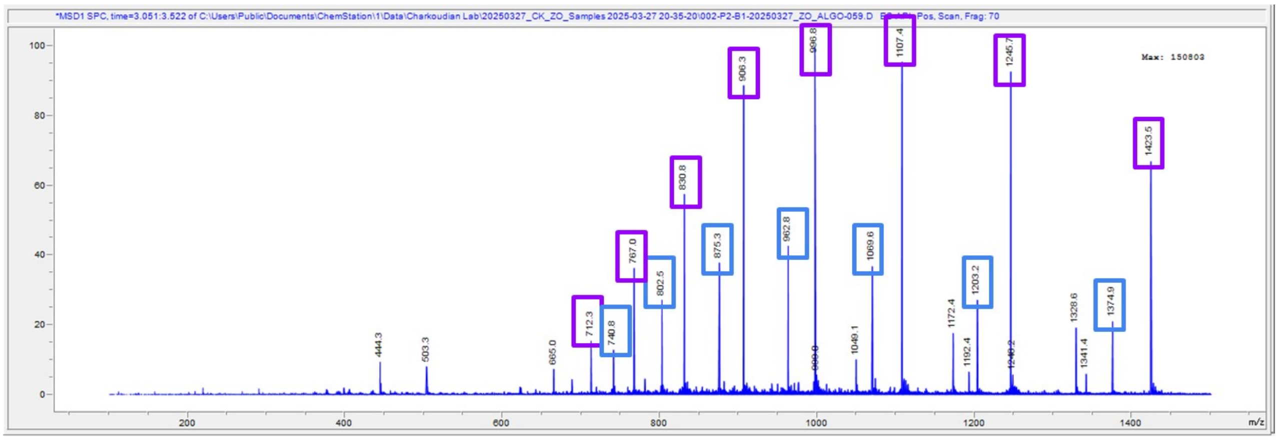

apo-ALGO-059

holo-ALGO-059

Fig. S27 Attempted apo→holo conversion of ALGO-059 using *Bs*Sfp. Recorded using an Agilent Technologies InfinityLab G6125B single quadrupole MS.

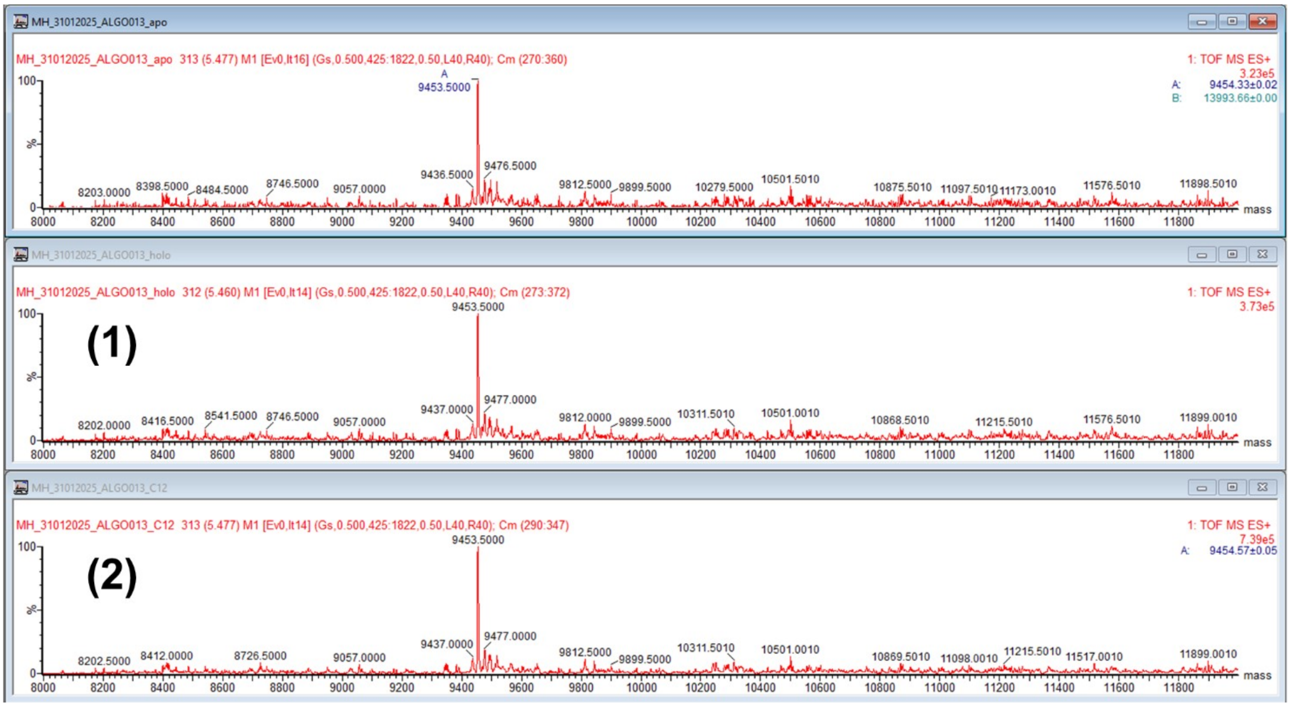

Fig. S28 Unsuccessful apo→holo→acyl conversion of ALGO-013 using *Ec*AcpS (1) and VhAasS (2).

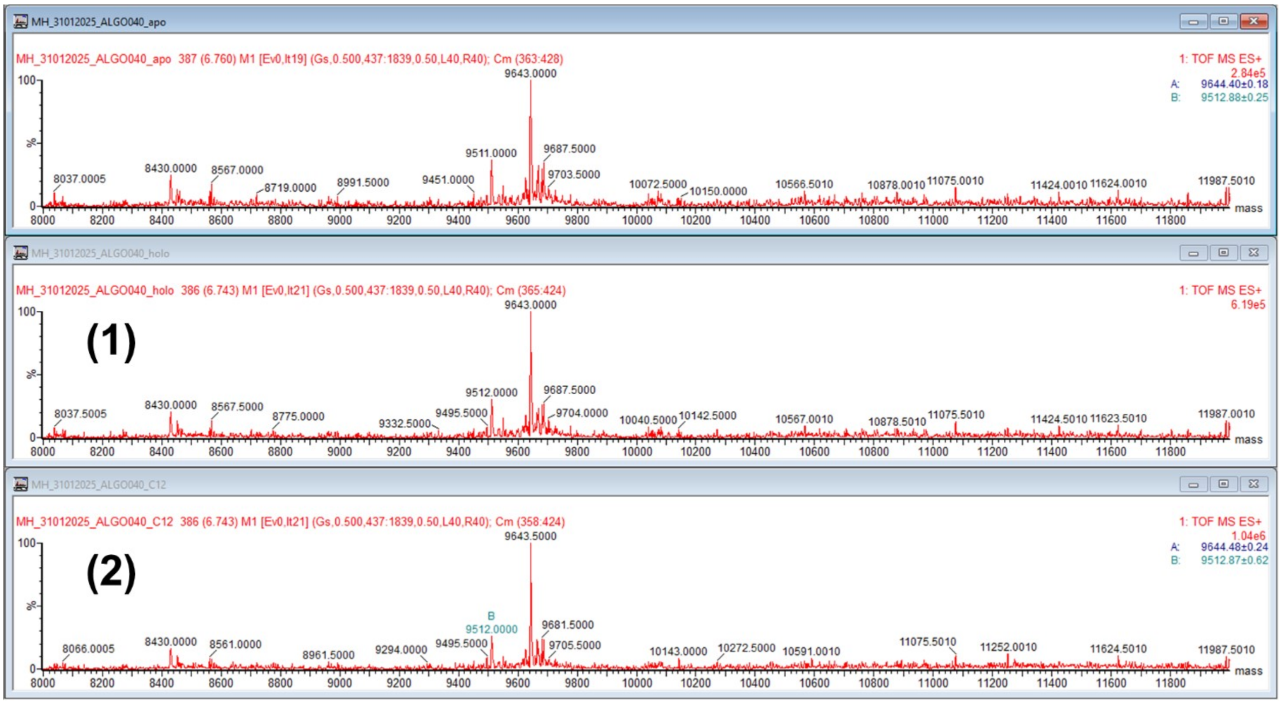

Fig. S29 Unsuccessful apo→holo→acyl conversion of ALGO-040 using *Ec*AcpS (1) and VhAasS (2).

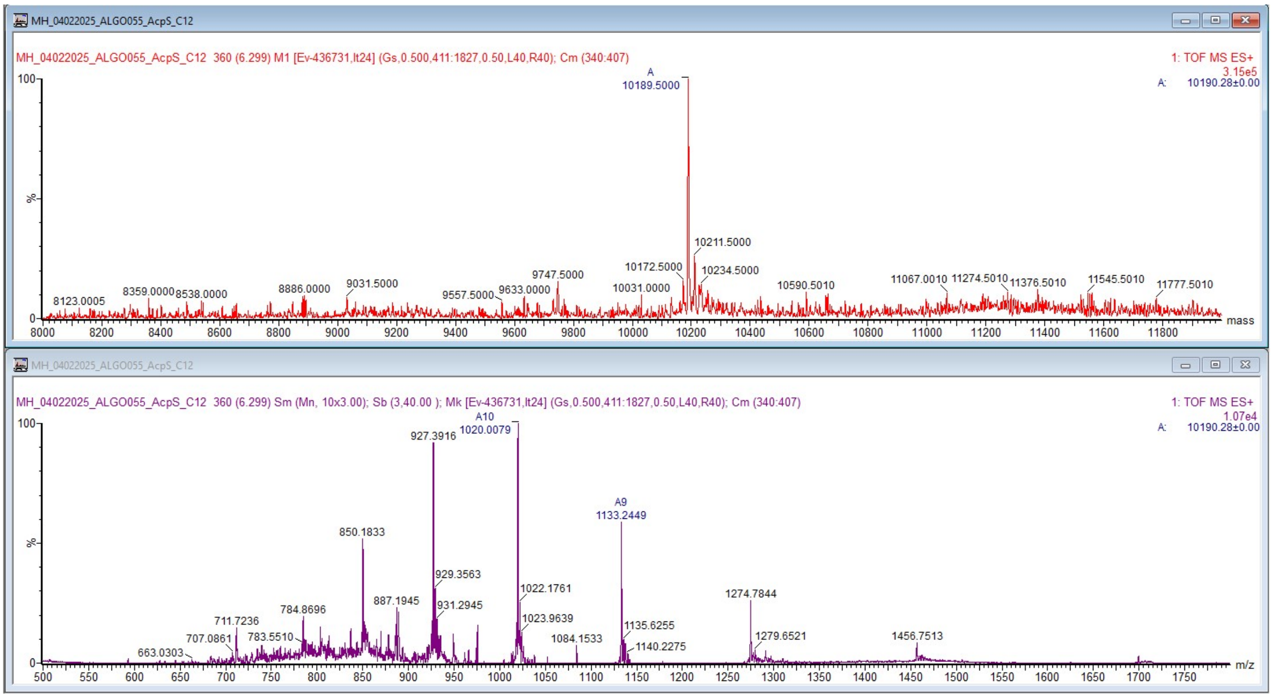

Fig. S30 C_{12}-ALGO-055 charge envelope.

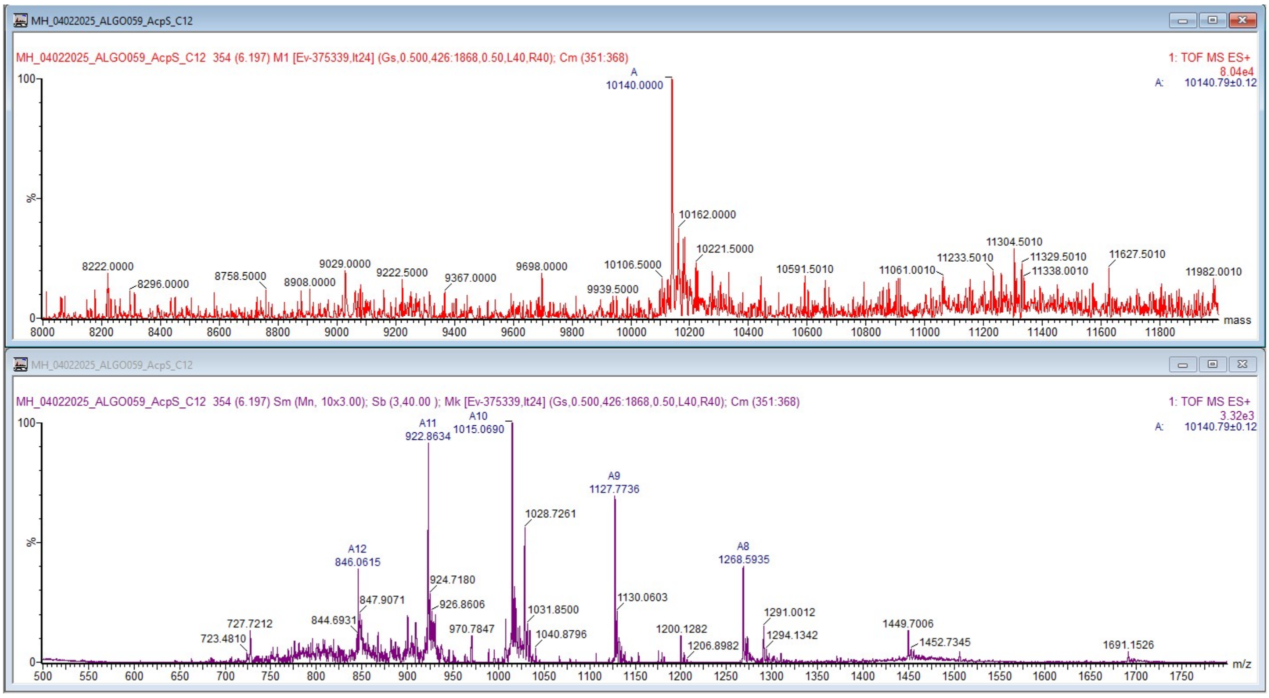

Fig. S31 C_{12}-ALGO-059 charge envelope.

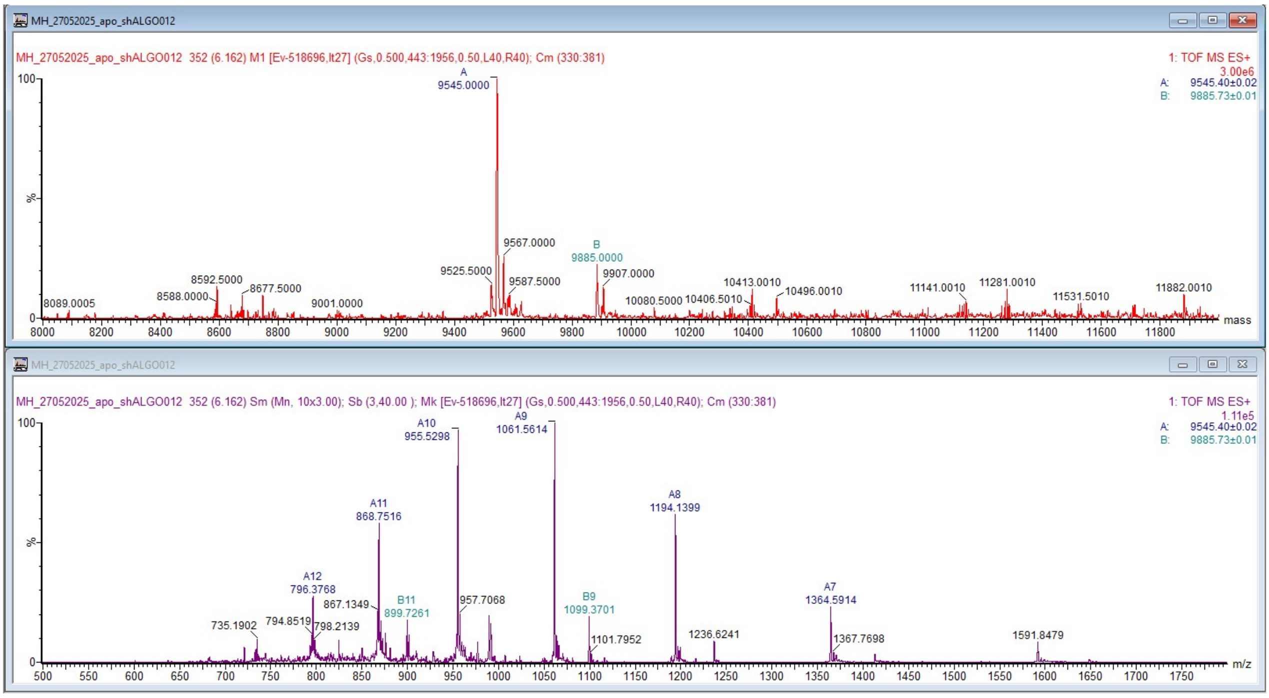

Fig. S32 *apo*-^{ch}ALGO-012 charge envelope.

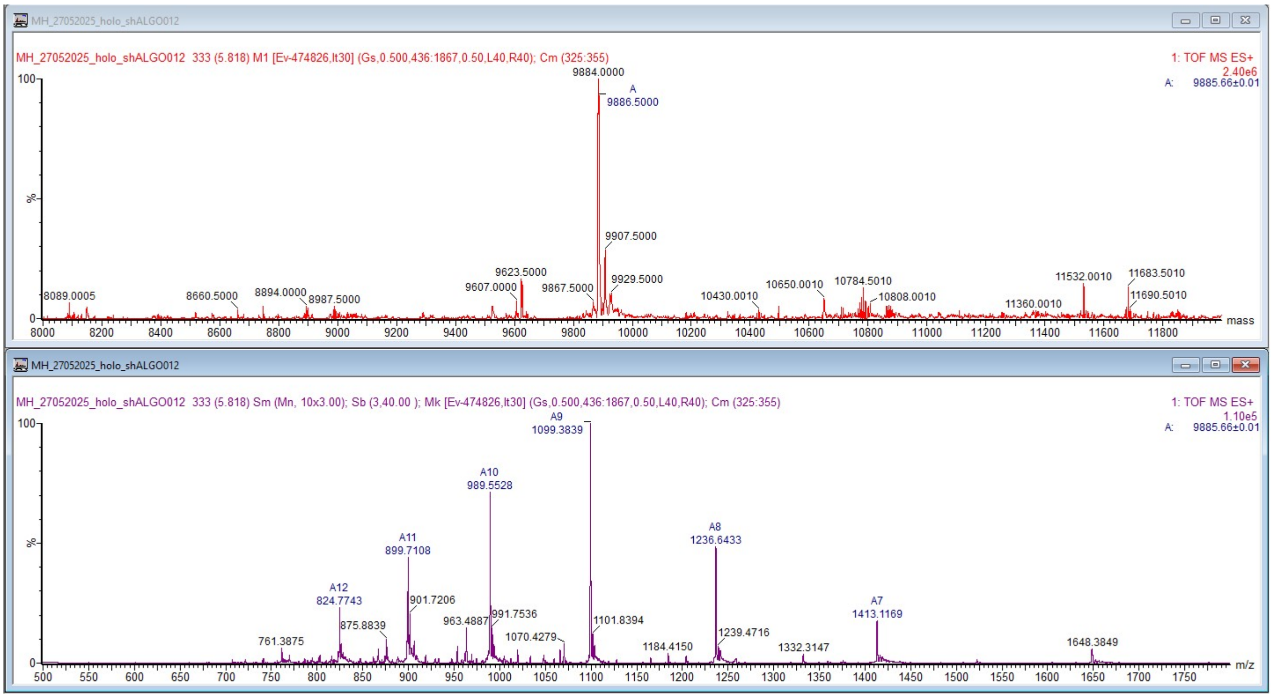

Fig. S33 *holo*-^{ch}ALGO-012 charge envelope.

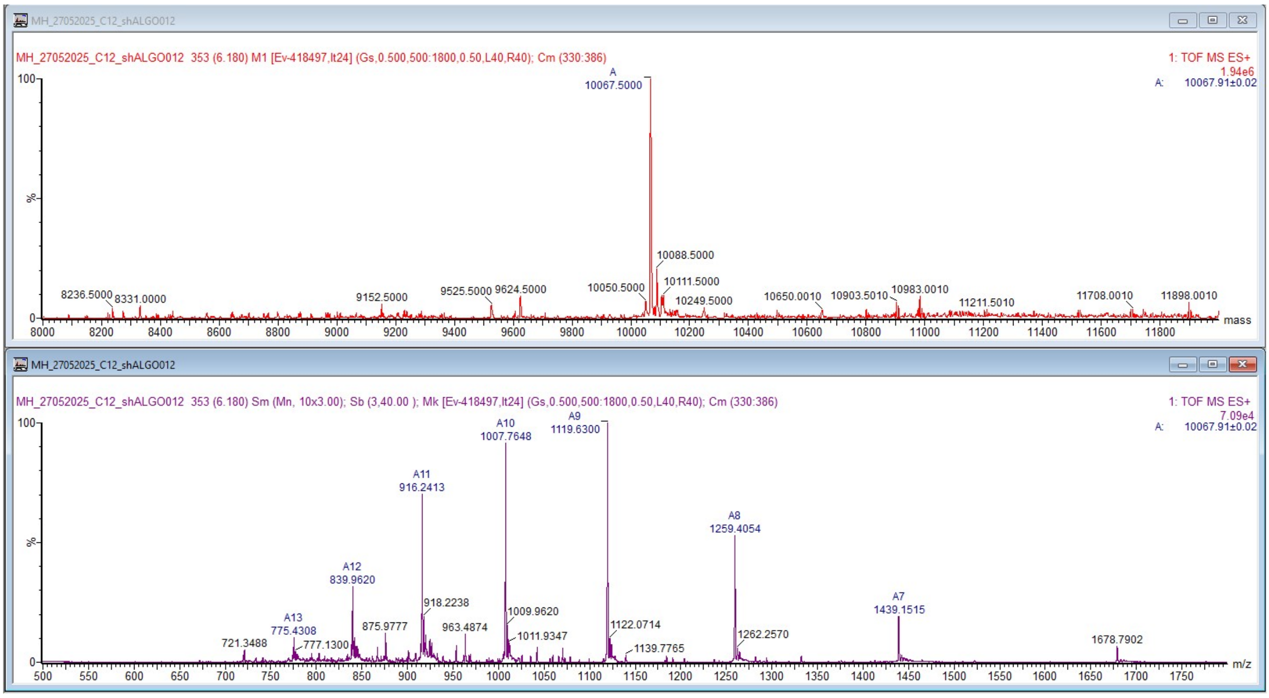

Fig. S34 C_{12}-^{ch}ALGO-012 charge envelope.

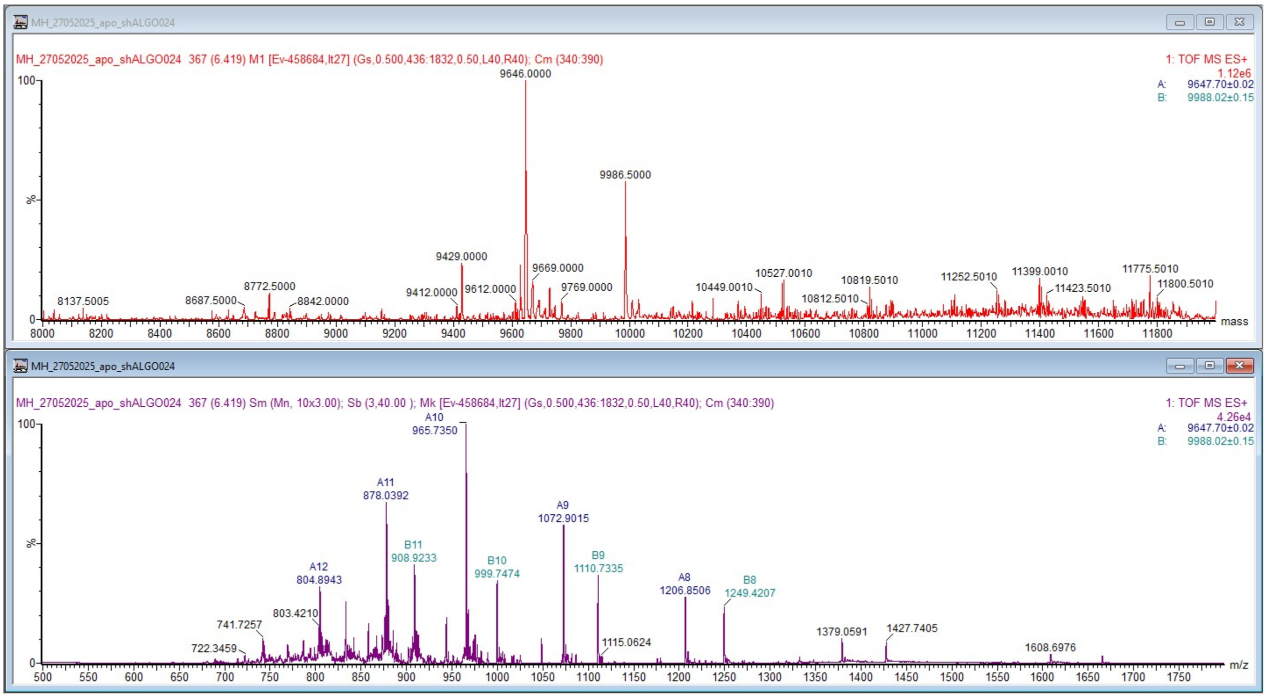

Fig. S35 *apo*-^{ch}ALGO-024 charge envelope.

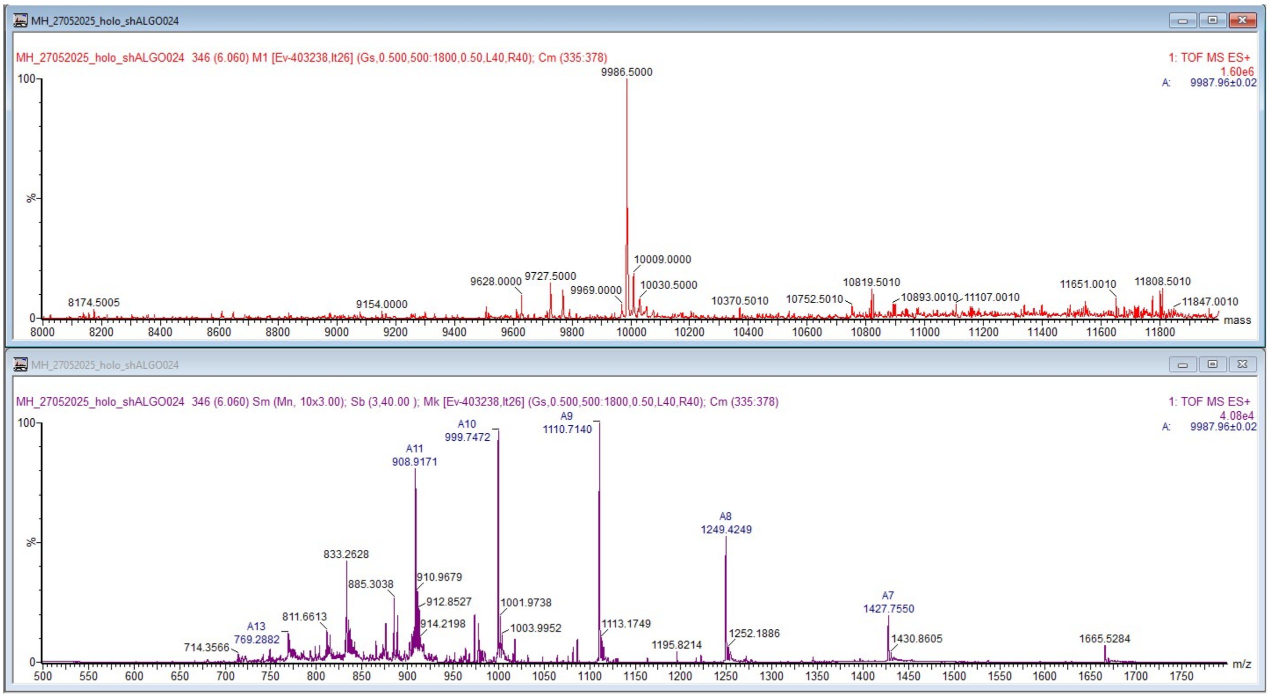

Fig. S36 *holo*-*Ec*AcpP charge envelope.

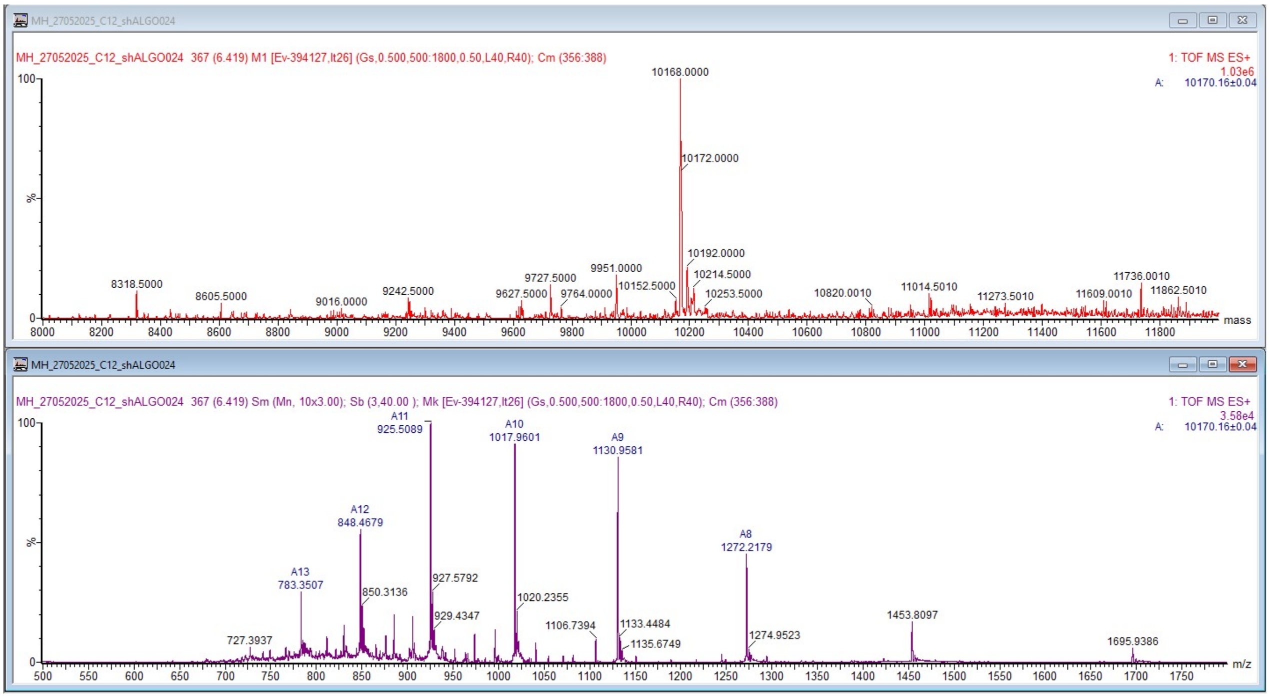

Fig. S37 C_{12}-^{ch}ALGO-024 charge envelope.

Structural modelling and MDS (Figs. S38-S47)

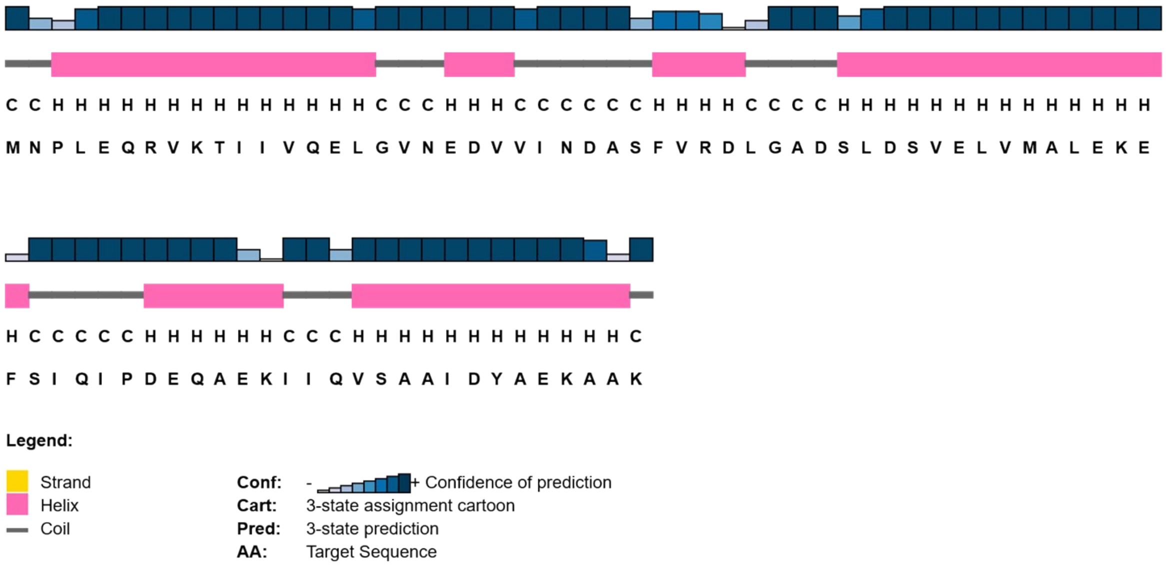

Fig. S38 PSIPRED secondary structure prediction from ALGO-055 primary sequence.

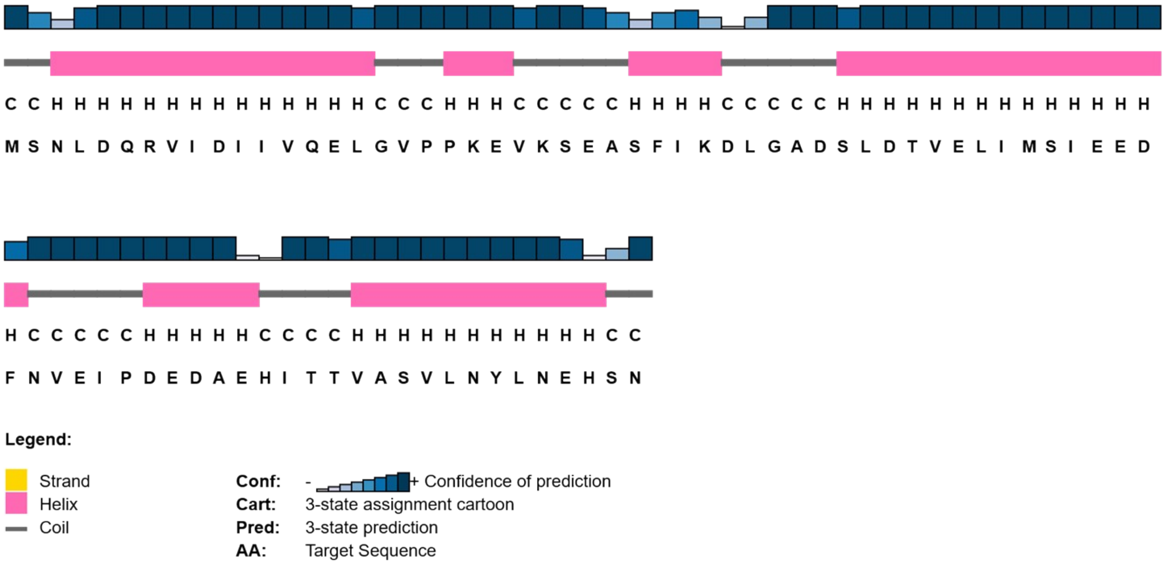

Fig. S39 PSIPRED secondary structure prediction from ALGO-059 primary sequence.

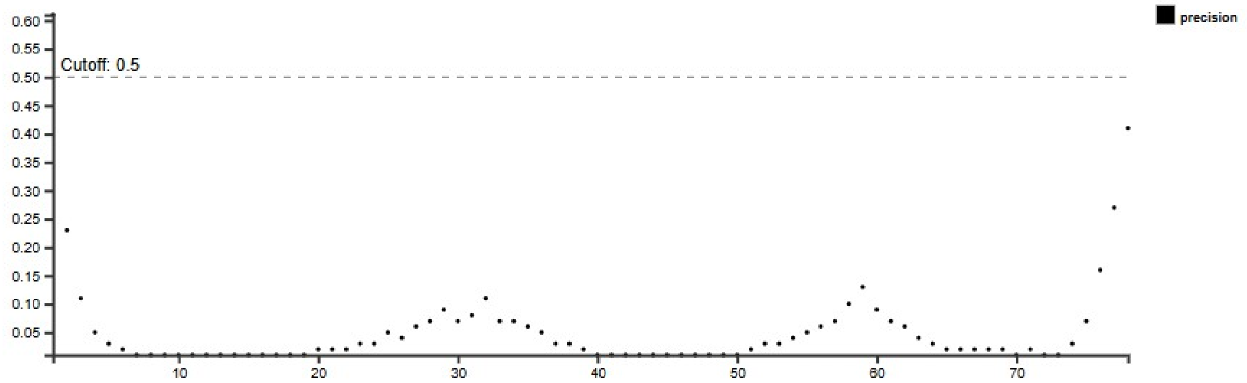

Fig. S40 DISOPRED3 disorder prediction from ALGO-059 primary sequence. Cut-off for protein disorder classification = 0.5.

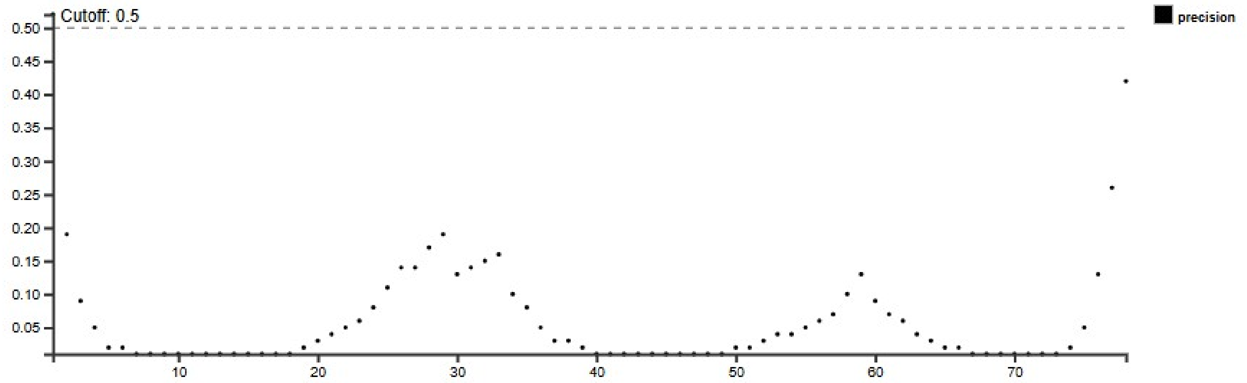

Fig. S41 DISOPRED3 disorder prediction from ALGO-059 primary sequence. Cut-off for protein disorder classification = 0.5.

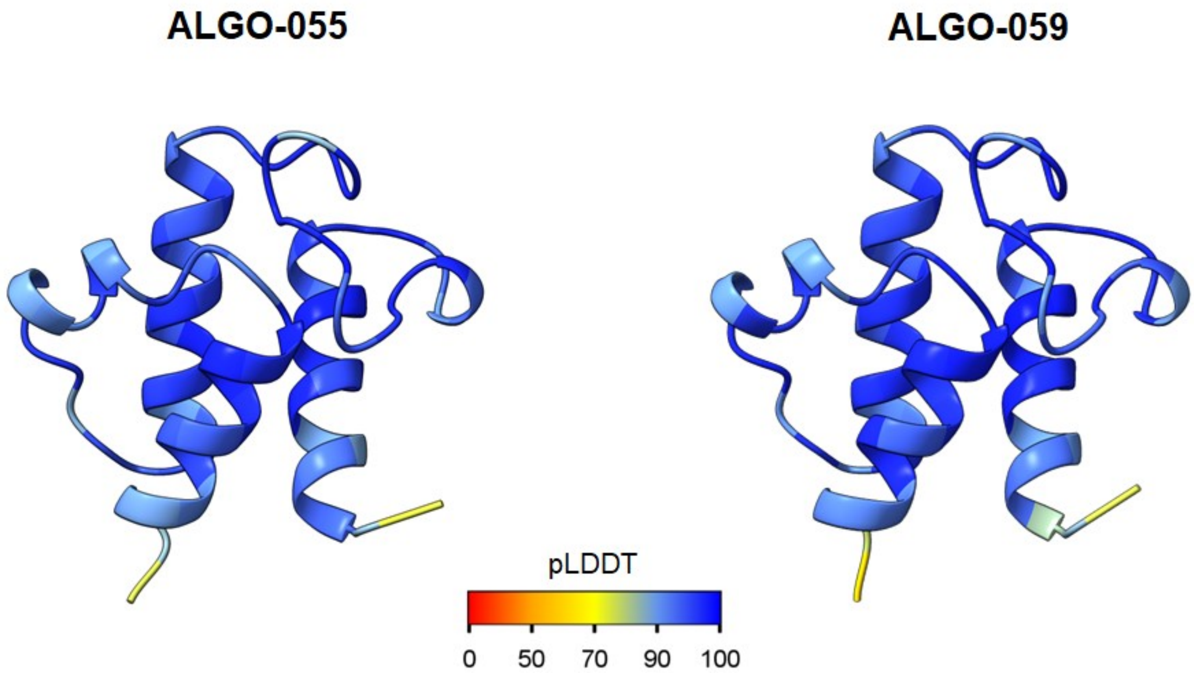

Fig. S42 AlphaFold3 predicted structural models of ALGO-055 and ALGO-059.

226

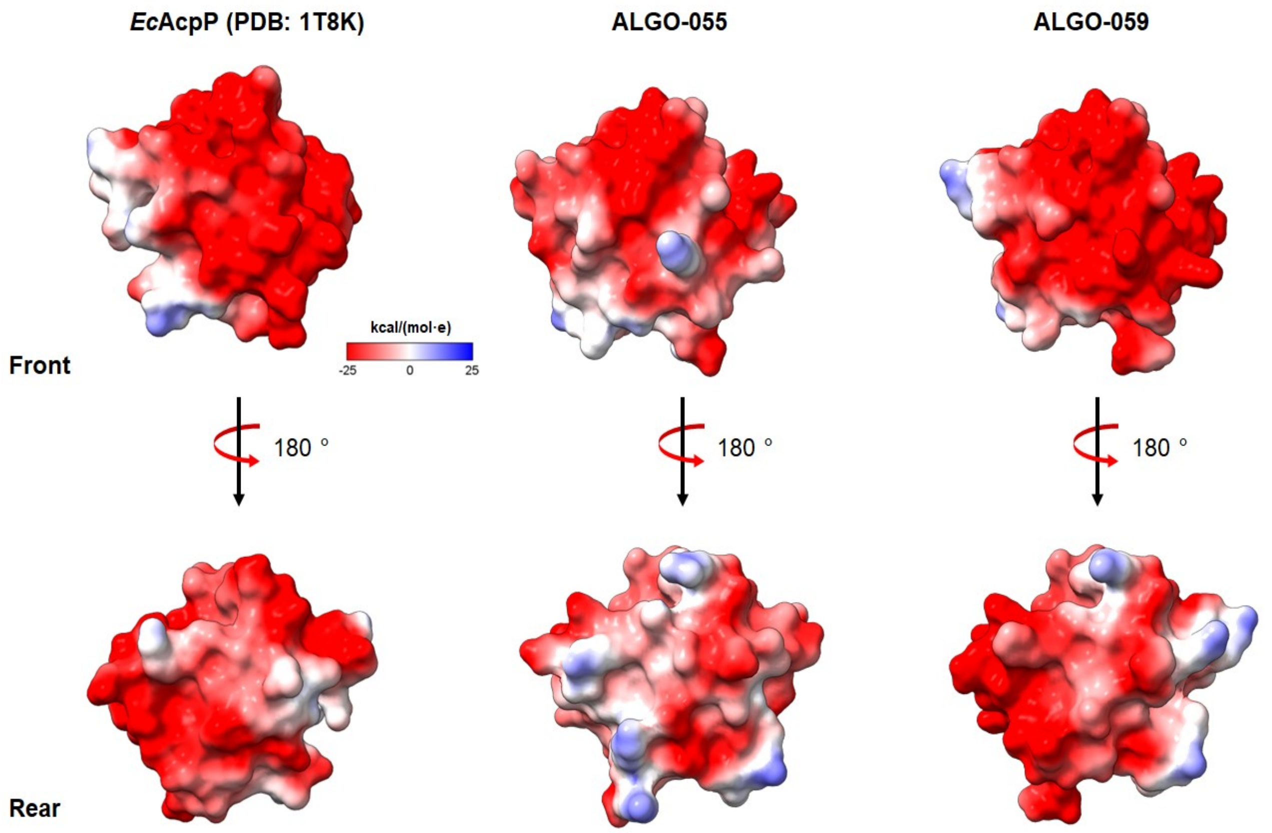

Fig. S43 Coulombic potential maps of *Ec*AcpP, alongisde AlphaFold3 models of ALGO-055 and ALGO059.

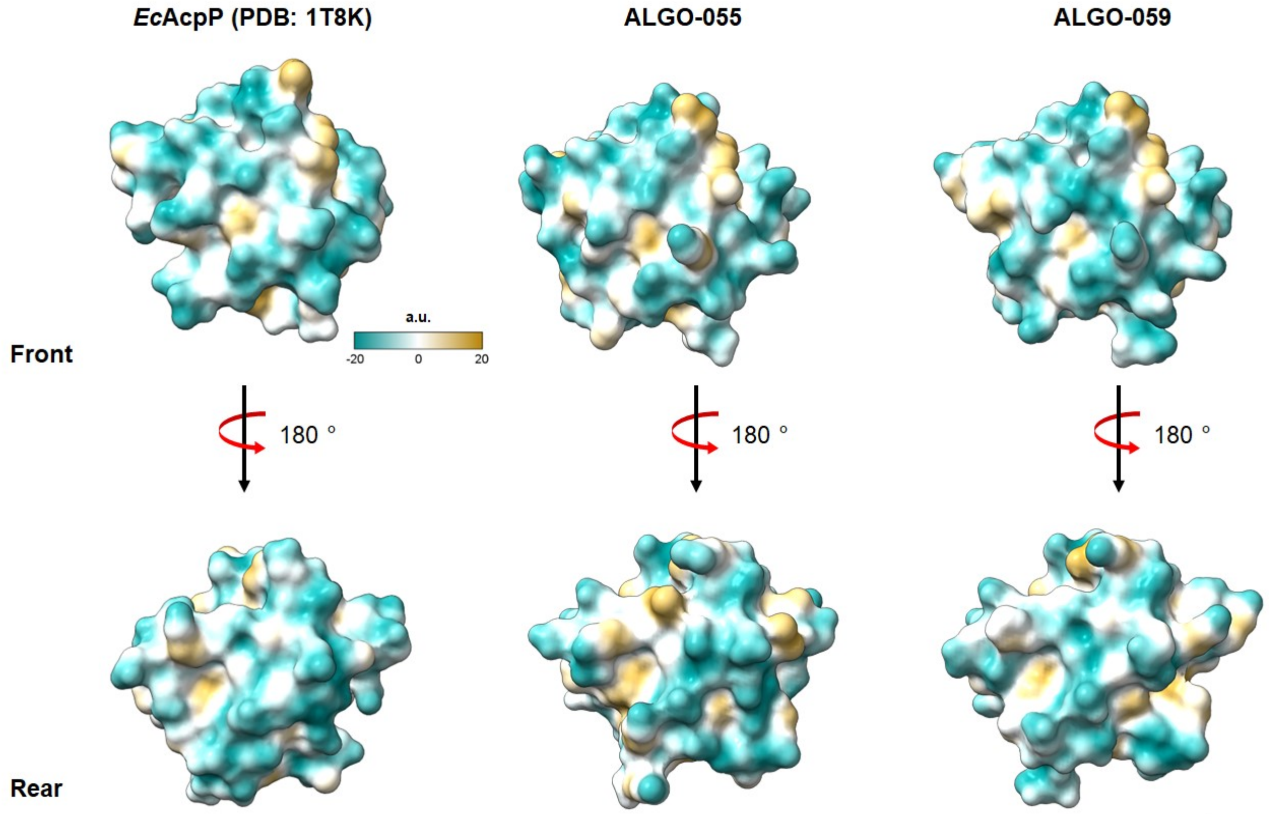

Fig. S44 Molecular lipophilicity potential maps of *Ec*AcpP, alongisde AlphaFold3 models of ALGO-055 and ALGO059.

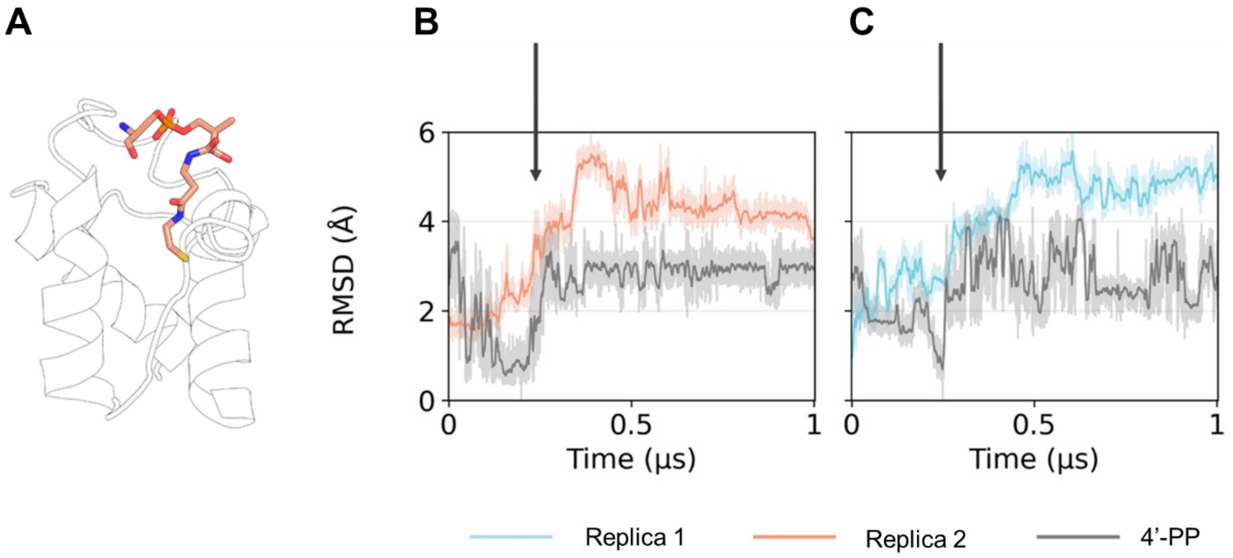

Fig. S45 Correlation between 4’-PP and protein RMSD in selected *holo*- ACP replicas. Arrows indicate the point of 4’-PP displacement from the ACP binding pocket. (A) A representative frame extracted from MD simulation, showing how the 4’-PP group positions itself inside the binding pocket. (B) *holo*-*Ec*AcpP, Replica 2 (C) *holo*-ALGO-059, Replica 1.

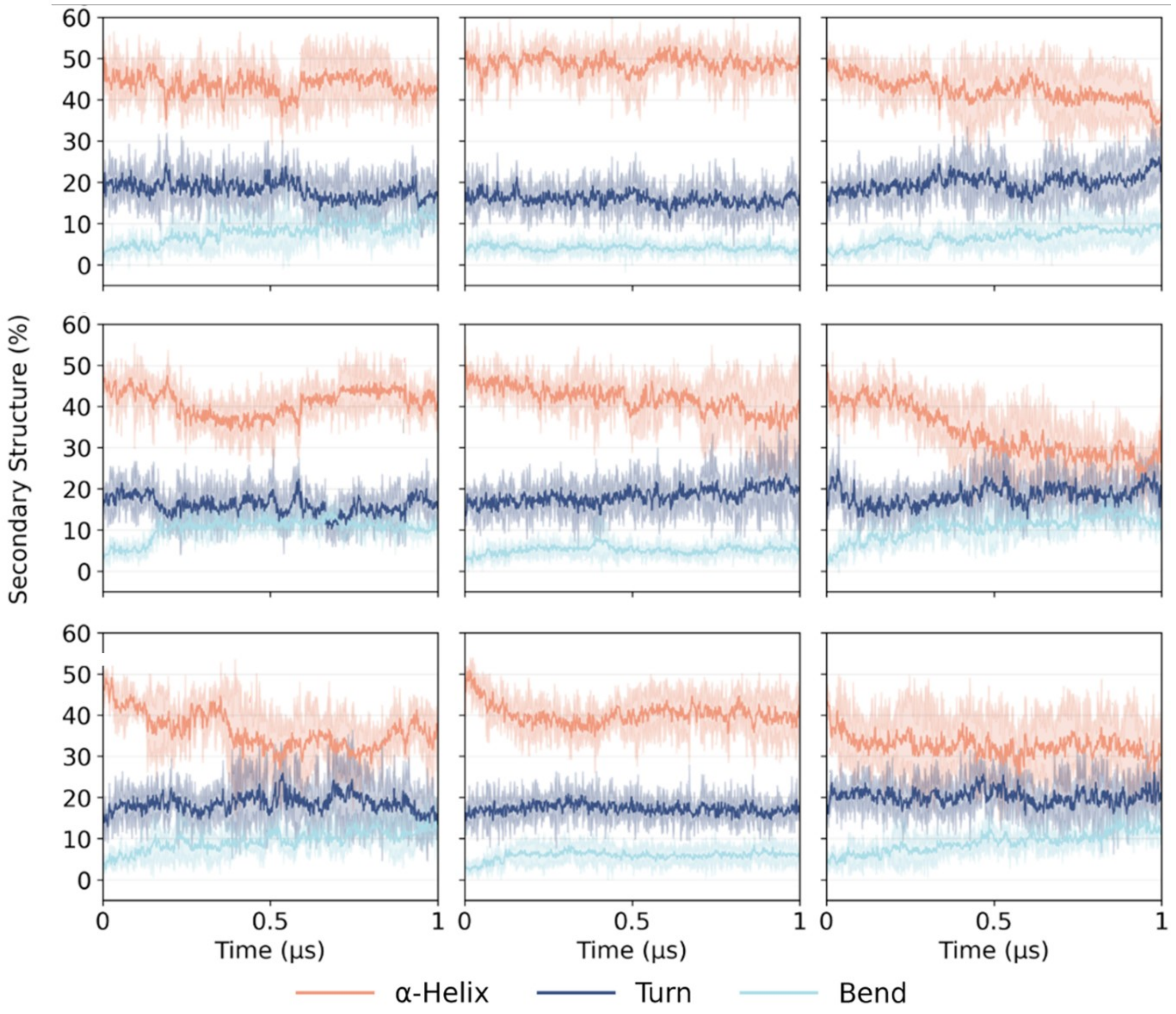

Fig. S46 Backbone DSSP profiles of MD trajectories. MD trajectories of *Ec*AcpP, ALGO-055, and ALGO-059 models were analysed in *apo*- (top), *holo*- (middle) and C_{12}-acylated (bottom) forms.

231

232

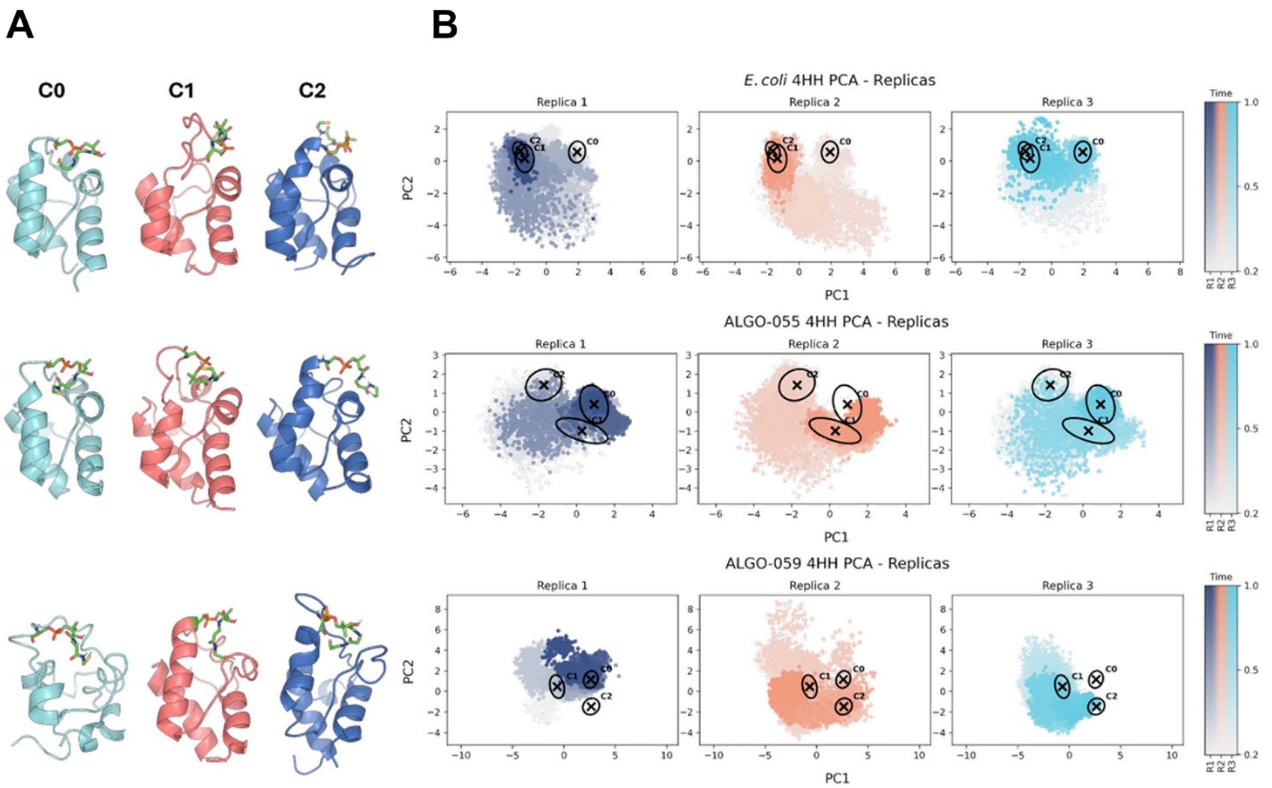

Fig. S47 Cluster analysis and PCA projections of *holo*-ACP trajectories. (A) Representative structures of the three main centroids (C0, C1, C2), clusters are marked as black circles. The 4HH moiety is represented as sticks in green. (B) PCA projections onto the PC1-PC2 plane for each replicate of the four ACP variants. Trajectories evolution is represented by colormaps. Clusters are marked as black circles.

Additional CD data (Fig. S48)

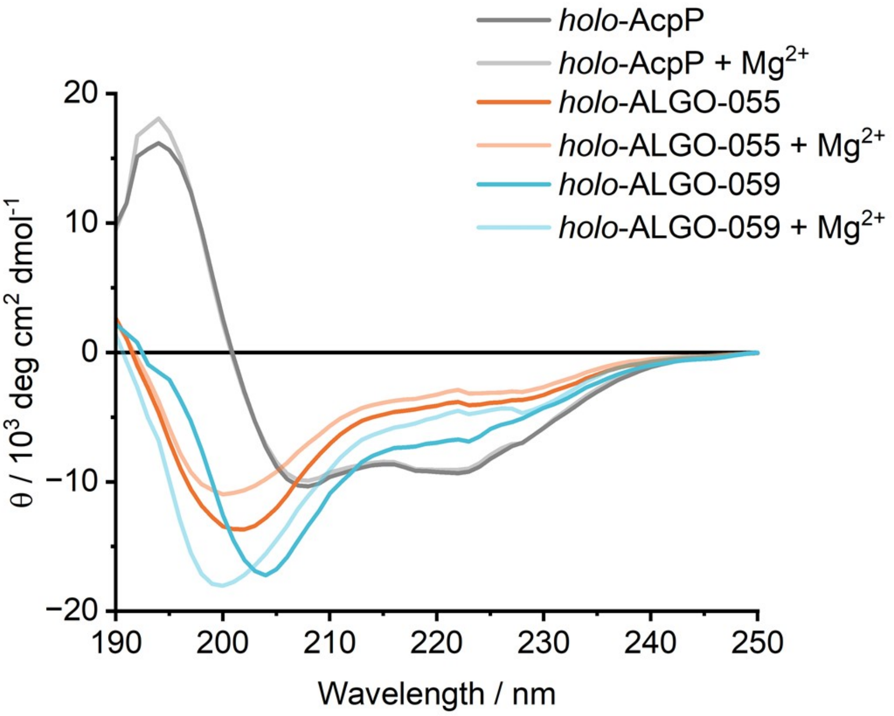

Fig. S48 CD spectra of *holo*- proteins before and after $MgCl_2$ supplementation.

Additional sequence data (Fig. S49)

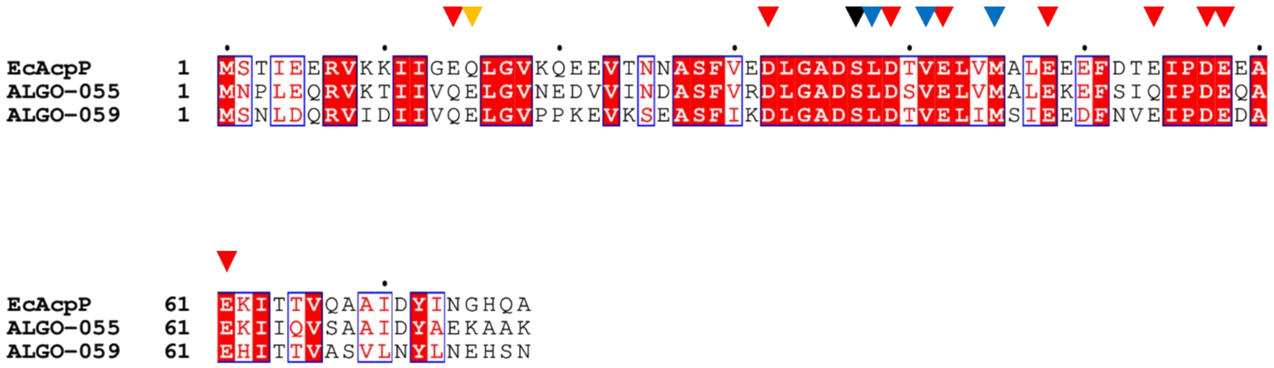

Fig. S49 MSA of *Ec*AcpP, ALGO-055 and ALGO-059, highlighting conserved contact residues. *Ec*AcpP residues involved in PPIs with *Ec*AcpS and *Vh*AasS, based on reported crystallographic and NMR data, are shown as triangles. Black triangle (position 37) highlights the position of the invariant serine. Red triangles show important acidic contact residues. Blue triangles highlight known hydrophobic contact residues that interact with *Vh*AasS. Yellow triangle (position 15) highlights Q→E variation in ALGO-055 and ALGO-059 that could still plausibly engage in PPIs.

Raw SDS-PAGE Images

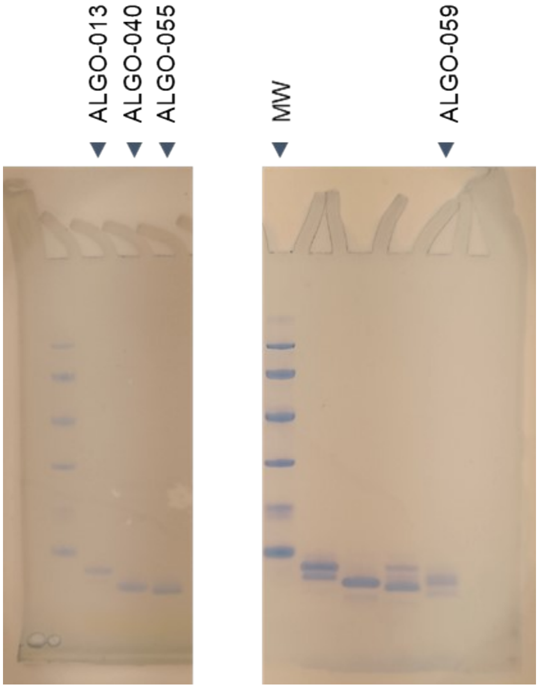

Raw SDS-PAGE gel photographs used for the composite image shown in Fig. 3B. Triangles indicate the lanes cropped.

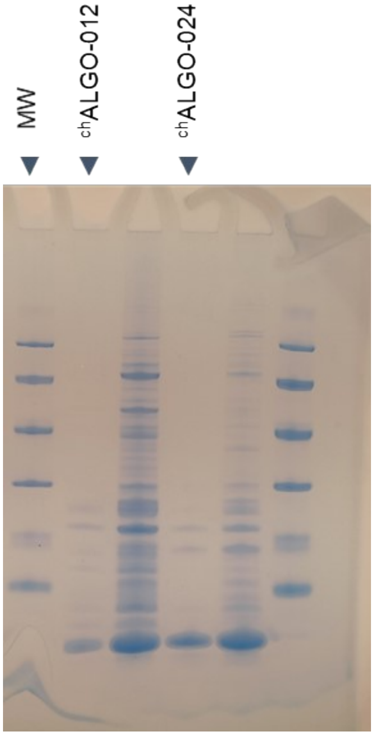

Raw SDS-PAGE gel photograph used for the image shown in Fig. 3G. Triangles indicate the lanes cropped.

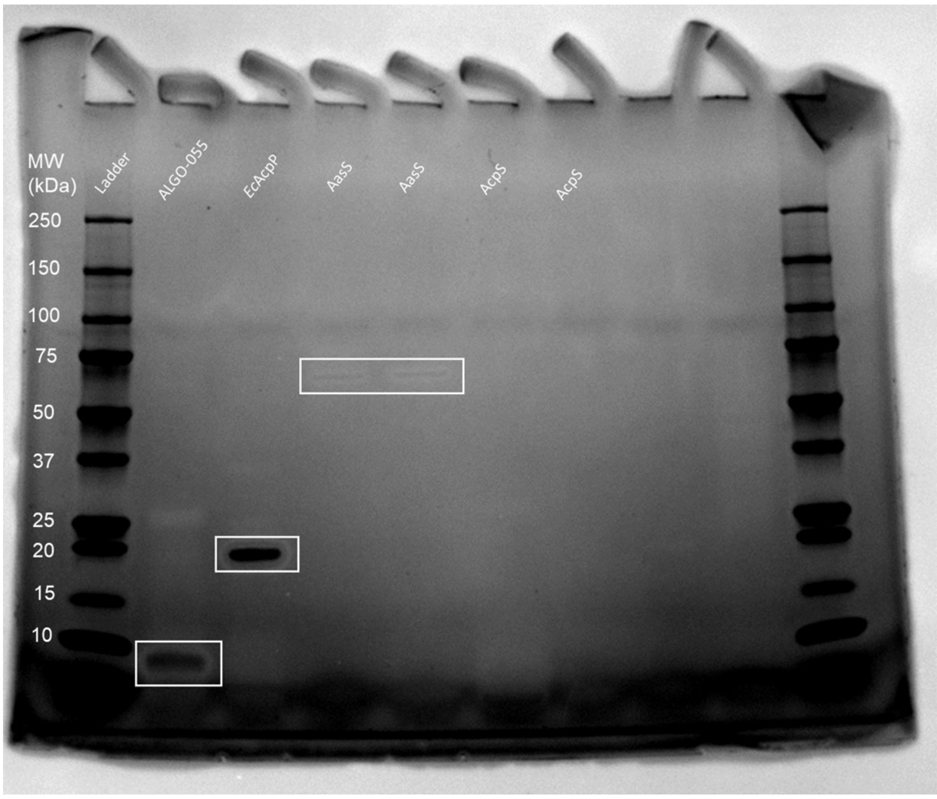

Raw SDS-PAGE gel photograph used for the image shown in Fig. S14A.

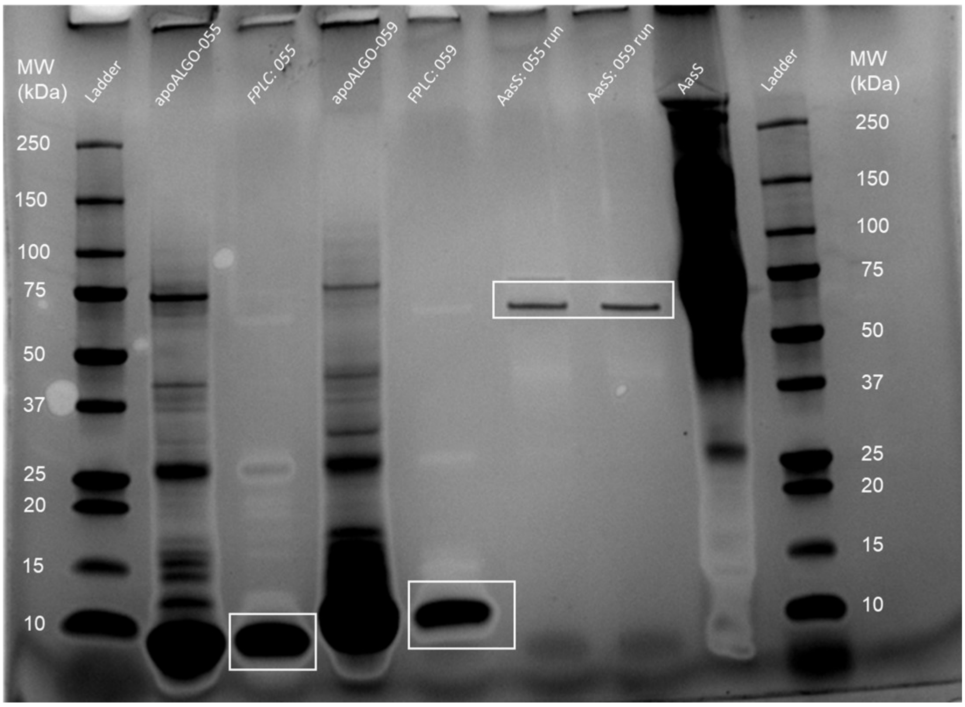

Raw SDS-PAGE gel photograph used for the image shown in Fig. S14B.

Plasmid Maps

ALGO013

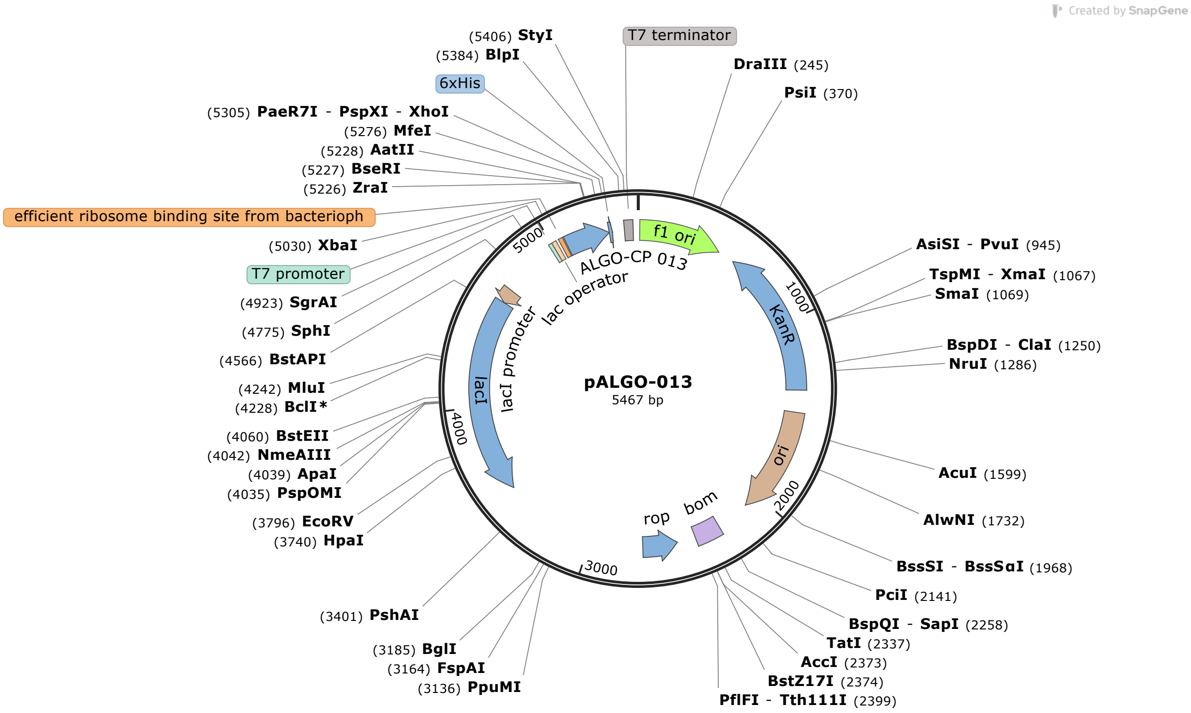

pAlgo-023

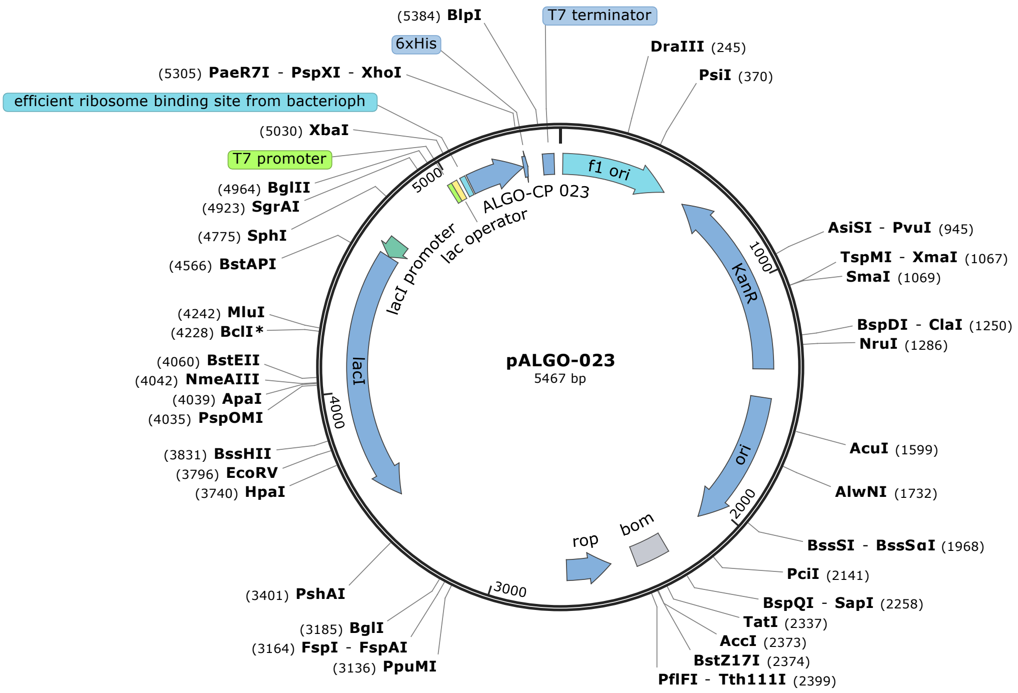
pALGO-023
5467 bp
(5384) BlpI
T7 terminator
6xHis
DraIII (245)
PsiI (370)
(5305) PaeR7I - PspXI - XhoI
efficient ribosome binding site from bacterioph
(5030) XbaI
T7 promoter
(4964) BglII
(4923) SgrAI
(4775) SphI
(4566) BstAPI
(4242) MluI
(4228) BclI*
(4060) BstEII
(4042) NmeAIII
(4039) ApaI
(4035) PspOMI
(3831) BssHII
(3796) EcoRV
(3740) HpaI
(3401) PshAI
(3185) BglI
(3164) FspI - FspAI
(3136) PpuMI
AsiSI - PvuI (945)
TspMI - XmaI (1067)
SmaI (1069)
BspDI - ClaI (1250)
NruI (1286)
AcuI (1599)
AlwNI (1732)
BssSI - BssSαI (1968)
PciI (2141)
BspQI - SapI (2258)
TatI (2337)
AccI (2373)
BstZ17I (2374)
PflFI - Tth111I (2399)
f1 ori
KanR
ori
rop
bom
lacI
lacI promoter
lac operator
ALGO-CP 023
1000
2000
3000
4000
5000

pALGO-040

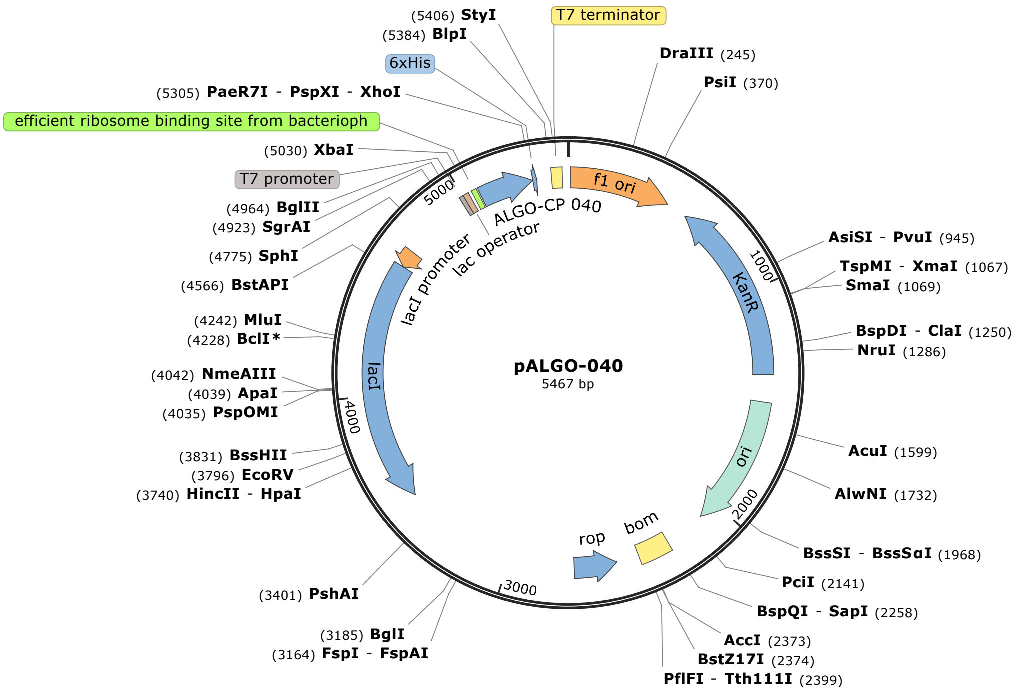

pALGO-040
5467 bp
(5406) StyI
(5384) BlpI
T7 terminator
DraIII (245)
PsiI (370)
6xHis
(5305) PaeR7I - PspXI - XhoI
efficient ribosome binding site from bacterioph
(5030) XbaI
T7 promoter
(4964) BglII
(4923) SgrAI
(4775) SphI
(4566) BstAPI
(4242) MluI
(4228) BclI*
(4042) NmeAIII
(4039) ApaI
(4035) PspOMI
(3831) BssHII
(3796) EcoRV
(3740) HincII - HpaI
(3401) PshAI
(3185) BglI
(3164) FspI - FspAI
AsiSI - PvuI (945)
TspMI - XmaI (1067)
SmaI (1069)
BspDI - ClaI (1250)
NruI (1286)
AcuI (1599)
AlwNI (1732)
BssSI - BssSαI (1968)
PciI (2141)
BspQI - SapI (2258)
AccI (2373)
BstZ17I (2374)
PflFI - Tth111I (2399)
f1 ori
ALGO-CP 040
lac operator
lacI promoter
KanR
lacI
ori
rop
bom
1000
2000
3000
4000
5000

ppALGO-044

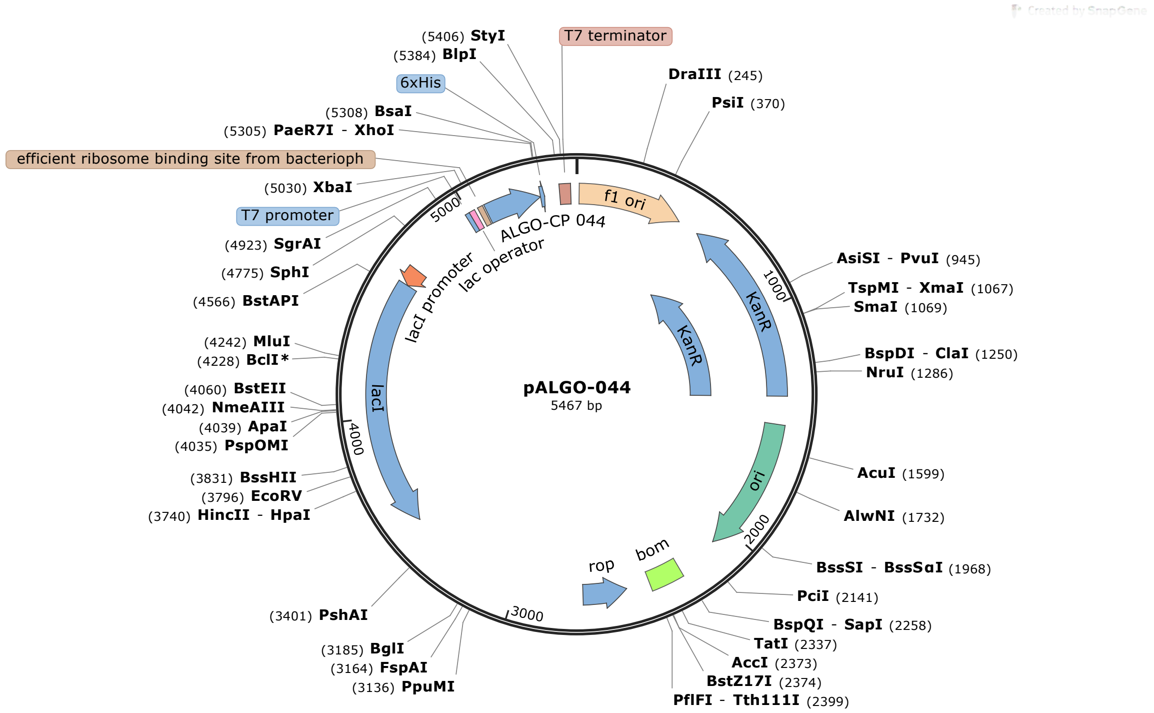
pALGO-044
5467 bp
(5406) StyI
(5384) BlpI
6xHis
(5308) BsaI
(5305) PaeR7I - XhoI
efficient ribosome binding site from bacterioph
(5030) XbaI
T7 promoter
(4923) SgrAI
(4775) SphI
(4566) BstAPI
(4242) MluI
(4228) BclI*
(4060) BstEII
(4042) NmeAIII
(4039) ApaI
(4035) PspOMI
(3831) BssHII
(3796) EcoRV
(3740) HincII - HpaI
(3401) PshAI
(3185) BglI
(3164) FspAI
(3136) PpuMI
T7 terminator
DraIII (245)
PsiI (370)
AsiSI - PvuI (945)
TspMI - XmaI (1067)
SmaI (1069)
BspDI - ClaI (1250)
NruI (1286)
AcuI (1599)
AlwNI (1732)
BssSI - BssSαI (1968)
PciI (2141)
BspQI - SapI (2258)
TatI (2337)
AccI (2373)
BstZ17I (2374)
PflFI - Tth111I (2399)
ALGO-CP 044
f1 ori
KanR
KanR
ori
bom
rop
lacI
lacI promoter
lac operator
1000
2000
3000
4000
5000

pALGO-055

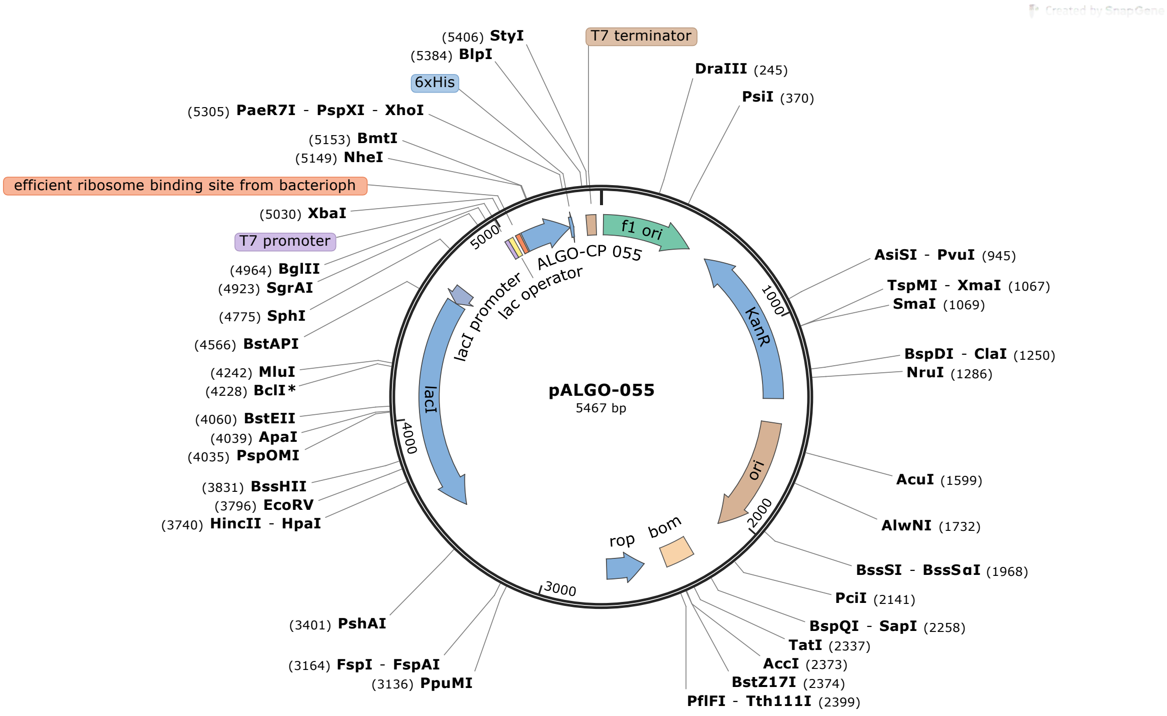
(5406) StyI
(5384) BlpI
6xHis
(5305) PaeR7I - PspXI - XhoI
(5153) BmtI
(5149) NheI
efficient ribosome binding site from bacterioph
(5030) XbaI
T7 promoter
(4964) BglII
(4923) SgrAI
(4775) SphI
(4566) BstAPI
(4242) MluI
(4228) BclI*
(4060) BstEII
(4039) ApaI
(4035) PspOMI
(3831) BssHII
(3796) EcoRV
(3740) HincII - HpaI
(3401) PshAI
(3164) FspI - FspAI
(3136) PpuMI
T7 terminator
DraIII (245)
PsiI (370)
AsiSI - PvuI (945)
TspMI - XmaI (1067)
SmaI (1069)
BspDI - ClaI (1250)
NruI (1286)
AcuI (1599)
AlwNI (1732)
BssSI - BssSαI (1968)
PciI (2141)
BspQI - SapI (2258)
TatI (2337)
AccI (2373)
BstZ17I (2374)
PflFI - Tth111I (2399)
f1 ori
ALGO-CP 055
lac operator
lacI promoter
lacI
KanR
ori
rop
bom
1000
2000
3000
4000
5000
pALGO-055
5467 bp

pALGO-057

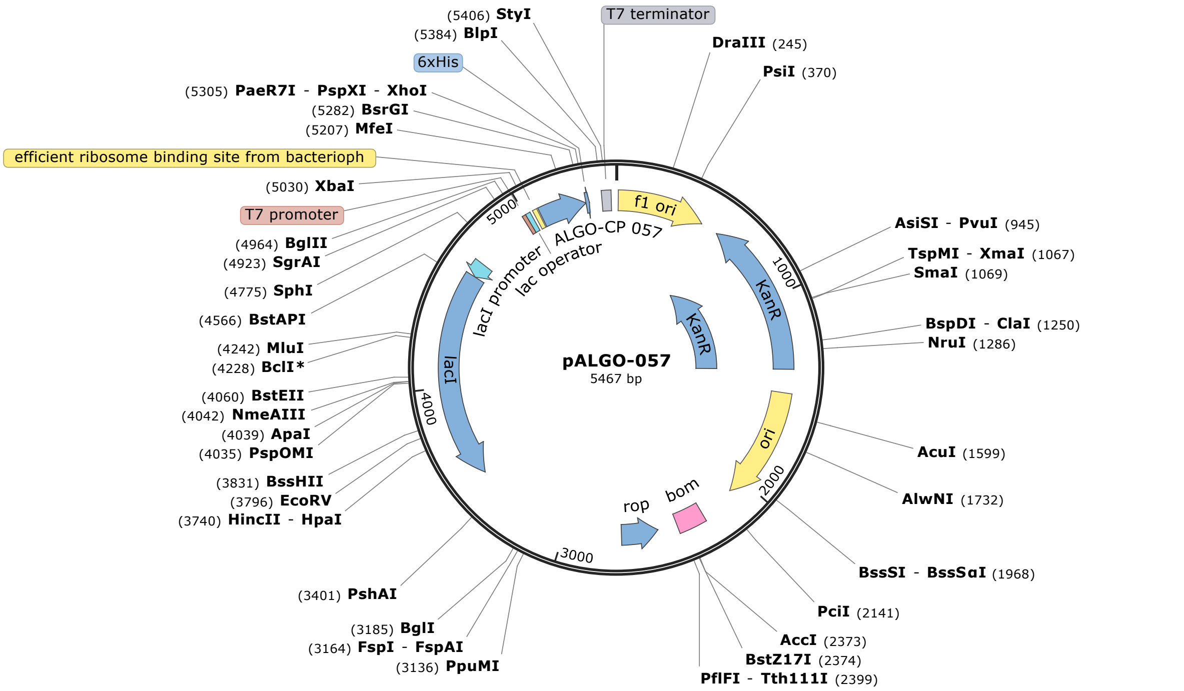
pALGO-057
5467 bp
(5406) StyI
(5384) BlpI
6xHis
(5305) PaeR7I - PspXI - XhoI
(5282) BsrGI
(5207) MfeI
efficient ribosome binding site from bacterioph
(5030) XbaI
T7 promoter
(4964) BglII
(4923) SgrAI
(4775) SphI
(4566) BstAPI
(4242) MluI
(4228) BclI*
(4060) BstEII
(4042) NmeAIII
(4039) ApaI
(4035) PspOMI
(3831) BssHII
(3796) EcoRV
(3740) HincII - HpaI
(3401) PshAI
(3185) BglI
(3164) FspI - FspAI
(3136) PpuMI
T7 terminator
DraIII (245)
PsiI (370)
AsiSI - PvuI (945)
TspMI - XmaI (1067)
SmaI (1069)
BspDI - ClaI (1250)
NruI (1286)
AcuI (1599)
AlwNI (1732)
BssSI - BssSαI (1968)
PciI (2141)
AccI (2373)
BstZ17I (2374)
PflFI - Tth111I (2399)
f1 ori
KanR
KanR
ori
bom
rop
lacI
lacI promoter
lac operator
ALGO-CP 057
1000
2000
3000
4000
5000

pALGO-059

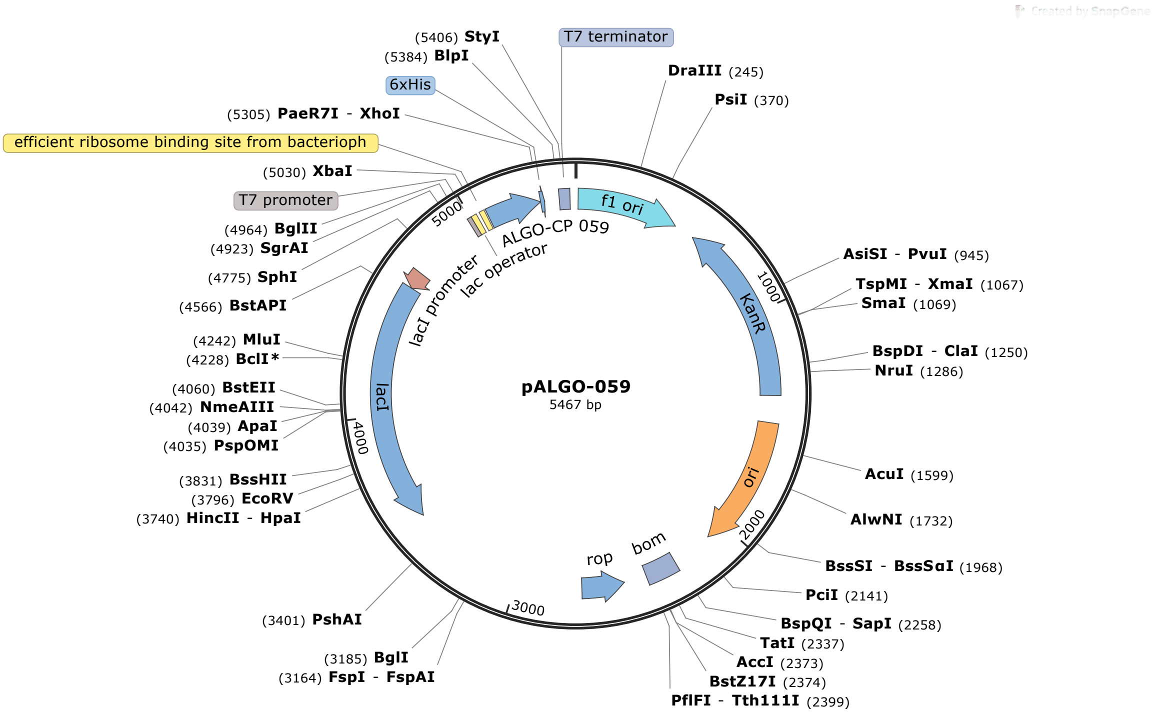

(5406) StyI
(5384) BlpI
6xHis
(5305) PaeR7I - XhoI
efficient ribosome binding site from bacterioph
(5030) XbaI
T7 promoter
(4964) BglII
(4923) SgrAI
(4775) SphI
(4566) BstAPI
(4242) MluI
(4228) BclI*
(4060) BstEII
(4042) NmeAIII
(4039) ApaI
(4035) PspOMI
(3831) BssHII
(3796) EcoRV
(3740) HincII - HpaI
(3401) PshAI
(3185) BglI
(3164) FspI - FspAI
T7 terminator
DraIII (245)
PsiI (370)
AsiSI - PvuI (945)
TspMI - XmaI (1067)
SmaI (1069)
BspDI - ClaI (1250)
NruI (1286)
AcuI (1599)
AlwNI (1732)
BssSI - BssSαI (1968)
PciI (2141)
BspQI - SapI (2258)
TatI (2337)
AccI (2373)
BstZ17I (2374)
PflFI - Tth111I (2399)
f1 ori
ALGO-CP 059
lac operator
lacI promoter
lacI
KanR
ori
rop
bom
pALGO-059
5467 bp
5000
1000
2000
3000
4000

pCHALGO-009

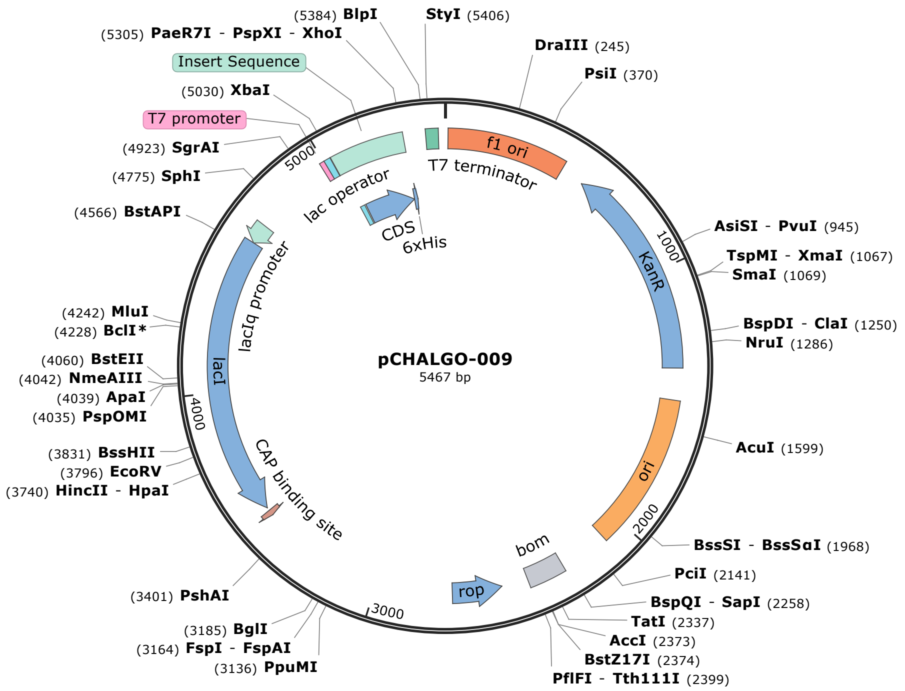

(5384) BlpI
StyI (5406)
(5305) PaeR7I - PspXI - XhoI
DraIII (245)
PsiI (370)
Insert Sequence
(5030) XbaI
T7 promoter
(4923) SgrAI
(4775) SphI
(4566) BstAPI
AsiSI - PvuI (945)
TspMI - XmaI (1067)
SmaI (1069)
BspDI - ClaI (1250)
NruI (1286)
(4242) MluI
(4228) BclI*
(4060) BstEII
(4042) NmeAIII
(4039) ApaI
(4035) PspOMI
AcuI (1599)
(3831) BssHII
(3796) EcoRV
(3740) HincII - HpaI
BssSI - BssSαI (1968)
PciI (2141)
(3401) PshAI
BspQI - SapI (2258)
TatI (2337)
AccI (2373)
BstZ17I (2374)
PflFI - Tth111I (2399)
(3185) BglI
(3164) FspI - FspAI
(3136) PpuMI
f1 ori
T7 terminator
lac operator
CDS
6xHis
KanR
lacIq promoter
lacI
pCHALGO-009
5467 bp
ori
CAP binding site
bom
rop
5000
1000
2000
3000
4000

pCHALGO-012

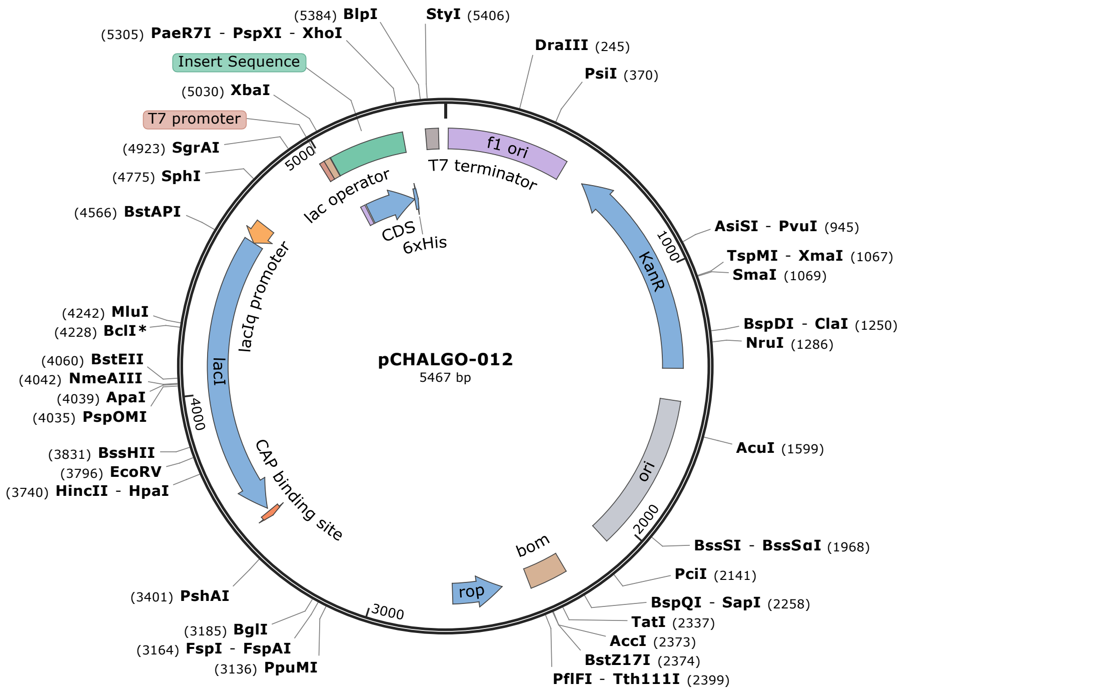
(5384) BlpI
StyI (5406)
(5305) PaeR7I - PspXI - XhoI
DraIII (245)
Insert Sequence
PsiI (370)
(5030) XbaI
T7 promoter
(4923) SgrAI
(4775) SphI
(4566) BstAPI
AsiSI - PvuI (945)
TspMI - XmaI (1067)
SmaI (1069)
(4242) MluI
(4228) BclI*
BspDI - ClaI (1250)
NruI (1286)
(4060) BstEII
(4042) NmeAIII
(4039) ApaI
(4035) PspOMI
AcuI (1599)
(3831) BssHII
(3796) EcoRV
(3740) HincII - HpaI
BssSI - BssSαI (1968)
PciI (2141)
(3401) PshAI
BspQI - SapI (2258)
TatI (2337)
(3185) BglI
(3164) FspI - FspAI
(3136) PpuMI
AccI (2373)
BstZ17I (2374)
PflFI - Tth111I (2399)
f1 ori
T7 terminator
lac operator
CDS
6xHis
KanR
lacIq promoter
lacI
CAP binding site
ori
bom
rop
pCHALGO-012
5467 bp
1000
2000
3000
4000
5000

pCHALGO-024

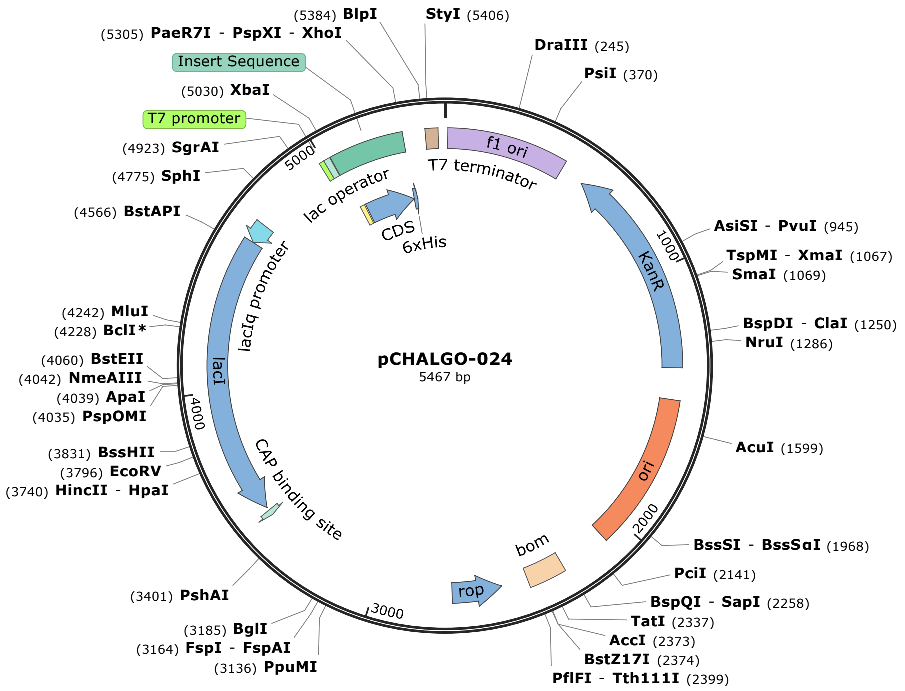
(5384) BlpI
StyI (5406)
(5305) PaeR7I - PspXI - XhoI
DraIII (245)
PsiI (370)
Insert Sequence
(5030) XbaI
T7 promoter
(4923) SgrAI
(4775) SphI
(4566) BstAPI
5000
f1 ori
T7 terminator
lac operator
CDS
6xHis
lacIq promoter
KanR
1000
AsiSI - PvuI (945)
TspMI - XmaI (1067)
SmaI (1069)
BspDI - ClaI (1250)
NruI (1286)
(4242) MluI
(4228) BclI*
(4060) BstEII
(4042) NmeAIII
(4039) ApaI
(4035) PspOMI
lacI
4000
pCHALGO-024
5467 bp
AcuI (1599)
ori
(3831) BssHII
(3796) EcoRV
(3740) HincII - HpaI
CAP binding site
2000
BssSI - BssSαI (1968)
bom
PciI (2141)
(3401) PshAI
rop
BspQI - SapI (2258)
3000
TatI (2337)
(3185) BglI
(3164) FspI - FspAI
(3136) PpuMI
AccI (2373)
BstZ17I (2374)
PflFI - Tth111I (2399)

pCHALGO-044

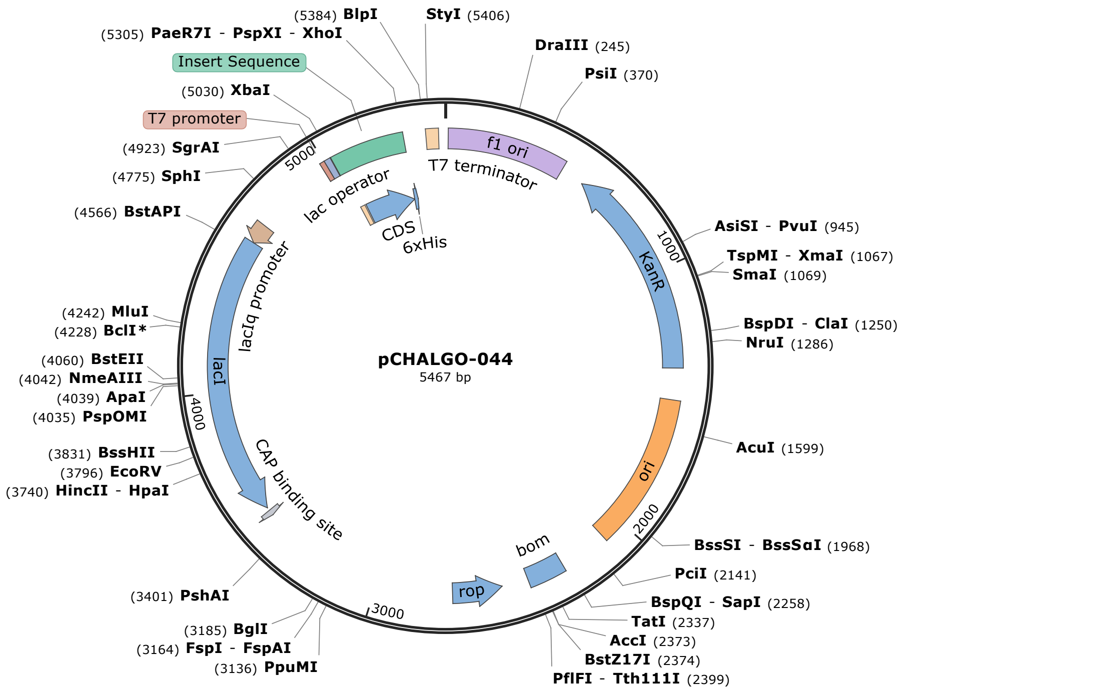

(5384) BlpI
StyI (5406)
(5305) PaeR7I - PspXI - XhoI
DraIII (245)
PsiI (370)
Insert Sequence
(5030) XbaI
T7 promoter
(4923) SgrAI
(4775) SphI
(4566) BstAPI
AsiSI - PvuI (945)
TspMI - XmaI (1067)
SmaI (1069)
(4242) MluI
(4228) BclI*
BspDI - ClaI (1250)
NruI (1286)
(4060) BstEII
(4042) NmeAIII
(4039) ApaI
(4035) PspOMI
AcuI (1599)
(3831) BssHII
(3796) EcoRV
(3740) HincII - HpaI
BssSI - BssSαI (1968)
PciI (2141)
(3401) PshAI
BspQI - SapI (2258)
TatI (2337)
(3185) BglI
AccI (2373)
(3164) FspI - FspAI
BstZ17I (2374)
(3136) PpuMI
PflFI - Tth111I (2399)
f1 ori
T7 terminator
lac operator
CDS
6xHis
KanR
lacIq promoter
lacI
CAP binding site
ori
bom
rop
pCHALGO-044
5467 bp
1000
2000
3000
4000
5000

pCHALGO-097

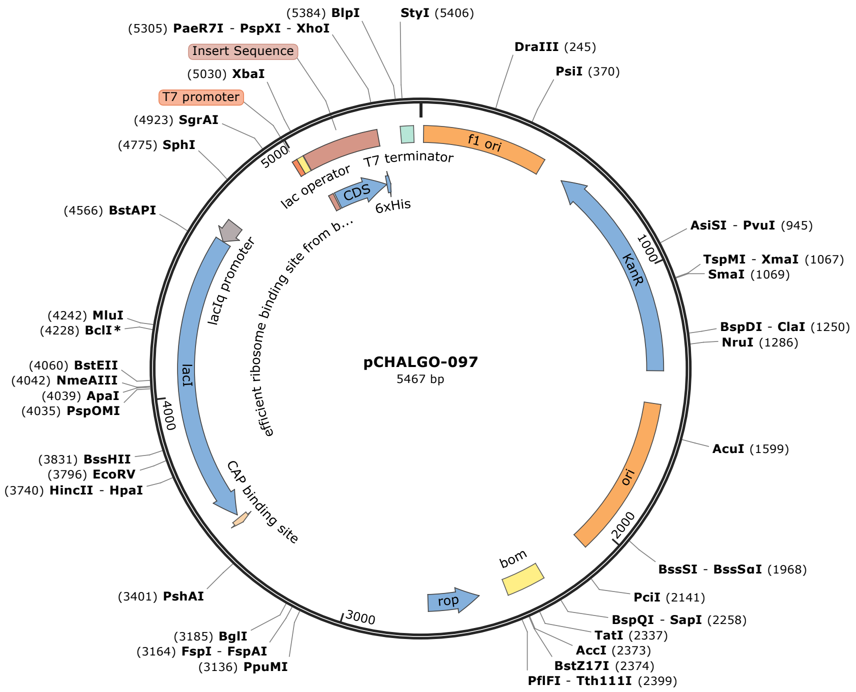
pCHALGO-097
5467 bp
(5384) BlpI
StyI (5406)
(5305) PaeR7I - PspXI - XhoI
Insert Sequence
(5030) XbaI
T7 promoter
(4923) SgrAI
(4775) SphI
(4566) BstAPI
(4242) MluI
(4228) BclI*
(4060) BstEII
(4042) NmeAIII
(4039) ApaI
(4035) PspOMI
(3831) BssHII
(3796) EcoRV
(3740) HincII - HpaI
(3401) PshAI
(3185) BglI
(3164) FspI - FspAI
(3136) PpuMI
DraIII (245)
PsiI (370)
AsiSI - PvuI (945)
TspMI - XmaI (1067)
SmaI (1069)
BspDI - ClaI (1250)
NruI (1286)
AcuI (1599)
BssSI - BssSαI (1968)
PciI (2141)
BspQI - SapI (2258)
TatI (2337)
AccI (2373)
BstZ17I (2374)
PflFI - Tth111I (2399)
f1 ori
T7 terminator
lac operator
CDS
6xHis
KanR
ori
bom
rop
lacIq promoter
lacI
efficient ribosome binding site from b...
CAP binding site
1000
2000
3000
4000
5000
